# Seafood traceability program design: Examination of the United States’ Seafood Import Monitoring Program

**DOI:** 10.1007/s13280-024-02075-8

**Published:** 2024-10-03

**Authors:** Andrew Steinkruger, Kailin Kroetz, Kaitlyn L. Malakoff, Jessica A. Gephart, Gloria Luque, Patrick Lee, Katrina Chicojay Moore, C. Josh Donlan

**Affiliations:** 1 Resources for the Future, 1616 P St NW, Washington, DC 20036 USA; 2 School of Sustainability, Arizona State University, 777 E University Dr, Tempe, AZ 85281 USA; 3 School of Aquatic and Fishery Sciences, University of Washington, 1122 NE Boat St Box 355020, Seattle, WA 98195 USA; 4 Advanced Conservation Strategies, PO Box 413, Midway, UT 84049 USA; 5 Cornell Lab of Ornithology, Cornell University, 159 Sapsucker Woods Road, Ithaca, NY 14850 USA

**Keywords:** Illegal, unreported, and unregulated, Mislabeling, Seafood import monitoring program, Traceability

## Abstract

The United States’ current Seafood Import Monitoring Program (SIMP) and a potential extension are undergoing review, yet quantitative evaluation of the current program is lacking. The SIMP is a traceability program aimed at reducing imports of seafood products that are of illegal, unreported, and unregulated (IUU) origin or associated with seafood fraud. We conducted a quantitative examination of the SIMP’s current scope and design by synthesizing publicly available trade data along with measures of IUU fishing and seafood mislabeling. We found prioritized shipments amounted to 33% of 2016 imported tonnage. The SIMP species groups had higher IUU scores and mislabeling rates relative to non-SIMP groups, but the difference was consistent with random prioritization suggesting potential benefits from program expansion. Furthermore, two-thirds of imported volume lacked a mislabeling rate and 5% lacked species information, underlining the urgent need for improved open-access data on globalized seafood supply chains.

**Supplementary Information:**

The online version contains supplementary material available at 10.1007/s13280-024-02075-8.

## Introduction

Post-implementation evaluation of the United States’ (U.S.) Seafood Import Monitoring Program (SIMP) is urgently needed as its future is uncertain, with implications for the SIMP and other potential programs. The SIMP has been controversial, and a proposed expansion was tabled in late 2023 (NOAA 2023). Instead, a longer and more public-facing process is underway to consider what expansion could entail and ways to enhance and strengthen the SIMP’s overall impact and effectiveness, with the goal of formulating recommendations on next steps by Fall 2024 (NOAA 2023; NOAA Fisheries 2024).

**Fig. 1:**
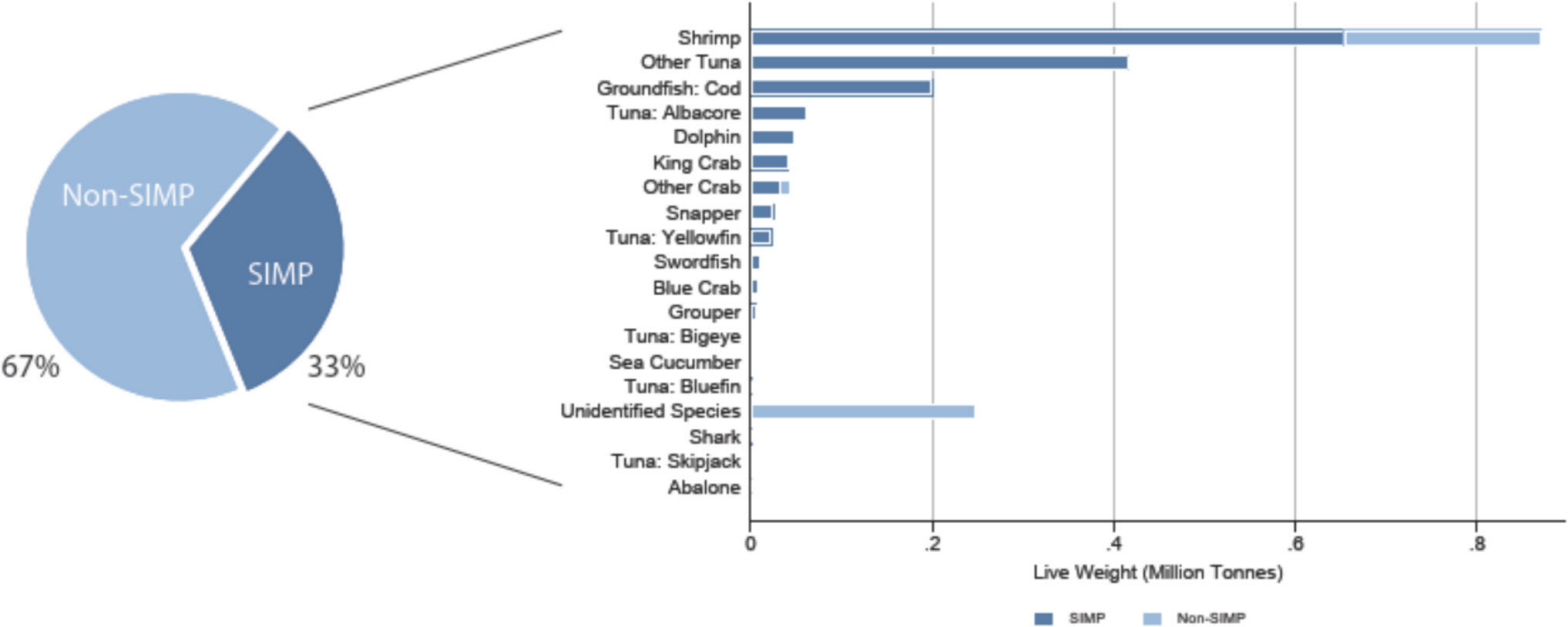
Estimated SIMP imports as a percentage of U.S. imports in 2016. SIMP and non-SIMP tonnage as a percentage of total live-weight imported (left). The SIMP final rule identified 13 prioritized groups (Atlantic Cod; Pacific Cod; Blue Crab; Red King Crab; Dolphinfish (Mahi Mahi); Grouper; Red Snapper; Sea Cucumber; Sharks; Swordfish; Tunas (Albacore, Bigeye, Skipjack, Yellowfin, and Bluefin); Abalone; Shrimp). We generally followed NMFS species groups but added blue crab for products with “Callinectes” in the product name to match the SIMP rule language (NMFS, 2016). Imports of NMFS species groups are presented in millions of metric tons live weight for all NMFS species groups that contain HTS codes covered by SIMP (right). The “Unidentified Species” group corresponds to a set of HTS codes that fall under groups with no identifiable species, e.g., “Fish NSPF,” or that include many species (see SI for a full list of HTS codes in this group)

The SIMP is a traceability system that unilaterally targets seafood at risk of being from illegal, unreported, and unregulated (IUU) fishing or associated with seafood fraud (NMFS 2016). With its implementation in January 2018, the SIMP imposed information-based controls forcing importers to register for a permit, report supply chain information from harvest to import on most prioritized shipments, retain records to be made available if requested for imports falling under the SIMP, and instituted selected audits of reported supply chain information for prioritized imports with the option to hold and physically inspect the imported product (NMFS 2016). The SIMP final rule identified 13 prioritized groups (Fig. 1) and used Harmonized Tariff Schedule (HTS) codes to identify products subject to required reporting and potential inspection (NMFS 2016). The initial list of species groups was determined by a Presidential Taskforce, but despite references to data and risk in rulemaking, the working group did not describe data and methods or summarize results related to risk of IUU and mislabeling (Presidential Task Force 2015).

Examination of the SIMP is needed to inform current policy decisions as well as to understand the potential for traceability programs to meet goals set by national and supranational governments worldwide (Bailey et al. 2016). As outlined in SALT (2021), SIMP builds on coverage of seafood traceability programs that have been implemented recently in the European Union and Japan, which together have global impacts on producing countries through seafood trade. Given uncertainty over the future of sustainability initiatives related to seafood (e.g. Roheim et al. (2018)), it is essential to examine these traceability initiatives as a tool to improve seafood sustainability.

Despite the SIMP’s coverage of a substantial portion of U.S. seafood imports (e.g., NOAA Fisheries 2024), there has been relatively little evaluation of the program. Current literature on the SIMP is primarily qualitative (He 2018; Willette and Cheng 2018) with one quantitative study examining market power and the potential for on-the-ground management changes in harvest locations for shrimp, king crab, and tuna (Fang and Asche 2021). Here we fill this gap conducting the first quantitative study systematically investigating SIMP performance in terms of scope and program design.

## Materials and methods

### Data

Our primary seafood trade database was downloaded through the National Marine Fisheries Service’s (NMFS) Commercial Fisheries Statistics (NMFS n.d.) and contained information on fishery products by country of origin (i.e., country in which a product was last “substantially transformed”), NMFS species group, 10-digit HTS code, and the associated product description (“product name”). We used data from 2016 to reflect information available during the rulemaking period and the set of HTS codes from the final SIMP rule to define HTS codes treated under the program (NMFS 2016). All trade tonnages were reported in kilograms raw weight, which we converted to estimated weight at harvest (“live weight”) in metric tons using product-specific conversion ratios from the European Market Observatory for Fisheries and Aquaculture Products (EUMOFA 2021) to achieve a standardized weight measure that is not tied to product form and instead aligned with fishery management (e.g., Kroetz et al. (2020)).

We also estimated import quantity attributable to aquaculture for each NMFS species group. To do this, we accessed production data by Aquatic Sciences and Fisheries Information System (ASFIS) species 3-alpha code for each country from the FishStatJ Production Database (FAO 2023). We assigned ASFIS 3-alpha codes to NMFS species groups using scientific taxa (Table S12), calculated the percentage of production attributable to aquaculture, and merged this data to our primary trade database. See Supplementary Information (SI) including Figure S1 for additional information on databases and linkages.

After constructing our trade database, we linked it to the best available quantitative information on IUU and seafood fraud. For our analysis of IUU, we used data from the International Trade Commission (ITC) (USITC 2021) to calculate a relative ranking of IUU risk from capture fisheries imported for each HTS code and country. Although the ITC data is from 2018 and therefore post-SIMP, to our knowledge it is the best available data, and we used it under the assumption that although the composition of U.S. imports may have changed with SIMP over time, the relative ranking of percent IUU imported by HTS and country combination would not have changed substantially in the first year of mandatory compliance. Another limitation is the ITC inclusion of forced labor in estimates of IUU, but this is likely correlated with IUU fishing which should not substantially influence relative IUU ranking. Additionally, for our measure of fraud, we used data on seafood mislabeling from 112 mislabeling studies in the U.S. to develop distributions of posterior modal mislabeling rates following a Bayesian meta-analysis approach in Luque and Donlan (2019). There was sufficient data to estimate rates for 16 species groups (“mislabeling species group”). See SI including Tables S3 and S8 for additional detail including NMFS species group IUU scores and mislabeling rates.

### Scope

Our analysis of scope focused on characterizing the coverage of SIMP relative to all U.S. imports as well as tracing imports back to their country of origin. We estimated the total tonnage and value for each prioritized species group, broken down by SIMP versus non-SIMP tonnage and value. We also explored the presence of products that are designated as “Other” or that have descriptions that do not specify the species or that we could not assign to a unique species group (see Table S10) and their SIMP status. We concluded our examination of scope by turning to distributional impacts, calculating aggregate and proportional exports of SIMP and non-SIMP products to the U.S. for each exporting country.

### Program design

We assessed the program’s design by comparing SIMP and non-SIMP species groups on metrics for both the IUU and seafood fraud program goals. In the few instances where a species group contained both SIMP and non-SIMP products, we broke the species group into two (a SIMP and non-SIMP group). Although the program includes several degrees of treatment (see SI for a full description), we define treated shipments as those with HTS codes included in the final rule because they must, at minimum, identify an AFSIS 3-alpha code and may be subject to the full SIMP documentation requirements. Recognizing that we have data on all imports (and therefore from an inferential statistics perspective we have the population) we began by generating descriptive statistics comparing the implementation outcome in terms of program objective indicators and showed the full distribution of scores and reported the weighted mean score.

We also used nonparametric statistical hypothesis tests to develop insight into the process of SIMP designation and contextualize the observed outcome. Specifically, we explored the performance of SIMP as implemented relative to a relevant alternative treatment approach: random assignment of SIMP status to species groups. We tested the null hypothesis that the observed difference in the mean SIMP and non-SIMP scores for the outcome (IUU or mislabeling) equals that under random assignment of SIMP status to species groups against the one-sided alternative that the observed difference in the mean outcome score between SIMP and non-SIMP products was greater than that under random assignment of species groups to SIMP. Initial data exploration revealed a lack of normality in the distribution of scores (Fig. 4), and therefore, we used a nonparametric permutation test (Good 2006), randomly assigning the SIMP designation to species groups and calculating the weighted mean for the SIMP species group and the non-SIMP species group outcome for 20 000 repetitions. This allowed us to construct a distribution of the difference between the mean SIMP and non-SIMP scores under the null, which we used to calculate a p value for the observed difference by calculating the percentage of the observations greater than the observed value. We used quantity imported (in live weight) as weights for both the IUU and fraud outcomes in our main analysis (robustness checks are summarized in the SI).

## Results

### Scope

SIMP HTS codes represented about 33% of 2016 U.S. imports by converted live weight, 35% by product weight, and 38% by import value (Fig. 1, Table S9). SIMP-covered shrimp outweighed SIMP representation within each species group except tuna by at least an order of magnitude (Fig. 1). Our calculations suggest that SIMP coverage is lower than the approximately 50% coverage of U.S. seafood imports publicly reported (Table S2).

**Fig. 2:**
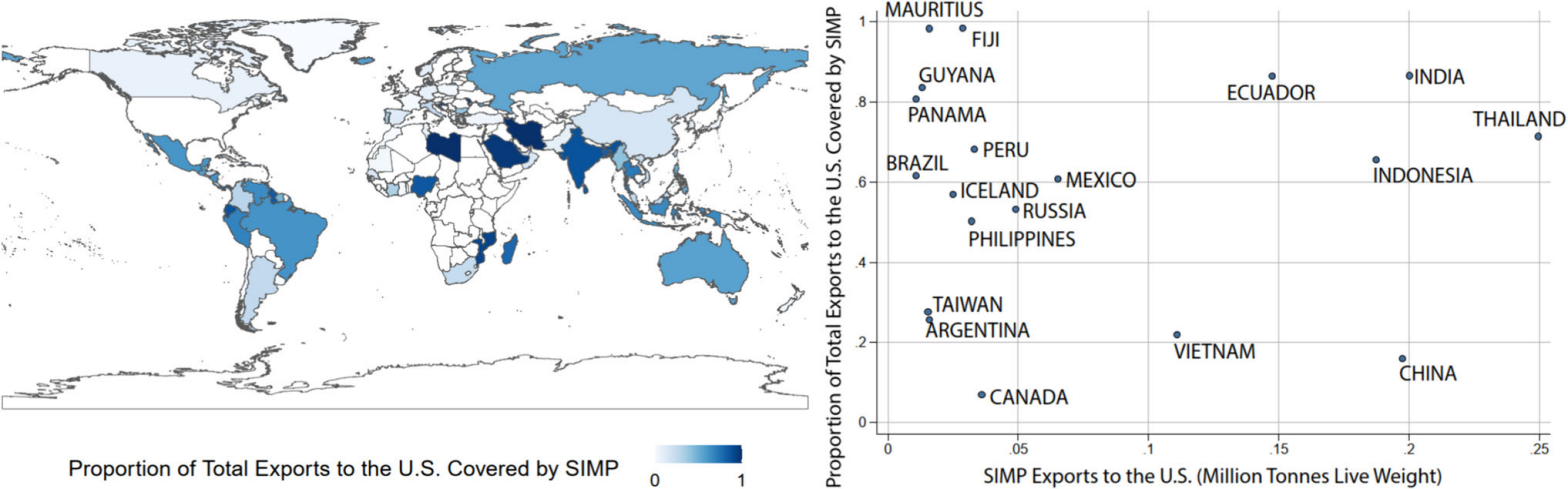
Aggregate estimated tonnage and proportion of exports to the U.S. covered by SIMP by country. The proportion of a country’s exports to the U.S. covered by SIMP is shown at left (with the exception of Tokelau and Reunion). Export tonnages and proportions of export tonnage of SIMP products to the U.S. are shown by country at right. Only exporters of more than 10 000 metric tons of SIMP products are shown, which together accounted for 94% by tonnage of imports of SIMP products

Across countries, the volume and value of SIMP products exported to the U.S. differed. The geographic distribution of exporters with high production and a large proportion of U.S. seafood exports falling under SIMP is shown in Fig. 2. Notably, high proportions of exports covered by the SIMP and high volume of SIMP shipments were concentrated in Ecuador, Indonesia, India, and Thailand, following the distribution of shrimp and tuna production. Often, a large proportion of exports are subject to SIMP in locations with low overall SIMP tonnages, for example, in Mauritius, Fiji, Guyana, and Panama (Fig. 2). However, diversified exports in countries with larger aquaculture and fishery sectors could explain the lower proportions of exports from some countries (e.g., China).

**Fig. 3:**
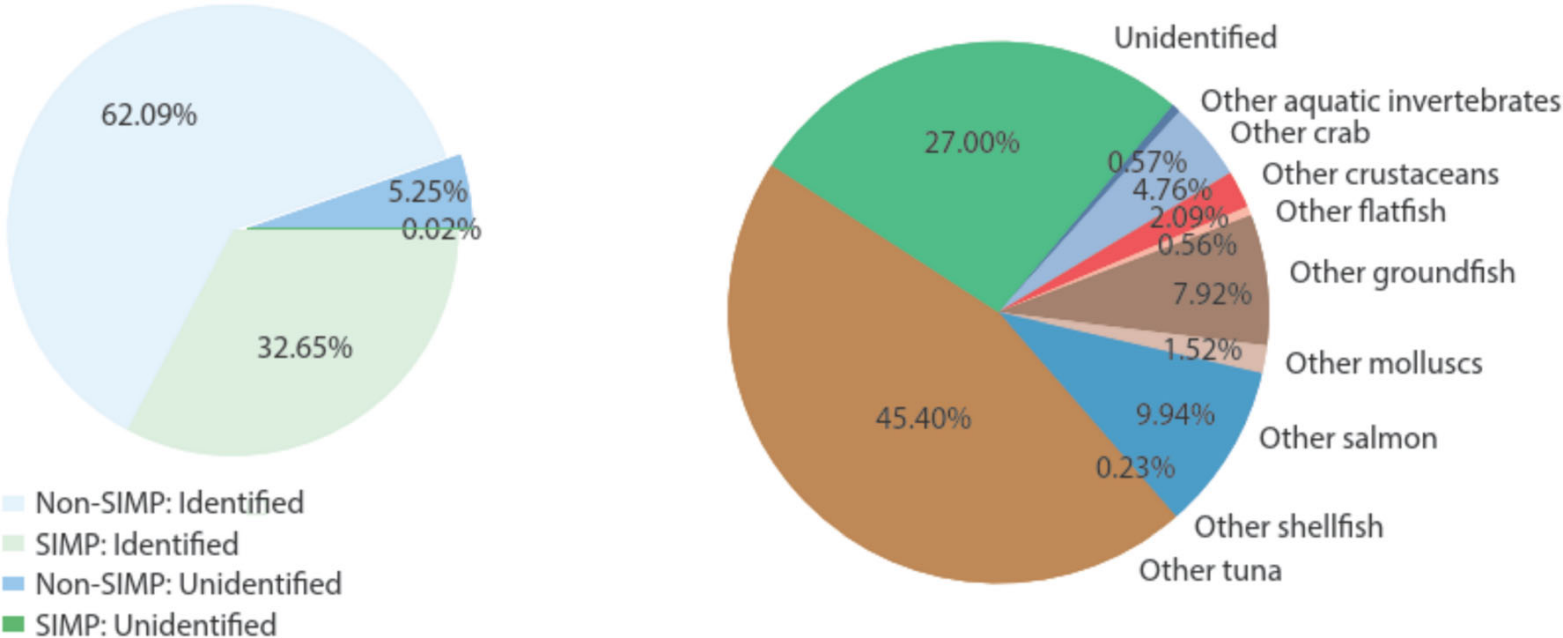
Presence of “Other” and “Unidentified” species in imports. The percentage of Unidentified species (those whose product descriptions do not specify the species or that we could not assign to a unique species group) in 2016 U.S. imports measured in live weight (left). A breakdown of the Other and Unidentified imports (right). Together the Other and Unidentified groups not covered by SIMP constitute 9.96% of imports (in live weight). A full list of HTS codes and their SIMP designation in the Other and Unidentified groups is in Table S10

Lastly, our empirical work highlights the presence of substantial quantities of other and unidentified species, many not subject to SIMP (Figs. 1 and 3; Table S10). On one hand, leaving these HTS codes with little or no species information out runs counter to the SIMP goals of “combat[ing] IUU fishing and seafood fraud” (NMFS, 2016). We note, however, that leakage to these less descriptive non-SIMP codes may be mitigated by the requirement to use “the most detailed and descriptive HTS code applicable to the product being entered” (see 19 CFR 141.90 and NMFS (2016)).

### Program design

We observe a higher weighted mean IUU score and mislabeling rate for SIMP versus non-SIMP species groups. Specifically, the observed weighted mean IUU score for SIMP species groups is 8.23, which is greater by 1.55 than the weighted mean for non-SIMP species groups. We observe the quantity-weighted mean species group mislabeling rate, for species groups for which a rate is available, of 0.20 which is 0.10 greater than the quantity-weighted mean mislabeling rate for non-SIMP species groups with rates available.

**Fig. 4:**
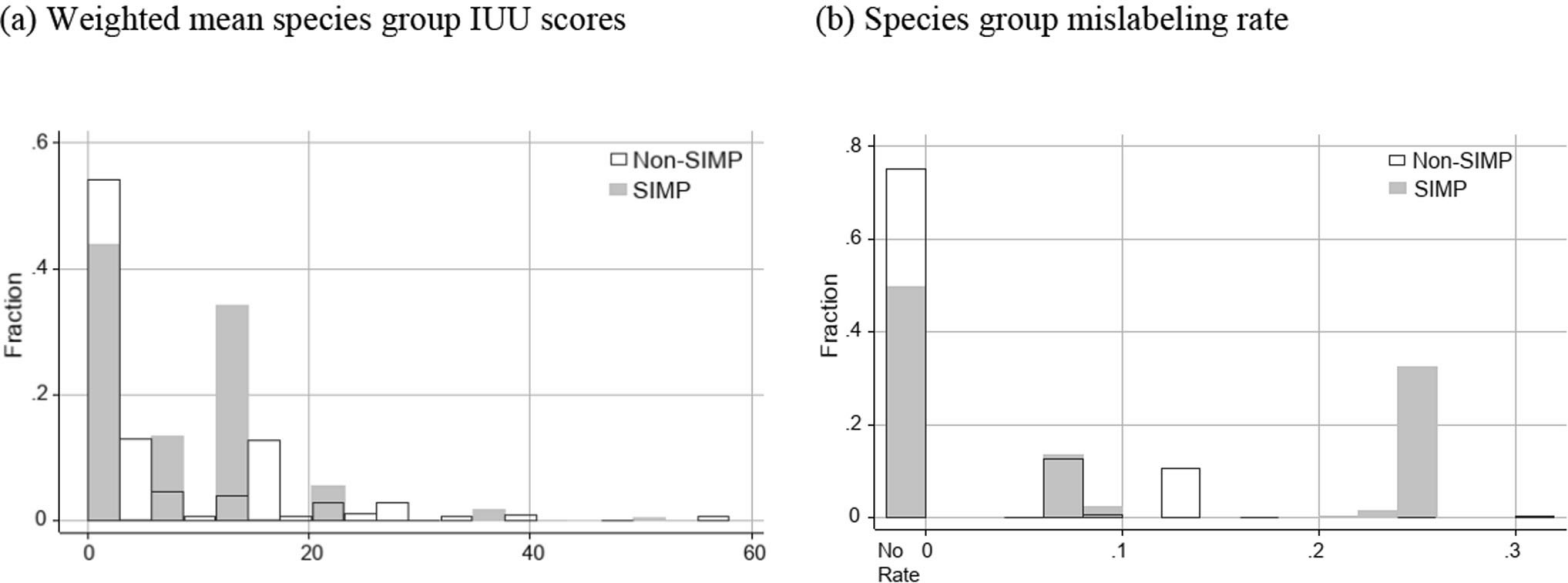
Species group IUU scores and mislabeling rates. Panel (a) contains a histogram of IUU scores for SIMP and non-SIMP species groups, weighted by live-weight tonnage. Our permutation test led to a failure to reject the null hypothesis that the observed difference between the tonnage-weighted SIMP and non-SIMP mean IUU scores (1.55) is equal to the difference if SIMP status had been randomly assigned to species groups (*p* value = 0.42). Panel (b) contains a histogram of observed mislabeling rates for SIMP and non-SIMP species groups, weighted by live-weight tonnage. Our permutation test led to a failure to reject the null hypothesis that the observed difference between the tonnage-weighted SIMP and non-SIMP mean mislabeling rates (0.10) is equal to the difference if SIMP status had been randomly assigned to species groups (*p* value = 0.20)

Our hypothesis testing of observed SIMP performance relative to a process where SIMP status was randomly assigned to species groups revealed performance in terms of targeting species groups was consistent with random assignment of SIMP prioritization. The IUU scores and mislabeling rates for SIMP species groups do not appear different from scores of non-SIMP products (Fig. 4a and b, respectively) and we were unable to reject the null hypothesis that the observed difference between SIMP and non-SIMP IUU scores equals that under random assignment (*p* value = 0.42 and *p* value 0.20, respectively). Additionally, 67% of imported tonnage has had insufficient testing done to estimate a mislabeling rate (Fig. 4b).

## Discussion

Our quantitative assessment of the scope and design of SIMP through a synthesis of the best available data on production, trade, IUU fishing, and fraud demonstrated (1) the program’s broad scope, and (2) that SIMP species groups had higher IUU scores and mislabeling rates relative to non-SIMP groups, but that the difference was consistent with random prioritization. Furthermore, our analysis revealed a substantial quantity of imported seafood without a known mislabeling rate and highlighted that about 5% of imports are not associated with a particular species.

With the current program and a potential expansion of the SIMP being evaluated, our work provides quantitative insights to this decision-making process. The hypothesis testing results are driven by species with relatively high import volume and IUU scores and mislabeling rates left out of the original SIMP rule. For IUU, these include octopus, sole, squid, swimming crab, and whiting (Table S3). Non-SIMP species groups with high mislabeling scores include several salmon species (Table S8). Apart from octopus and squid, none of these species were included in the (now withdrawn) proposed expansion (NOAA 2023). Our results for both IUU fishing and mislabeling suggest expanding program coverage to additional species groups with high IUU scores and/or mislabeling rates could increase program benefits. Additionally, product inspections create an opportunity to conduct testing to improve knowledge of mislabeling across species and within the supply chain.

Our process of conducting a synthesis of quantitative data to evaluate SIMP relative to its stated goals also provides support for calls to improve the quality and availability of data on seafood supply chains and advance research to inform policy for seafood sustainability (Cawthorn and Mariani 2017; Donlan and Luque 2019; Gephart et al. 2019; Kroetz et al. 2020). For example, gaps occur in mislabeling data for abalone, blue crab, dolphinfish, king crab, sea cucumber, shark, shrimp, and most non-SIMP products (Table S8). Additionally, because we do not have mislabeling information at all points along the supply chain, our analysis requires the assumption that products are not mislabeled at the point of import. However, it is possible the locations within the supply chain where mislabeling occurs could vary by species. We were also constrained by available production and trade data, having to make assumptions to estimate IUU and aquaculture quantities. Relatedly, the magnitude of changes along supply chains in realized import of mislabeled and IUU product remains unclear and future work should include evaluation of confidential audit and inspection data as well as assessment of the costs of these measures.

Here, we focused narrowly on the program goals of mislabeling and IUU, but traceability programs can adopt broader goals and/or have impacts beyond stated goals. Further data, discussion, and analysis could provide a broader understanding of other potential social and environmental impacts of the SIMP as is occurring around the potential expansion (NOAA 2023) and contribute to a broader discussion around traceability as a means of improving supply chain sustainability.

## Supplementary Information

Below is the link to the electronic supplementary material.

Supplementary file1 (PDF 3366 KB)

## Data Availability

All data and code used to conduct the analysis for the current study are available in the following GitHub repository: https://github.com/kaitlyn-c-lee/seafood-traceability-design.git.
