# Supplementary material for: Seafood traceability program design: Examination of the United States’ Seafood Import Monitoring Program
Source: Ambio. 2024 Oct 3;54(2):168–74. doi: 10.1007/s13280-024-02075-8 (PMC11662096; doi:10.1007/s13280-024-02075-8)
Supplement: Supplementary file 1 — Supplementary file1 (PDF 3366 KB) [file 13280_2024_2075_MOESM1_ESM.pdf]

Ambio

Supplementary Information
This supplementary information has not been peer reviewed.

Title: **Seafood Traceability Program Design: Examination of the United States' Seafood Import Monitoring Program**

Table of Contents

Terminology 2
SIMP Coverage 3
Database Construction 4
Trade Data 5
Percent Aquaculture Estimates 6
SIMP Status 7
Illegal, Unregulated, and Unreported (IUU) Fishing Indicators 8
Fraud Indicator: Seafood Mislabeling Rates Meta-Analysis 16
Robustness checks 30
Detailed Summary Statistics 32
Detailed Data Tables 38
References 126

Terminology

We combine data from multiple sources to construct the database we use for our analysis. Noting that multiple sources use the term "product" to refer to different things. We fix terminology in Table S1.

Table S1: Terminology used in the paper and SI.

Long-form description	*Abbreviated terminology used in the paper*
Reported 10-digit Harmonized Tariff Schedule (HTS) code	HTS code
HTS code product description	Product/HTS product/product name
FAO 3-alpha code	ASFIS species
Species group from the NMFS trade data (NMFS n.d.)	NMFS species group
Species groups listed as prioritized species groups in the SIMP Final Rule (NMFS 2016b)	SIMP prioritized species group
Seafood product categories for which mislabeling rates are estimated	Mislabeling species group
Individual import record by country of origin and 10-digit HTS code (NMFS n.d.)	Shipment
Country in which a product was last "substantially transformed" (19 CFR Part 177),	Country of origin

SIMP Coverage

Table S2 provides a non-exhaustive list of government documents that summarize imports covered via the SIMP, and the associated volumes, values, and/or percentages of volumes or values covered.

Table S2: A non-exhaustive list of government documents that summarize SIMP import coverage.

Report citation	*SIMP import value reported (USD)*	*Percent of all seafood import value*	*SIMP import volume reported (kg)*	*Percent of all seafood import volume*	*Time period of reporting*
NOAA Fisheries (2024). "Report on the Seafood Import Monitoring Program – FY 2023."	$6,432,182,862	30%	773,238,047.51	32%	FY 2023
NOAA (2022). "Developing a priority list of species for consideration under the Seafood Import Monitoring Program."	$10,383,488,198	47%	1,330,222,507.77	45%	FY 2020
NOAA (2021). "Efforts to prevent importation of seafood harvested through illegal, unreported, and unregulated fishing and address imported seafood fraud."	$10,354,659,786		1,330,222,508		FY 2020
NOAA (2016). "Final rule to implement U.S. Seafood Import Monitoring Program, RIN 0648-BF09. Final regulatory impact review and final regulatory flexibility analysis."	$9.34 billion	46%	990,275,614	39%	2014

Database Construction

We combine data from multiple sources. Figure S1 shows the databases we combine, the key fields we use from the databases, and the fields we use to link databases in bold.

Figure S1: Data pipeline.

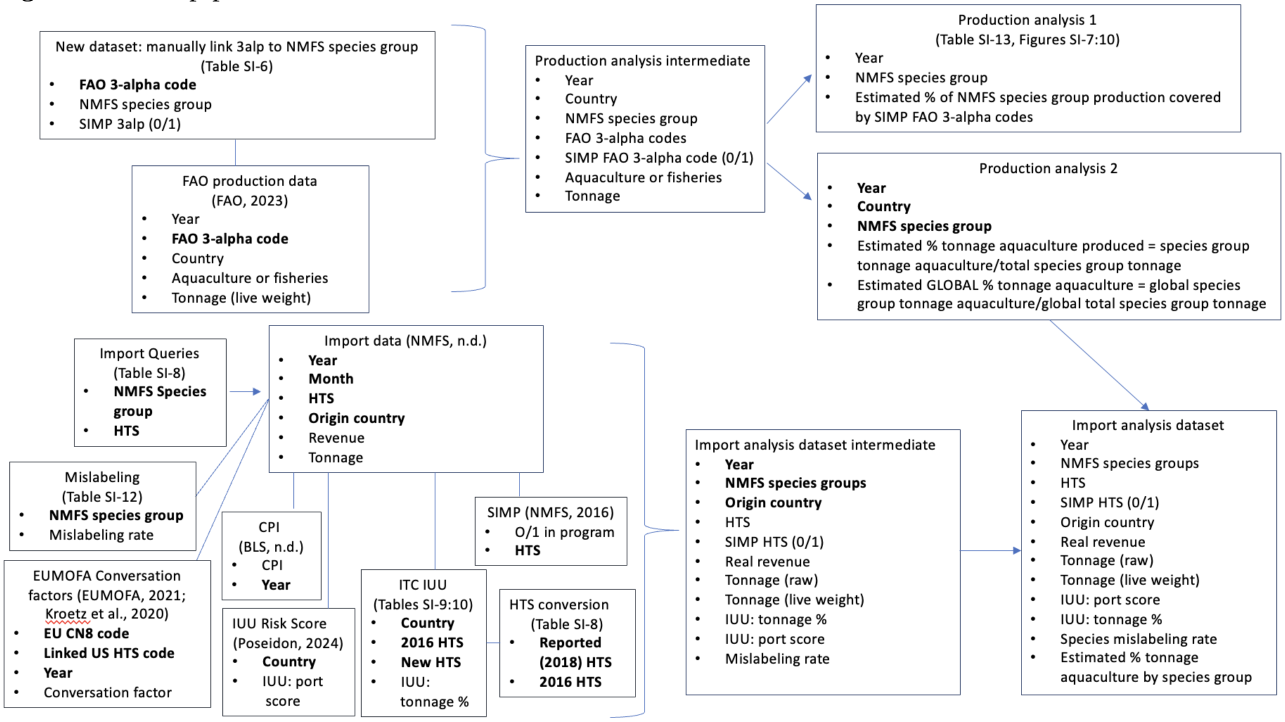

Trade Data

We identified program scope using seafood trade data available through the National Marine Fisheries Service's Commercial Fisheries Statistics (NMFS n.d.). These data originate with Customs and Border Protection and the Foreign Trade Division of the Census Bureau. We identified imports ("shipments") to the U.S. by country of origin, species group ("NMFS species group"), and 10-digit U.S. Harmonized Trade Schedule (HTS) code. It is possible to query the database by species group, which we refer to as the NMFS species group. This is done by running a query for each species group, extracting HTS codes included in each output file, and creating a linkage between HTS code reported in the trade data and NMFS species group. We include the mapping of reported 2016 HTS codes to NMFS species groups in Table S13.

We only analyze edible fish products and therefore follow the convention in the literature of dropping non-edible codes and byproducts. We do this by removing records associated with any non-edible HTS codes and byproducts through manual inspection of query name and/or product name where necessary. These products either do not have an assigned live weight conversion factor or it is set to zero (EUMOFA 2021). Removing these records from analysis follows recent work including Kroetz et al. (2020). We include the list of HTS codes and associated product names removed as non-edible codes and byproducts in Table S11.

We made several adjustments to the NMFS species groupings to match language of the final SIMP rule. There is no NMFS species group for blue crab, so we delineated this group using HTS product descriptions that include "Callinectes" to follow language from the SIMP rule. There are also two sets of trade records that do not match to individual species. Catch-all other groups, e.g., "Other tuna" and "Other crab", are less resolved than species groups that identify specific species such as "Snow Crab" and "Yellowfin Tuna", but we retain them following the construction of the NMFS species groups. There is also a set of HTS codes that include not specifically provided for (NSPF) products and cannot be clearly matched to any one species, e.g., "Fish NSPF," which we assign to their own group called "Unidentified Species." Also included in the "Unidentified Species" group is any HTS code with a product description that included more than one species. See Table S10 for the full list of HTS products included in each of the other and Unidentified Species groups.

We converted all trade tonnages to estimated weight at capture or harvest ("live weight") for all NMFS trade data products using mass conversion ratios from the European Market Observatory for Fisheries and Aquaculture Products (EUMOFA) (EUMOFA 2021). These ratios enable comparison of volumes for products across different forms (e.g., canned meat fillets, etc.). Additionally, this allows for a justifiable linkage to production data to estimate the percentage of production from fisheries versus aquaculture. We follow Kroetz et al. (2020) in linking EU Combined Nomenclature-8 codes to U.S. HTS codes, which we use to merge the conversion factor data to the 2016 trade data. We multiply reported tonnage (raw weight in kilograms) by the product-specific conversion factor to estimate live weight tonnage, then divide by 1,000 to convert to metric tonnes.

Percent Aquaculture Estimates

For each country the U.S. imported from, we estimated production tonnage by method of production (i.e., aquaculture or fishery), and the percentage produced via aquaculture for each species group. We accessed the production data through the FishStatJ Production Database maintained by the Fisheries and Aquaculture Department of the Food and Agriculture Organization (FAO) (FAO 2023). FishStatJ is a compilation of production data shared with FAO by national governments.

We manually linked NMFS species groups to FAO Aquatic Sciences and Fisheries Information System (ASFIS) species 3-alpha codes. The FAO data use scientific names and generally are a finer resolution than the NMFS species groups. This involves manually assigning the 3-alpha codes to NMFS species groups using a search of common and scientific names with ad-hoc additions following methods from Kroetz et al. (2020). The mapping from each ASFIS 3-alpha code to NMFS species group is shown in Table S12.

We developed estimates of the percentage of production likely attributable to aquaculture and to each ASFIS 3-alpha code for each country and species group code pair in the trade data. For each country, we assumed the quantity imported of a NMFS species group is proportional to the exporting country's production of each ASFIS 3-alpha code that falls under that NMFS species group. For example, we linked two ASFIS 3-alpha codes, "Atlantic bluefin tuna" and "Pacific bluefin tuna", to a NMFS species group, "bluefin tuna." This assumes the country of origin is equivalent to the country of production, which is not the case for exports that underwent foreign processing. However, over 75% of exports are produced by the exporting country (Gephart et al. *in press*) and most HTS codes reasonably separate species that are primarily wild caught versus farmed, so this is unlikely to affect the results. For each NMFS species group and country pair, we summed total production quantity from the country as well as aquaculture production (we considered any production source labeled "Aquaculture production (brackishwater)", "Aquaculture production (freshwater)", or "Aquaculture production (marine)" as aquaculture) and calculated the percentage of the total live weight production attributable to aquaculture. Following these steps, we developed a database by species group and country of the estimated percentage of production from aquaculture origin.

We then assigned each trade record the percentage aquaculture associated with the country of origin and species group. We matched approximately 85% of live weight tonnage with a species identified (i.e., not including other or unidentified). For the remaining unmatched records, we applied the global weighted average percent aquaculture for the species group and were able to match all remaining records with a species identified. Regarding the other and unidentified species groups, we were able to estimate a percent aquaculture for all but the following least-resolved species groups: unidentified species, other aquatic invertebrates, other crustaceans, other molluscs, and other shellfish.

SIMP Status

NMFS identified 13 priority species groups and designated HTS codes within those species groups as subject to the final rulemaking (NMFS 2016b). For each SIMP HTS code, importers must report the associated ASFIS 3-alpha code and only a sub-set of these 3-alpha codes are subject to the full recordkeeping requirements. We choose to use HTS codes listed under SIMP to define treatment due to the structure of program reporting requirements. In accordance with the compliance guide and final rule document, any shipment imported under a SIMP HTS code must report the correct ASFIS 3-alpha code (NMFS 2019; NMFS 2016b). If the reported 3-alpha code also falls under the set of SIMP-designated 3-alpha codes, then the full SIMP documentation is required (NOAA Fisheries 2019). Therefore, importers must report some SIMP data, including only identifying the ASFIS 3-alpha code, for every shipment imported under a SIMP HTS code. For this reason, we use HTS codes to designate SIMP treated and untreated import records. We use the list of HTS codes included in the final rule to define SIMP treatment for products imported to the U.S. in 2016 (NMFS 2016b). We plot observed coverage using SIMP HTS codes to define treatment in Figure S9, Figure S10, and Figure S11.

Illegal, Unregulated, and Unreported (IUU) Fishing Indicators

The Final Rule language identifies the first program goal as targeting products "identified as being at particular risk of illegal, unreported, and unregulated (IUU) fishing" (NMFS 2016b). In addition to the broad goals related to IUU and fraud, the working group that led the selection of SIMP prioritized species groups identified enforcement capability, existence of a catch documentation scheme, complexity of the supply chain, known species substitution, history of mislabeling (other than misidentification of species), and history of fisheries violations as principles to use when selecting species for prioritization (NMFS 2016a, NMFS 2016b). Enforcement capacity, existence of catch documentation schemes, and history of violations all relate to IUU and are often components of assessing IUU risk and we consider these criteria in our selection of IUU indicators.

We use IUU fishing indicators from two sources in our analysis. Ideally, we would have 2016 data on IUU risk to match the analysis that is conducted using 2016 trade data. We are unaware of refined data for 2016, and instead use two databases that are slightly more recent. Our main results are from the International Trade Commission (ITC) report (USITC 2021) and are at the HTS code and country resolution for 2018. Qualitative risk criteria are used in this report to aid in estimating IUU quantity imported, which relies on country and fishery specific history of violations and current regulations (e.g., catch documentation schemes), among other factors (USITC 2021). The country and HTS level of resolution captures potential variability in IUU risk by species and country. The year 2018 is also justifiable because it is difficult to reduce IUU and it is the year of implementation of SIMP, and therefore less likely to have induced changes in IUU.

The ITC report estimates quantity imported that is likely to be from IUU sources, including for HTS codes that should be covered by SIMP. There are a few possible explanations why this may be the case. First, the ITC report is conducted using data from the first year of SIMP mandatory compliance, so there may not have been significant changes to supply chains yet. Next, it is possible, under the current SIMP rule, for there to remain some IUU in harvest locations provided that only the shipments subject to the full SIMP recordkeeping (i.e., by HTS and 3-alpha codes) have a legal catch record. Finally, monitoring and enforcement of SIMP may be imperfect and the goals of the program are to *reduce* IUU imports rather than eliminate them entirely (NMFS 2016b). Future work is needed to examine how SIMP affected imports of IUU product and the extent to which monitoring and enforcement are complete.

We also check the robustness of our results using the more widely used IUU Risk Index data (Macfadyen and Hosch 2023; Poseidon 2024). This dataset is at the country resolution, which makes it less resolved and therefore less preferred for our analysis. Furthermore, given that this is 2019 data, there are more substantive concerns that SIMP could have changed the IUU outcomes across countries and therefore affected the relative ranking of IUU hotspots. Country-level "response" to IUU, such as catch documentation schemes, is a type of indicator included in the IUU fishing index (Poseidon 2024).

Lastly, IUU is a concept applied to capture fisheries production. The SIMP rule outlines the focus on risk as related to "the identification of fish species likely to be most at risk of IUU fishing or seafood fraud" (NMFS 2016a, NMFS 2016b). This leaves ambiguous whether the risk assessment would be focused on the presence of IUU within species group imports or the presence of IUU at the harvest location. The latter is consistent with indicators of IUU that have been developed at the country-level and are measures of the relative concerns over IUU related to the capture fishing within the country and/or by country flagged vessels (Poseidon 2024). Given this ambiguity, for our primary results we focus on an index value corresponding to the rate of IUU products within species group imports. However, we also explore the robustness of our primary results to an index corresponding to the rate of IUU within capture production.

International Trade Commission IUU measures

We took several steps to create a measure of IUU risk for each species group as well as several other measures to use as robustness checks. We used the scores, combined with a set of weights representing the best estimate of quantity at risk of IUU for each species group, to assess IUU risk for each species group. One challenge is that some species groups have both SIMP and non-SIMP HTS codes included in the species group. We treat species groups with both SIMP and non-SIMP HTS codes as individual species groups. The ITC IUU data is organized by HTS code and country, so there is no issue in splitting SIMP and non-SIMP using HTS codes within species groups. One species group, snails, is not included in the ITC report and therefore we drop the associated records from our trade database.

A challenge in linking the 2018 ITC data to our 2016 trade data is that there was a change in HTS codes beginning in January 2017 (USITC 2023). Therefore, we created a mapping between the 2018 HTS codes in the ITC data and the HTS codes used in the 2016 import data. For all but two HTS codes, we were able to match each 2018 HTS code back to the less-refined 2016 HTS code that it likely stemmed from using product names. For example, “Abalone frozen/dried/salted/brine” is a 2016 HTS code that was split into two 2018 HTS codes: “Abalone frozen” and “Abalone dried/salted/brine.” Both of these 2018 HTS codes were linked back to the 2016 HTS code “Abalone frozen/dried/salted/brine.” We provide the full mapping of 2018 HTS codes to 2016 HTS codes in Table S13. There are two shrimp HTS codes introduced in 2018 that come from more than one 2016 code, so we aggregate the respective 2016 codes.

Our primary results relate to a measure of the likely relative proportion of IUU product within a species groups’ imports. Here the assumption is that risk is measured as a high proportion of IUU product in the imported tonnage. We used the ITC data on IUU quantity and total quantity for each HTS code and country. We first divided estimated quantity from IUU and produced via capture fisheries by total quantity (i.e., estimated percentage of HTS and country quantity that is IUU). For observations with entirely aquaculture production and/or a quantity of zero from IUU, we record a true zero percent from IUU. We then merged this to the 2016 import data at the HTS code and country level, using global species group information as a secondary match for observations in the import data with no match in the ITC data. We then calculated a weighted average, where the weight is the imported tonnage from the trade data, for all HTS and country codes within a species group to come up with a single score for the NMFS species groups. The IUU scores are shown for the NMFS species groups in Table S3.

We also created a measure of relative IUU risk from capture fishing using data from the ITC report (USITC 2021). We first divide estimated quantity from IUU and produced with capture fisheries by total capture quantity (i.e., estimated percentage of capture quantity that is IUU) at the HTS code and country scale. For observations with entirely aquaculture production and/or a quantity of zero from IUU, we record a true zero percent from IUU. We then merged this with the 2016 import data at the HTS code and country level, using global species group (max/min) as a secondary match for observations in the import data with no match in the ITC data. Using the 2016 trade data, now augmented with an IUU score at the HTS and country level, we applied the species group and country percent aquaculture to the HTS code and country pairs in the 2016 import data to calculate an estimate of the quantity imported from capture fisheries for each HTS code and country in our trade data. We then use this estimate of fisheries tonnage for each HTS and country combination as weights and the IUU risk score from the ITC report to calculate a weighted average IUU relative risk from capture fishing by species group.

To calculate estimated import quantity from capture fisheries at the species group level, we used 2016 FAO production data (FAO 2023) to calculate the percentage of production from aquaculture for each species group and country combination. We then multiply quantity imported (in live weight) by (1-percent aquaculture production), with data organized by species group and country. Here, we assume that imports are in proportion to production. We then summed over countries to estimate the quantity imported from capture fisheries for each species group. The IUU scores and capture tonnages are shown for the NMFS species groups in Table S4.

Table S3: Mean IUU score for each species group. The mean IUU score and overall species group IUU score is calculated using weights equal to the species group import tonnage.

NMFS Species Group	*Live Weight (Tonnes)*	*Mean IUU Score*	*SIMP (1=yes)*
Abalone - Non-SIMP	2,194	5.24	0
Abalone - SIMP	202	7.78	1
Anchovy	6,888	16.70	0
Atka Mackerel	111	15.24	0
Bass	433	0.19	0
Bonito	23,712	18.14	0
Butterfish	1,999	19.40	0
Capelin	5,458	8.42	0
Carp	90	0.00	0
Catfish	303,307	0.00	0
Clam	32,781	1.53	0
Cobia	916	1.62	0
Cockles	564	7.00	0
Conch	824	16.05	0
Crab: Blue	8,309	34.04	0
Crab: Dungeness	99	1.14	0
Crab: King	41,661	16.14	0
Crab: Snow	202,650	5.21	0
Crab: Swimming	37,626	25.35	0
Crawfish	15,558	5.55	0
Cuttlefish	3,600	34.79	0
Dolphin	48,409	21.77	1
Eels	7,963	0.01	0
Flatfish: Flounder	28,197	8.56	0
Flatfish: Halibut	11,798	5.32	0
Flatfish: Plaice	781	1.73	0
Flatfish: Sole	25,713	55.46	0
Flatfish: Turbot	3,020	3.83	0
Groundfish: Blue Whiting	165	24.92	0
Groundfish: Cod - Non-SIMP	0		0
Groundfish: Cod - SIMP	200,152	7.45	1
Groundfish: Cusk	4	1.26	0
Groundfish: Haddock	67,369	3.02	0
Groundfish: Hake	9,174	21.93	0
Groundfish: Ocean Perch	13,220	3.46	0
Groundfish: Pollock	103,704	12.96	0
Groundfish: Whiting	10,811	33.75	0
Grouper	7,204	49.96	1
Herring	24,820	6.78	0
Horse Mackerel Jack	1,768	29.22	0
Jellyfish	1,361	20.79	0
Krill	711	2.87	0
Lingcod	303	2.88	0
Lobster	78,705	5.79	0
Mackerel	36,738	14.64	0

Monkfish	390	46.80	0
Mullet	172	17.36	0
Mussels	97,194	0.09	0
Nile Perch	785	0.00	0
Octopus	26,890	38.00	0
Orange Roughy	4,075	10.45	0
Other Aquatic Invertebrates	5,250	1.07	0
Other Crab - Non-SIMP	11,665	22.33	0
Other Crab - SIMP	32,012	22.48	1
Other Crustaceans	19,146	4.47	0
Other Flatfish	5,179	9.90	0
Other Groundfish	72,665	15.84	0
Other Molluscs	13,976	12.20	0
Other Salmon	91,197	7.25	0
Other Shellfish	2,079	13.36	0
Other Tuna - Non-SIMP	799	38.03	0
Other Tuna - SIMP	415,835	11.61	1
Oysters	13,926	1.29	0
Perch Nspf	2,052	1.05	0
Pickerel	4,523	0.00	0
Pike	274	0.87	0
Rays Skates	53	13.10	0
Sablefish	2,271	2.82	0
Salmon: Atlantic	408,255	0.00	0
Salmon: Chinook	4,513	3.69	0
Salmon: Chum	4,235	4.10	0
Salmon: Coho	1,405	2.65	0
Salmon: Pink	8,423	11.83	0
Salmon: Sockeye	3,770	15.50	0
Sardine	73,830	21.12	0
Sauger	66	0.21	0
Scallops	132,745	0.72	0
Sea Bass	7,331	1.51	0
Sea Cucumber - Non-SIMP	277	26.92	0
Sea Cucumber - SIMP	2,681	5.74	1
Sea Urchin	2,437	1.79	0
Seabream	697	1.29	0
Shark	834	45.48	1
Shrimp - Non-SIMP	215,645	1.66	0
Shrimp - SIMP	653,872	2.43	1
Smelts	3,213	0.77	0
Snapper	24,897	35.63	1
Squid	91,466	27.27	0
Swordfish - Non-SIMP	3,486	15.93	0
Swordfish - SIMP	10,207	11.95	1
Tilapia	479,219	0.00	0
Toothfish	18,035	9.18	0
Trout	21,033	0.00	0
Tuna: Albacore	62,074	12.87	1
Tuna: Bigeye	4,678	14.93	1

Tuna: Bluefin	1,611	4.30	1
Tuna: Skipjack	283	17.91	1
Tuna: Yellowfin	22,581	11.64	1
Unidentified Species - Non-SIMP	246,958	16.05	0
Unidentified Species - SIMP	836	14.88	1
Yellow Perch	2,359	0.39	0

Table S4: Mean IUU score for each species group. The mean IUU score and overall species group IUU score is calculated using weights equal to the species group import tonnage from fisheries.

NMFS Species Group	Live Weight from Fisheries (Tonnes)	Mean IUU Score	SIMP (1=yes)
Abalone - Non-SIMP	700	8.44	0
Abalone - SIMP	98	15.28	1
Anchovy	6,888	16.70	0
Atka Mackerel	111	15.24	0
Bass	404	0.22	0
Bonito	23,712	18.87	0
Butterfish	1,999	19.40	0
Capelin	5,458	8.42	0
Carp	5	0.03	0
Catfish	14	0.25	0
Clam	10,765	6.82	0
Cobia	8	62.78	0
Cockles	564	9.81	0
Conch	824	16.05	0
Crab: Blue	8,309	34.04	0
Crab: Dungeness	99	1.14	0
Crab: King	41,661	16.14	0
Crab: Snow	202,650	5.21	0
Crab: Swimming	31,771	35.47	0
Crawfish	651	6.96	0
Cuttlefish	3,600	34.79	0
Dolphin	48,409	21.77	1
Eels	119	1.66	0
Flatfish: Flounder	7,451	12.20	0
Flatfish: Halibut	10,847	4.93	0
Flatfish: Plaice	781	1.73	0
Flatfish: Sole	25,487	55.87	0
Flatfish: Turbot	890	3.44	0
Groundfish: Blue Whiting	165	24.92	0
Groundfish: Cod - Non-SIMP	0		0
Groundfish: Cod - SIMP	200,098	7.45	1
Groundfish: Cusk	4	1.26	0
Groundfish: Haddock	67,369	3.02	0
Groundfish: Hake	9,174	21.93	0
Groundfish: Ocean Perch	12,684	3.49	0
Groundfish: Pollock	103,704	12.96	0
Groundfish: Whiting	10,811	33.75	0
Grouper	6,780	52.14	1
Herring	24,820	6.78	0
Horse Mackerel Jack	1,767	29.24	0
Jellyfish	1,361	22.44	0
Krill	711	3.73	0
Lingcod	303	2.88	0

Lobster	78,651	5.77	0
Mackerel	36,735	14.64	0
Monkfish	390	46.80	0
Mullet	121	26.89	0
Mussels	44,955	0.10	0
Nile Perch	769	0.00	0
Octopus	26,889	38.00	0
Orange Roughy	4,075	10.45	0
Other Aquatic Invertebrates	0		0
Other Crab - Non-SIMP	9,796	22.42	0
Other Crab - SIMP	22,961	25.00	1
Other Crustaceans	0		0
Other Flatfish	5,179	10.23	0
Other Groundfish	72,665	15.84	0
Other Molluscs	0		0
Other Salmon	11,552	4.98	0
Other Shellfish	0		0
Other Tuna - Non-SIMP	799	38.03	0
Other Tuna - SIMP	415,835	11.61	1
Oysters	1,706	17.00	0
Perch Nspf	1,693	1.26	0
Pickerel	4,523	0.00	0
Pike	248	0.68	0
Rays Skates	53	13.10	0
Sablefish	2,271	2.82	0
Salmon: Atlantic	7,959	4.47	0
Salmon: Chinook	4,326	3.85	0
Salmon: Chum	4,235	4.10	0
Salmon: Coho	1,262	2.97	0
Salmon: Pink	8,423	11.83	0
Salmon: Sockeye	3,770	15.50	0
Sardine	73,830	21.12	0
Sauger	65	0.22	0
Scallops	49,263	2.21	0
Sea Bass	496	21.93	0
Sea Cucumber - Non-SIMP	176	21.18	0
Sea Cucumber - SIMP	2,089	18.34	1
Sea Urchin	2,433	1.77	0
Seabream	117	11.38	0
Shark	834	45.48	1
Shrimp - Non-SIMP	55,734	15.88	0
Shrimp - SIMP	203,261	15.99	1
Smelts	3,154	0.80	0
Snapper	24,189	36.82	1
Squid	91,466	27.27	0
Swordfish - Non-SIMP	3,486	15.93	0
Swordfish - SIMP	10,207	11.95	1
Tilapia	7,518	0.00	0
Toothfish	18,035	9.18	0
Trout	61	0.06	0

Tuna: Albacore	62,074	12.87	1
Tuna: Bigeye	4,678	14.93	1
Tuna: Bluefin	569	9.80	1
Tuna: Skipjack	283	17.91	1
Tuna: Yellowfin	22,581	11.64	1
Unidentified Species - Non-SIMP	0		0
Unidentified Species - SIMP	0		1
Yellow Perch	2,341	0.39	0

IUU Fishing Risk Index

As a metric of IUU risk, we also used the IUU Fishing Index, which quantifies countries' multidimensional exposure and response to IUU fishing (Macfadyen and Hosch 2023). The description of the database notes that "few indicators are likely to be especially robust in a conceptual sense as a measure of IUU fishing risk in a country when used on their own or in isolation" (Poseidon Aquatic Resource Management Ltd. 2024). Therefore, we use the aggregate score in our analysis. The indicator scores range from 1-5 (1 = best performing, 5 = poorly performing) (Poseidon Aquatic Resource Management Ltd. 2024). While coverage of the IUU Fishing Index for exporter countries is incomplete, the IUU Fishing Risk Index includes information for 152 countries. For the countries the USA imported from in 2016, the countries in the database cover 99% of import records. The majority of the records missing the IUU Fishing Risk score are associated with Faroe Islands. The vast majority of these records are farmed salmon and, given that IUU is generally a capture-production concept, dropping these records will not exclude a high-IUU area. In our analysis with the IUU Fishing Risk Index data we used the estimated capture quantity (i.e., removing the estimated percent of production from aquaculture based on our FAO data calculations) associated with each species group as a measure of the quantity imported at risk of IUU.

Fraud Indicator: Seafood Mislabeling Rates Meta-Analysis

With our second indicator, we aimed to capture a measure of the second goal of the SIMP associated with targeting importation of certain fish and fish products identified as being at particular risk seafood fraud (NMFS 2016b). In prior work, seafood fraud has been characterized as intentional mislabeling of seafood products and falls under a broader umbrella of seafood mislabeling that can be intentional or unintentional (e.g. Kroetz et al. (2020)). Because of the difficulty in determining intent of mislabeling, we used data on seafood mislabeling as the best available data related to seafood fraud rates. Furthermore, a broader consideration of mislabeling is also consistent with health concerns (e.g., Love et al. 2021) and the principles of known species substitution, history of mislabeling (other than misidentification of species), and potentially complexity of the supply chain (NMFS 2016a, NMFS 2016b).

Models

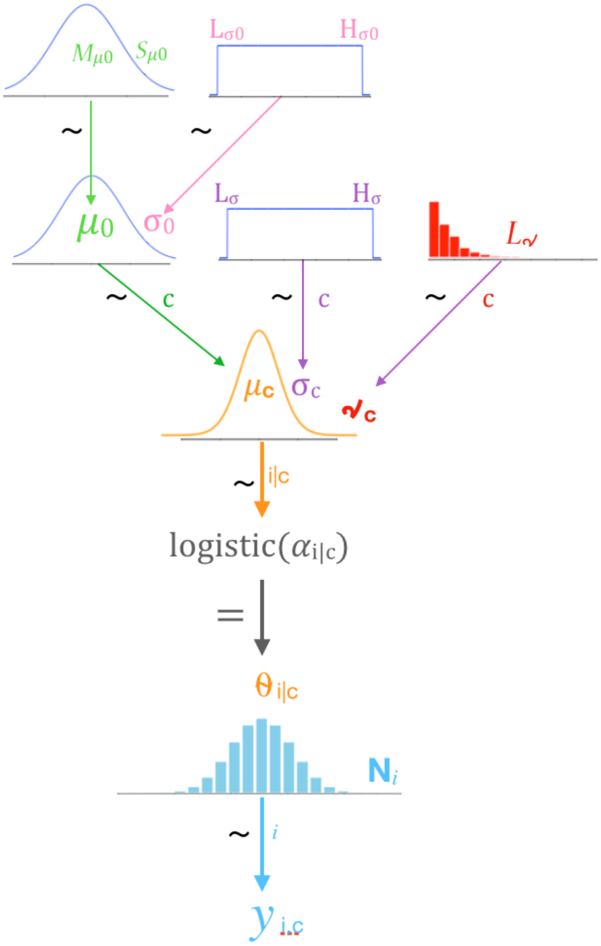

Figure S2. Schematic of a robust logistic regression model with two levels of hierarchy. Parameter definitions are described, as well as the relationships between the parameters and the data The Greek characters in the diagram are parameters or hyper-parameters estimated by the model, while the Latin characters are values that describe the prior distributions that are provided to the model. Schematic adopted from Kruschke 2015.

Our statistical estimation uses the data from and follows the approach and models used for a global meta-analysis on seafood mislabeling (Luque and Donlan 2019). We use a Bayesian two-level hierarchical model to estimate mislabeling rates in the United States. Each mislabeling species group is a different category *c* in the model, described in Figure S2. We estimate the mean (μ_c) and variance (σ_c) for each seafood product. The likelihood function is described as,

$$y_{i,c} \mid \theta_{i,c} \sim \text{Bin}(\theta_{i,c}\ ,\ N_{i,c})$$

where, $y_{i,c}$ represents the number of mislabeled samples by study *i* out of N_i (total # samples in each study *i*) within each category of seafood product *c*. $Y_{i,c}$ is assumed to be a random variable from a binomial distribution that is defined by the mean $\theta_{i,c}$ and the number of samples ($N_{i,c}$). There are as many $\theta_{i,c}$ as number of studies within each category of mislabeling species group (which is a proportion and thus can only take values between 0 and 1).

We transform the parameters $\theta_{i|c}$ using a logistic transformation and use a *t* distribution as a conjugate prior of the likelihood function. The transformation takes the form,

$$\alpha_{i|c} = \text{logit}(\theta_{i|c}).$$

The estimated $\alpha_{i|c}$ are *t* distributed random values around the central tendency μ_c. A *t* distribution is similar to a normal distribution with two parameters that control its mean μ and its width σ. The *t* distribution has a third parameter that controls the heaviness of its tails, the normality parameter ν, and describes any deviation from normality when there are outliers (i.e., data values that fall unusually far from a model's expected value). Thus, the *t* distribution is robust against outliers (i.e., robust logistic regression), and the parameters $\alpha_{i|c}$ can be described as,

$$\alpha_{i,c} \mid \mu_c, \sigma_c, \nu_c \sim t(\ \mu_c\ ,\ \sigma_c\ ,\ \nu_c\ )$$

where the probability of $\alpha_{i,c}$ given the three hyper-parameters follows a *t* distribution described by the μ_c, σ_c, and ν_c (Figure S2). There are five types of hyper-parameters in the model, which are described below, along with the values of their priors. The values that describe the priors can be whatever is an appropriate

description of real prior knowledge (i.e., informative prior) or vague and wide enough in the scale of the data (i.e., non-informative prior) so that the model can explore a wide range of possible values to estimate the parameters given by the model and data (Luque and Donlan 2019). Some argue that a mildly-informative prior can improve model estimates especially with small sample sizes (Gelman et al. 2008). In our model, we use both midly-informative and non-informative priors, which are described below.

- μ_c ~ Norm(μ_0,σ_0). There is an estimated mislabeling rate for each category of a factor (μ_c), which are the means of the distributions that describe the spread of mislabeling rates of all studies within each category (all $\alpha_{i,c}$). Each rate is described by a normal distribution defined by μ_0 and σ_c.
  - μ_0 ~ Norm($M_{\mu0}$,$S_{\mu0}$). The mean μ_0 describes the mean mislabeling rate of all seafood products means (μ_c), and is given a normal prior described by the two values, $M_{\mu0}$ and $S_{\mu0}$.
  - σ_0 ~ Unif(L_σ,H_σ). The scale σ_0 describes the precision of the global mean estimation μ_0, which is given a uniform prior described by a minimum and maximum value (L_σ and H_σ, respectively).

In our case, we have prior knowledge of seafood mislabeling from a recent global meta-analysis, including the estimates for 28 mislabeling species groups (Luque and Donlan 2019). Thus for μ_0 , we use the mean (9%) and standard deviation (65%) for these groups as priors, transforming the values on a logistic scale ($M\mu0$= -2.40) and doubling the standard deviation ($S\mu0$= 1.26). For σ_0, we use a uniform distribution that results in a non-informative prior (i.g., vague and noncommittal), allowing for broad range of values for hyper-parameter. All prior uniform distributions are described by two values: a lower (L_σ = 0) and a higher value (H_σ = 100).

- σ_c ~ Unif(L_σ,H_σ). The hyper-parameter σ_c describes the precision of the mean (i.e., scale) of each category of a factor, which in our case is mislabeling species groups. The model estimates a σ_c for each mislabeling species groups according to spread of the mislabeling rates within each group. This is equivalent to allowing the model to account for any heterogeneity in variances, which is an advantage compared to models that assume homogeneity of variances, a common constraint with frequentist analyses. For σ_c, we also use a uniform distribution that results in a non-informative prior, allowing for broad range of values for hyper-parameter ((L_σ = 0; H_σ = 100).

- ν_c ~ Exp(1/30). The normality hyper-parameter ν_c describes the heaviness of the tails of the distribution for each category of seafood product. It is estimated and given a broad exponential prior. Typically, one is not interested in the exact estimated value of ν_c; rather, in giving the model flexibility to use heavy tails if the data demand them. The normality hyper-parameter ν_c is estimated and given a broad exponential prior that can range from 1 to ∞. When ν_c =1 the *t* distribution has very long tails, while when ν_c approaches ∞ the *t* distribution becomes normal. Values of ν_c > ~30 indicate a normal distribution and values, while values < ~30 indicate a distribution with heavier tails than a normal distribution.

Models Performance and Diagnostics

From the Bayesian models, we generate Markov Chain Monte Carlo (MCMC) samples from posterior distributions. We run each model with three parallel chains for 1,000,000 iterations without thinning. The values from the MCMC sample of the posterior distribution must be representative from the true posterior distribution and the parameter estimates must be accurate and stable (i.e., the mean and HDI should not differ significantly when the model is re-run). We carried out a series diagnostic tests in order to check whether the MCMC sample from the posterior distribution of each model is representative of the true posterior distribution and whether the estimates accurate and stable.

Representativeness. First, we assessed whether each MCMS sample was a representative sample from the posterior distribution via visual examination. *Trace plots* reveal if all chains meander and overlap with each other, indicating they are representative of the posterior distribution (Figure S3). They also allow for the assessment of convergence and the necessary so-called *burn*-in period (i.e., the number of steps that should be excluded from the MCMC sample). We used a *burn-in* rate of 1,000 for all models. *Density plots* are smoothed histograms of the parameter values sampled by each chain, which provide another visual tool to assess overlap (Figure S3). Second, we assess representativeness numerically with the Gelman-Rubin statistic, which is known as the *shrink factor*. We used the heuristic of a Gelman-Rubin statistic >1.1 as a warning that the chains have potentially not converged adequately (Kruske 2015). We used three chains for all models.

Accuracy. First, we also assessed visually if MCMC samples were large enough for a stable and accurate numerical estimates of the prior distribution. *Autocorrelation plots* show the degree of autocorrelation between chains (Figure S3). Second, we assess accuracy numerically with the Effective Sample Size (ESS), which divides the actual sample size by the amount of autocorrelation. We used a heuristic of a ESS < 10,000 as a warning that the sample size may not be sufficient with respect to accuracy (Kruske 2015). Last, we also use the Monte Carlo Standard Error (MCSE) as a numerical diagnostic of accuracy. The MCSE indicates the estimated standard deviation of the sample mean in the chain, on the scale of the parameter value. In particular, we use the MCSE to assess the accuracy of the posterior mean, using a heuristic of >5% (% of the standard deviation) as a warning that sample size may not be sufficient with respect to accuracy (Kruske 2015; Breheny n.d.). Numerical diagnostics for all models are reported in the supplementary tables. All analysis were conducted in the statistical language R and JAGs (Plummer 2017; R Development Core Team 2017).

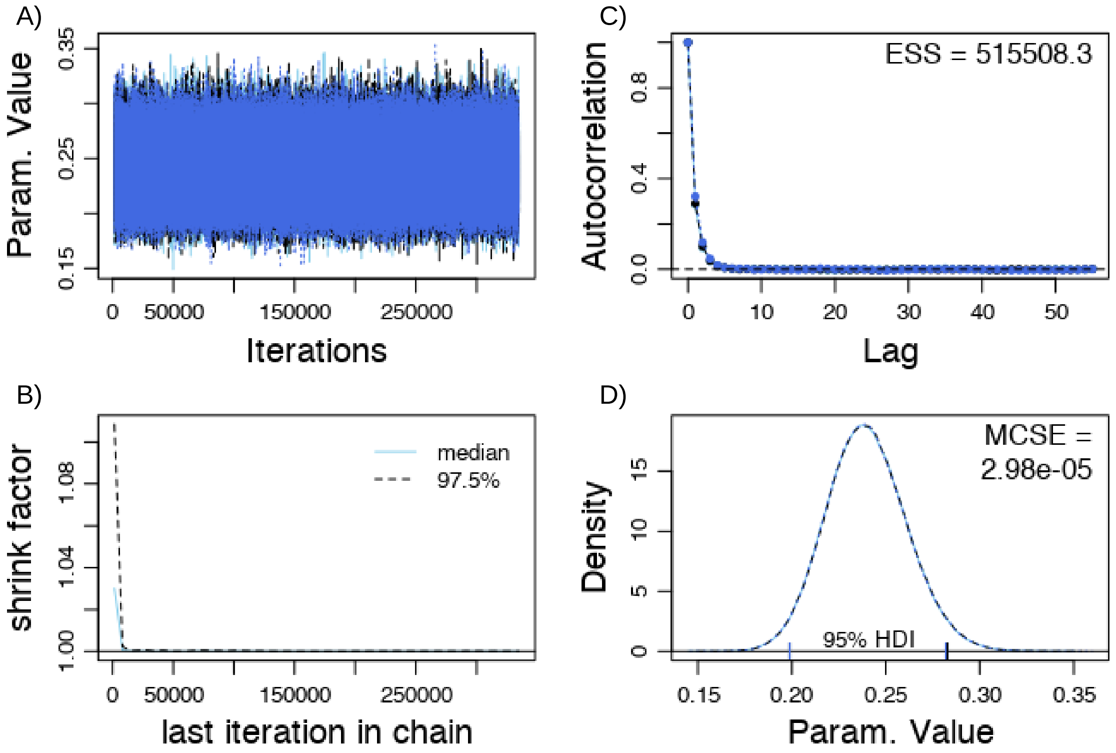

Figure S3. Visual diagnostics to assess the representativeness and accuracy of MCMC samples. A) Trace plots reveal if all chains meander and overlap with each other, indicating they are representative of the posterior distribution. B) The shrink factor informs if the chains have converged adequately. C) Autocorrelation plots show the degree of autocorrelation between chains, which informs Effective Sample Size. D) Density plot is a smoothed histogram of a parameter value sampled by each chain. We used the function *diagMCMC* from Kruschke 2015 to generate the visual diagnostics.

Model Results with Informative Priors from Global Analysis.

Figure S4. Posterior probability density of US mislabeling rates for 16 mislabeling species groups, which have ≥5 studies. Posterior mode (vertical line) and 95% HDI (horizontal line) are shown, along with the naive mean (▲). Products with an * represent more than one species. Posterior mode, 95% HDI, and sample size (N, number of studies) are shown on the right side (A 0 indicates a value <1%). Study-level mislabeling data are also shown with dot size proportional to number of samples.

.

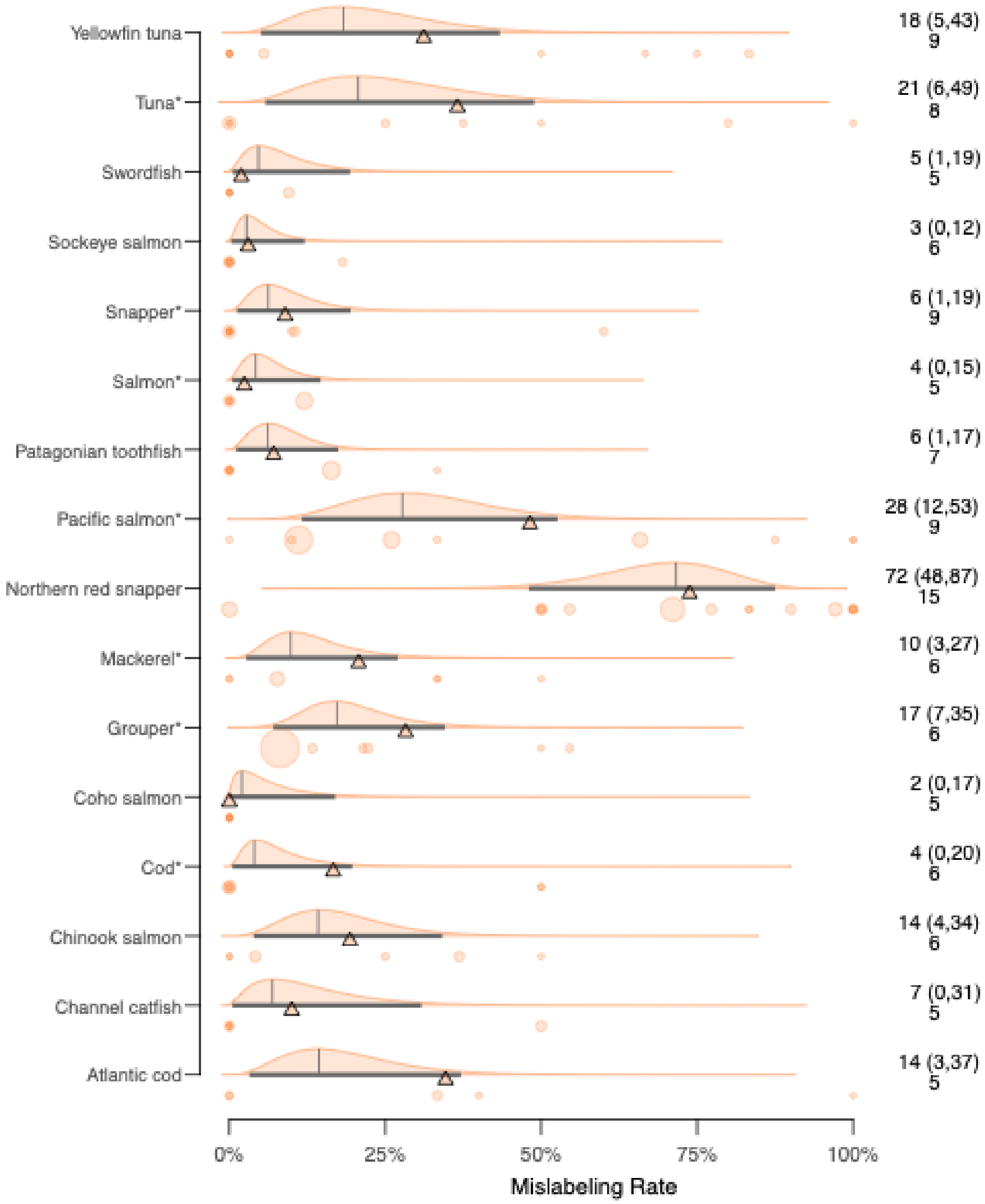

Table S5. US mislabeling estimates for 16 mislabeling species groups, which have ≥5 studies. The number of studies (n), naive mean, posterior mode, mean, and median, along with the 95% HDI are reported. Common names correspond to Fishbase. Products with an * represent more than one species.

**Mislabeling Species Group**	**N**	**Naive Mean**	**Mean**	**Median**	**Mode**	**Lower 95% HDI**	**Higher 95% HDI**
Atlantic Cod (*Gadus morhua*)	5	35%	19%	17%	14%	3%	37%
Channel catfish (*Ictalurus punctatus*)	5	10%	13%	11%	7%	0%	31%
Chinook salmon (*Oncorhynchus tshawytscha*)	6	19%	18%	17%	14%	4%	34%
Cod*	6	17%	8%	7%	4%	0%	20%
Coho salmon (*Oncorhynchus kisutch*)	5	0%	6%	5%	2%	0%	17%
Grouper*	6	28%	20%	19%	17%	7%	35%
Mackerel*	6	21%	13%	12%	10%	3%	27%
Northern red snapper (*Lutjanus* [illegible]	1[illegible]	74%	69%	70%	72%	48%	87%
Pacific salmon*	9	48%	31%	30%	28%	12%	53%
Patagonian toothfish (*Dissostichus eleginoides*)	7	7%	9%	8%	6%	1%	17%
Salmon*	5	2%	7%	6%	4%	0%	15%
Snapper*	9	9%	9%	8%	6%	1%	19%
Sockeye salmon (*Oncorhynchus nerka*)	6	3%	5%	4%	3%	0%	12%
Swordfish (*Xiphias gladius)*	5	2%	8%	7%	5%	1%	19%
Tuna*	8	37%	26%	24%	21%	6%	49%
Yellowfin tuna (*Thunnus albacares*)	9	31%	23%	21%	18%	5%	43%

Table S6. Diagnostics for hierarchical US seafood model. Effective sample size (ESS), Markov chain standard error (MCSE) and the Gelman-Rubin shrink reduction factor (Shrink Factor) for the mode (μ) and variance (σ) estimates for each model. The model performed well for all products: ESS is >10,000; the Shrink Factor <1.1; MCSE as a % of the standard deviation is <5%.

Mislabeling Species Group	μ			σ		
	ESS	MCSE	Shrink Factor	ESS	MCSE	Shrink Facto
Atlantic Cod (*Gadus morhua*)	826,569	0.0004	1.000	837,470	0.0015	1.000
Channel catfish (Ictalurus punctatus)	789,743	0.0004	1.000	907,710	0.0012	1.000
Chinook salmon (Oncorhynchus tshawytscha)	916,697	0.0003	1.000	824,103	0.0015	1.000
Cod*	66,235	0.0004	1.001	472,361	0.0015	1.000
Coho salmon (Oncorhynchus kisutch)	397,046	0.0002	1.000	734,281	0.0016	1.000
Grouper*	979,790	0.0003	1.000	949,145	0.0014	1.000
Mackerel*	805,518	0.0003	1.000	725,077	0.0016	1.000
Northern red snapper (Lutjanus campechanus)	90,237	0.0006	1.000	63,857	0.0023	1.000
Pacific salmon*	662,024	0.0004	1.000	819,248	0.0010	1.000
Patagonian toothfish (Dissostichus eleginoides)	540,668	0.0002	1.000	627,982	0.0016	1.000
Salmon*	422,593	0.0002	1.000	767,123	0.0016	1.000
Snapper*	800,999	0.0002	1.000	811,803	0.0012	1.000
Sockeye salmon (Oncorhynchus nerka)	360,773	0.0002	1.000	715,518	0.0016	1.000
Swordfish (Xiphias gladius)	577,528	0.0002	1.000	738,422	0.0016	1.000
Tuna*	667,239	0.0005	1.000	55,471	0.0017	1.004
Yellowfin tuna (Thunnus albacares)	849,652	0.0004	1.000	832,794	0.0010	1.000

Linking Mislabeling Species Group Information to the Main Database.

We used data on mislabeling derived from a Bayesian meta-analysis following the approach in Luque and Donlan (2019) to estimate rates of seafood mislabeling, as described above. Generally, the mislabeling species groups we were able to estimate mislabeling rates for aligned with the NMFS species groups (Table S7). The NMFS species groups and associated mislabeling rates – including for NMFS species groups where sufficient data were not available to estimate a rate – are listed in Table S8.

Table S7: Mislabeling species groups to NMFS species group link.

NMFS Species Group	*Mislabeling Species Group*
Atka Mackerel	Mackerel*
Catfish	Channel catfish (Ictalurus punctatus)
Groundfish: Cod	Cod*
Grouper	Grouper*
Mackerel	Mackerel*
Other Tuna	Tuna*
Salmon: Atlantic	Salmon*
Salmon: Chinook	Chinook salmon (Oncorhynchus tshawytscha)
Salmon: Chum	Pacific salmon*
Salmon: Coho	Coho salmon (Oncorhynchus kisutch)
Salmon: Pink	Pacific salmon*
Salmon: Sockeye	Sockeye salmon (Oncorhynchus nerka)
Snapper	Snapper*
Swordfish	Swordfish (Xiphias gladius)
Toothfish	Patagonian toothfish (Dissostichus eleginoides)
Tuna: Albacore	Tuna*
Tuna: Bigeye	Tuna*
Tuna: Bluefin	Tuna*
Tuna: Skipjack	Tuna*
Tuna: Yellowfin	Yellowfin tuna (Thunnus albacares)

Table S8: Mode mislabeling rates for each NMFS species group.

NMFS Species Group	*Species Group Value ($2016)*	*Live Weight (Tonnes)*	*Mode Mislabeling Rate*	*SIMP (1=yes)*
Abalone - Non-SIMP	18,831,270	2,194		0
Abalone - SIMP	18,831,270	202		1
Anchovy	35,289,864	6,888		0
Atka Mackerel	320,167	111	0.13	0
Bass	1,189,115	433		0
Bonito	29,491,723	23,712		0
Butterfish	6,270,384	1,999		0
Capelin	5,062,976	5,458		0
Carp	319,205	90		0
Catfish	405,640,412	303,307	0.13	0
Clam	73,060,788	32,781		0
Cobia	7,655,399	916		0
Cockles	1,971,091	564		0
Conch	7,651,855	824		0
Crab: Blue	74,487,600	8,309		0
Crab: Dungeness	1,188,342	99		0
Crab: King	280,452,715	41,661		0
Crab: Snow	658,027,010	202,650		0
Crab: Swimming	355,100,891	37,626		0
Crawfish	74,479,386	15,558		0
Cuttlefish	16,435,053	3,600		0
Dolphin	199,589,175	48,409		1
Eels	57,435,463	7,963		0
Flatfish: Flounder	51,818,147	28,197		0
Flatfish: Halibut	127,443,823	11,798		0
Flatfish: Plaice	2,617,428	781		0
Flatfish: Sole	47,844,110	25,713		0
Flatfish: Turbot	10,942,604	3,020		0
Groundfish: Blue Whiting	218,654	165		0
Groundfish: Cod - Non-SIMP	464,736,518	0		0
Groundfish: Cod - SIMP	464,736,518	200,152	0.08	1
Groundfish: Cusk	15,171	4		0
Groundfish: Haddock	139,012,757	67,369		0
Groundfish: Hake	14,322,105	9,174		0
Groundfish: Ocean Perch	15,562,680	13,220		0
Groundfish: Pollock	94,649,995	103,704		0
Groundfish: Whiting	13,017,189	10,811		0
Grouper	48,751,184	7,204	0.20	1
Herring	49,238,383	24,820		0
Horse Mackerel Jack	10,419,822	1,768		0
Jellyfish	5,965,607	1,361		0
Krill	1,635,162	711		0
Lingcod	1,540,532	303		0
Lobster	1,347,764,128	78,705		0
Mackerel	56,801,874	36,738	0.13	0
Monkfish	428,712	390		0

Mullet	402,091	172		0
Mussels	119,937,580	97,194		0
Nile Perch	2,562,639	785		0
Octopus	123,312,878	26,890		0
Orange Roughy	18,170,615	4,075		0
Other Aquatic Invertebrates	20,369,178	5,250		0
Other Crab - Non-SIMP	220,977,846	11,665		0
Other Crab - SIMP	220,977,846	32,012		1
Other Crustaceans	56,138,019	19,146		0
Other Flatfish	21,969,029	5,179		0
Other Groundfish	126,946,982	72,665		0
Other Molluscs	33,396,513	13,976		0
Other Salmon	422,532,695	91,197		0
Other Shellfish	3,720,788	2,079		0
Other Tuna - Non-SIMP	1,038,471,370	799	0.26	0
Other Tuna - SIMP	1,038,471,370	415,835	0.26	1
Oysters	74,463,990	13,926		0
Perch Nspf	8,193,180	2,052		0
Pickerel	19,333,185	4,523		0
Pike	1,007,470	274		0
Rays Skates	84,128	53		0
Sablefish	4,532,914	2,271		0
Salmon: Atlantic	2,686,933,186	408,255	0.07	0
Salmon: Chinook	30,851,298	4,513	0.18	0
Salmon: Chum	10,709,756	4,235	0.31	0
Salmon: Coho	5,712,436	1,405	0.06	0
Salmon: Pink	29,653,200	8,423	0.31	0
Salmon: Sockeye	22,964,941	3,770	0.05	0
Sardine	124,400,385	73,830		0
Sauger	312,930	66		0
Scallops	328,484,391	132,745		0
Sea Bass	39,542,781	7,331		0
Sea Cucumber - Non-SIMP	27,917,939	277		0
Sea Cucumber - SIMP	27,917,939	2,681		1
Sea Urchin	7,821,308	2,437		0
Seabream	4,628,700	697		0
Shark	1,557,792	834		1
Shrimp - Non-SIMP	5,704,894,484	215,645		0
Shrimp - SIMP	5,704,894,484	653,872		1
Smelts	7,999,092	3,213		0
Snail	5,596,859	1,280		0
Snapper	128,132,938	24,897	0.09	1
Squid	306,658,279	91,466		0
Swordfish - Non-SIMP	87,356,260	3,486	0.08	0
Swordfish - SIMP	87,356,260	10,207	0.08	1
Tilapia	754,805,482	479,219		0
Toothfish	243,512,560	18,035	0.09	0
Trout	120,977,955	21,033		0
Tuna: Albacore	139,077,984	62,074	0.26	1
Tuna: Bigeye	37,005,902	4,678	0.26	1

Tuna: Bluefin	27,476,892	1,611	0.26	1
Tuna: Skipjack	375,582	283	0.26	1
Tuna: Yellowfin	175,862,674	22,581	0.23	1
Unidentified Species - Non-SIMP	803,677,145	246,958		0
Unidentified Species - SIMP	803,677,145	836		1
Yellow Perch	21,416,368	2,359		0

Hypothesis Testing: Extended Results

Although we observed a higher mean IUU index score and mislabeling rate for our observed population, we proceeded with more formal hypothesis testing. We used the hypothesis testing to explore the performance of SIMP as implemented relative to a relevant alternative treatment approach: random assignment of SIMP status within the finite population of species groups. Specifically, we tested the null hypothesis that the observed difference in the observed mean SIMP and non-SIMP scores for the outcome (IUU or fraud) equals that which we would expect under random assignment of SIMP status to species groups. We tested the null hypothesis against the alternative that the observed difference in the mean outcomes score between SIMP and non-SIMP products is greater than that under random assignment of species groups to SIMP.

We used permutation tests to conduct our hypothesis testing. We used the permute command in STATA and repeatedly randomly assigned SIMP designation to species groups. Throughout the repetitions we retained the same list of species groups and held the number of SIMP-designated species groups constant. We randomly assigned the SIMP designations to species groups and calculated the weighted mean for the SIMP species group and the weighted mean for non-SIMP species group outcome (IUU score or mislabeling rate) for each repetition. In the case of the mislabeling rates where rates are not known for many of the species groups, we retained this structure and continued to calculate the mean SIMP rate for species groups with rates available and the mean non-SIMP rate for species groups for which rates are available. We then took the difference between the mean SIMP and non-SIMP outcome and stored the value. With over 100 species groups, it was impractical to complete full enumeration of potential permutations to obtain an exact distribution under the null; instead we conducted 20,000 repetitions, which yielded a distribution of the difference between the mean SIMP and non-SIMP outcome (IUU score or mislabeling rate) under the null hypothesis of random assignment of SIMP designation to species group. For any permutations where all the species groups are assigned to either SIMP or non-SIMP, we assign a value of zero difference. Lastly, we determined the p-value for the observed difference in SIMP and non-SIMP mean scores by calculating the percentage of the observations under the null that are greater than the observed value. We report the results for 20,000 repetitions but note that the p-values are identical, up to the digits listed, for runs with 25,000 repetitions. For the IUU score analysis we also reran the analysis replacing blank country and HTS scores with the min instead of the max and observed qualitative similar results, consistent with our main result of failing to reject the null hypothesis that the observed difference equals that which we would expect under random assignment of SIMP status to species groups.

We show the results of the permutation testing for the IUU score in Figure S5 and the mislabeling rate in S6. Each figure includes the distribution we obtained under the null distribution of random assignment of SIMP treatment to the species groups as well as the value associated with the 95th percentile observation and the observed difference.

In addition to the hypothesis tests that appear in the main paper, we conducted several additional tests to examine the robustness of our findings to different assumptions about species groups to include and weights to use.

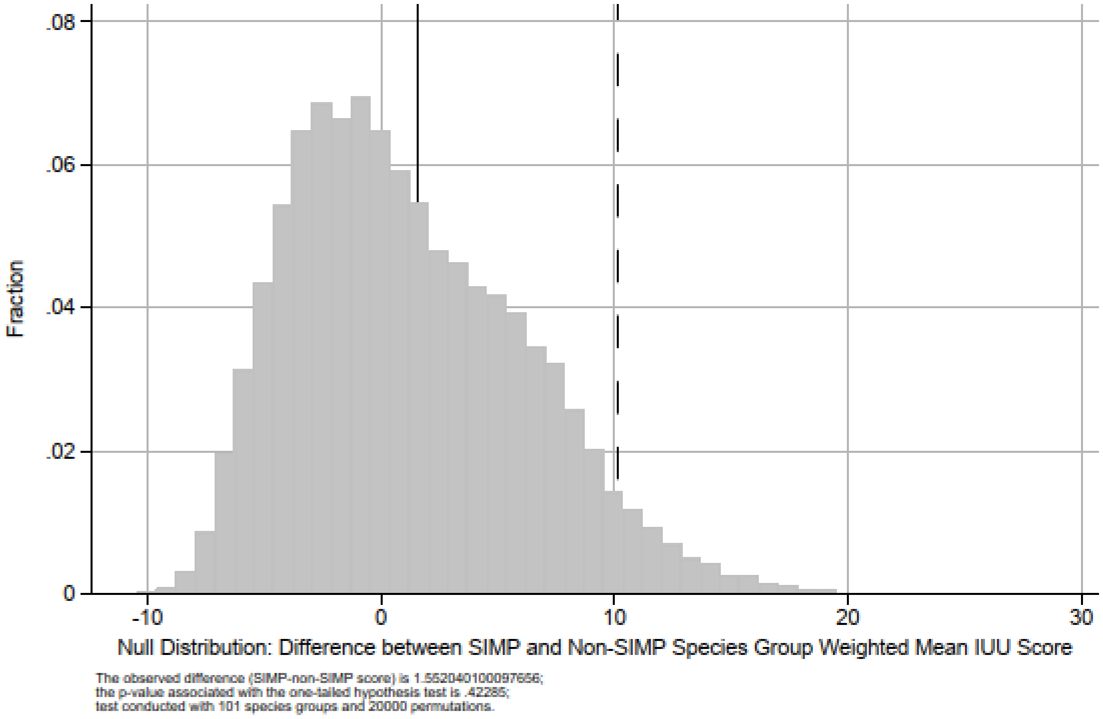

Figure S5: Null distribution and values associated with the IUU hypothesis test. Specifically, the dashed line represents the value associated with the 95th percentile observation of the distribution of the difference between the weighted mean SIMP and non-SIMP IIU score obtained in our 20,000 repetitions of random assignment of SIMP inclusion. We used live weight quantities as weights. The solid line represents the observed mean, 1.55, which is below the 95th percentile of the distribution. The p-value associated with our observed outcome is 0.42 leading us to fail to reject the null hypothesis that the observed difference equals that which we would expect under random assignment of SIMP status to species groups.

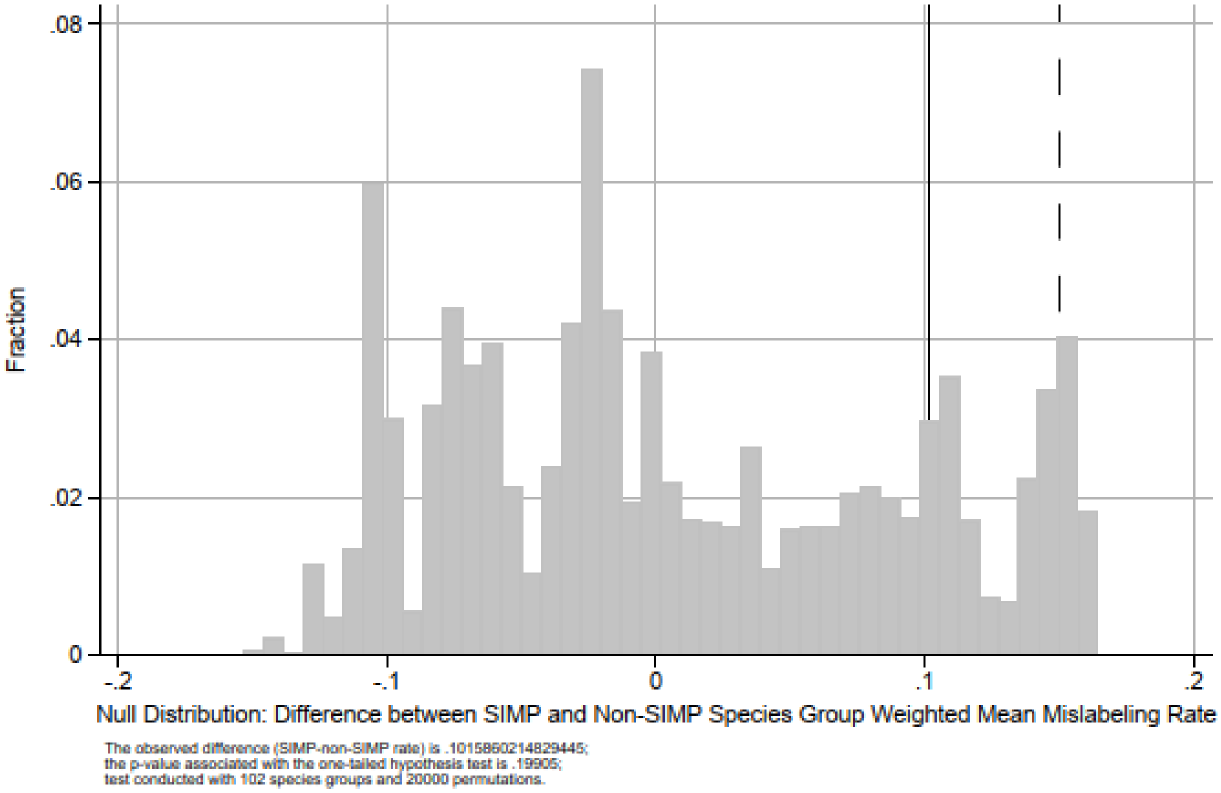

Figure S6: Null distribution and values associated with the mislabeling hypothesis test. Specifically, the dashed line represents the value associated with the 95th percentile observation of the distribution of the difference between the weighted mean SIMP and non-SIMP mislabeling rate obtained in our 20,000 repetitions of random assignment of SIMP inclusion. We used live weight quantities as weights in calculating the means. The solid line represents the observed mean, 0.10, which is below the 95th percentile of the distribution. The p-value associated with our observed outcome is 0.20 leading us to fail to reject the null hypothesis that the observed difference equals that which we would expect under random assignment of SIMP status to species groups.

Robustness checks

We also conduct a series of tests examining whether our findings are robust to focusing on targeting species groups with higher import values.

Raw weight

We repeated our hypothesis tests with raw instead of live weight. We observed a mean IUU score difference between SIMP and non-SIMP species groups of 0.422 with a p-value of 0.52 leading us to reject our null hypothesis that there is not a difference between the SIMP and non-SIMP weighted mean score. For the hypothesis testing of related to mislabeling rates, the observed mean is 0.11 and p-value 0.18 leading us to fail to reject the null hypothesis that the observed difference equals that which we would expect under random assignment of SIMP status to species groups.

Targeting higher-revenue species groups

We are not limited in our data-based approach in terms of species groups we have information on but consider these tests as they may more closely align with the choice set of the initial working group. Specifically, as described in the Presidential Task Force (2015), "Given the large number of seafood species domestically landed and imported, it was not feasible to analyze all species that enter U.S. commerce" and therefore "the Working Group created a base list of species for evaluation using several factors including "the value of domestic landings and imports (all seafood species with an imported or domestically landed value over $100 million USD in 2014 were included." However, other criteria were used including price per pound and expertise of the working group and lower-value species groups were considered. For example, Skipjack Tuna, Dungeness crab., shark, Pacific Whiting, orange roughy, and abalone all had under $20 million in 2016 import value (Table S8) and were examined by the task force (Presidential Task Force, 2015).

We consider the robustness of our results to targeting higher value species groups by restricting the selection of species groups for inclusion to those with at least $1,557,792 in 2016 nominal revenue. We chose a threshold of $1,557,792 because it corresponds to shark, the second lowest in revenue species group designated under SIMP (Table S8). The lowest revenue NMFS species group designated as SIMP is Skipjack Tuna, but it may not have been included if not for the inclusion of other tunas. Specifically, the Final Rule notes a desire "to establish consistent treatment of tuna species, and avoid possible concerns that one species of tuna may be treated differently than others" (NMFS 2016b). In terms of the decision to treat this as a robustness check rather than include a revenue threshold in the main analysis, we note that the total volume under SIMP (either restricting it to some minimum or targeting a threshold) was not mentioned as a goal in species group selection (Presidential Task Force 2105). Furthermore, as described in the main paper and Table S2, government documents are inconsistent on coverage of SIMP and not aligned with data.

When applying the threshold, we do not see a substantial qualitative difference in results for either IUU or mislabeling. The observed difference in weighted mean IUU score between SIMP and non-SIMP species groups was 1.55. We are unable to reject the null hypothesis that the observed difference equals that which we would expect under random assignment of SIMP status to species groups (p-value 0.41). For the analysis related to mislabeling rates, the observed mean is 0.10 and we calculated a p-value of 0.19 leading us to fail to reject the null hypothesis that the observed difference equals that which we would expect under random assignment of SIMP status to species groups.

Targeting high IUU fisheries

For only the IUU score analysis, we conduct a robustness check focused on targeting for SIMP inclusion products from high IUU fisheries, using the measure of IUU relative risk from capture fishing by species group described above. We find that using a score that reflects concentration of IUU product in HTS and country level imports and weights that reflect capture tonnage results in a difference of -0.17 between the weighted SIMP mean score and the weighted non-SIMP score. Because of the need for information on aquaculture tonnage, this score calculation has fewer species groups with both capture tonnage and a weighted capture source for the species group available (see Table S4). In our analysis we drop species groups without information available for the weighted mean and subsequent hypothesis test. Repeating the hypothesis testing, we fail to reject the null hypothesis that the observed difference equals that which we would expect under random assignment of SIMP status to species groups (p-value = 0.53).

IUU Fishing Risk Index

Using our country-scale IUU Fishing Risk Index scores we find that the weighted average SIMP species group score is lower than the weighted average non-SIMP species group score, when we use the live-weight estimates of species group imports from fisheries as weights. The difference in scores is -0.003. We conducted the permutation testing and cannot reject the null hypothesis that the observed difference equals that which we would expect under random assignment of SIMP status to species groups (p-value = 0.47).

Detailed Summary Statistics

Table S9: Summary of 2016 total value and weight subject to some form of SIMP reporting (i.e., treated HTS codes) versus not subject to any SIMP reporting. Percentages of the total are reported in parentheses.

	SIMP	Non-SIMP	Total
Value ($2016)	7,282,846,836 (38%)	11,734,690,222 (62%)	19,017,537,058
Raw Weight (Metric tons)	882,675 (35%)	1,619,929 (65%)	2,502,604
Live Weight (Metric tons)	1,538,338 (33%)	3,170,370 (67%)	4,708,708

Figure S7: Live-weight import volume for top NMFS Species Groups not subject to SIMP

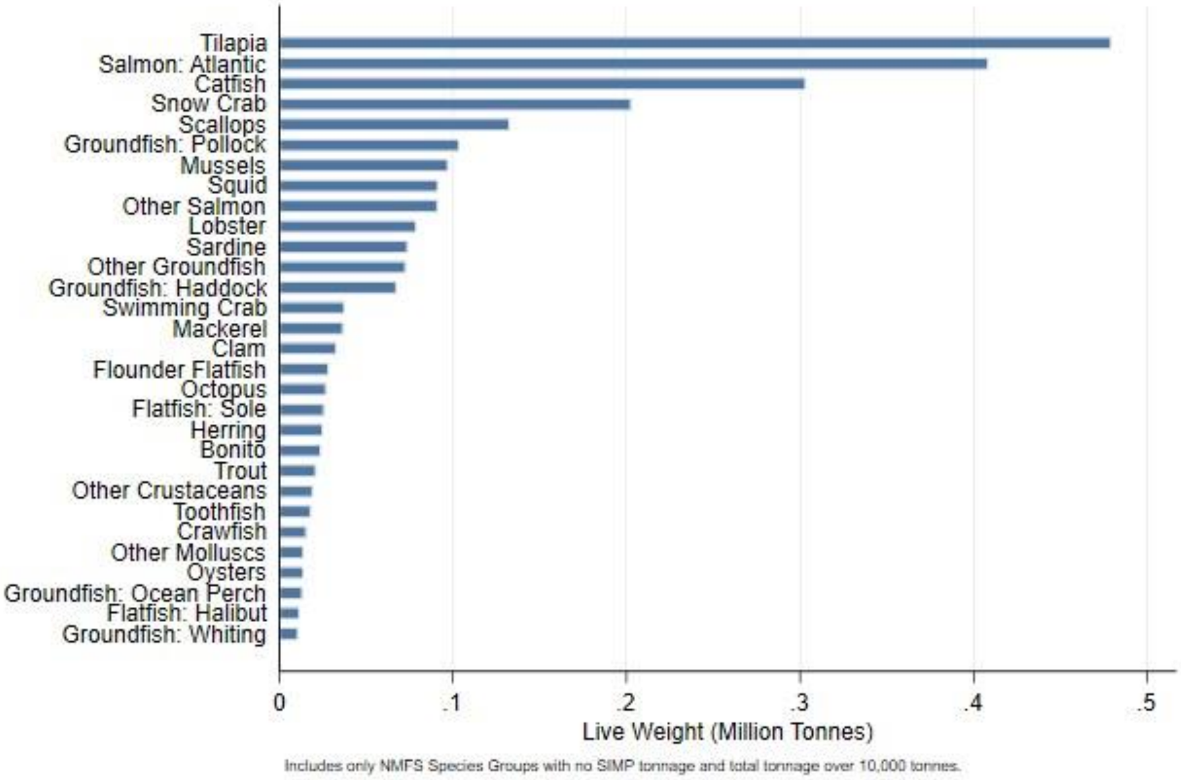

Figure S8: Raw-weight import volume for top NMFS Species Groups not subject to SIMP.

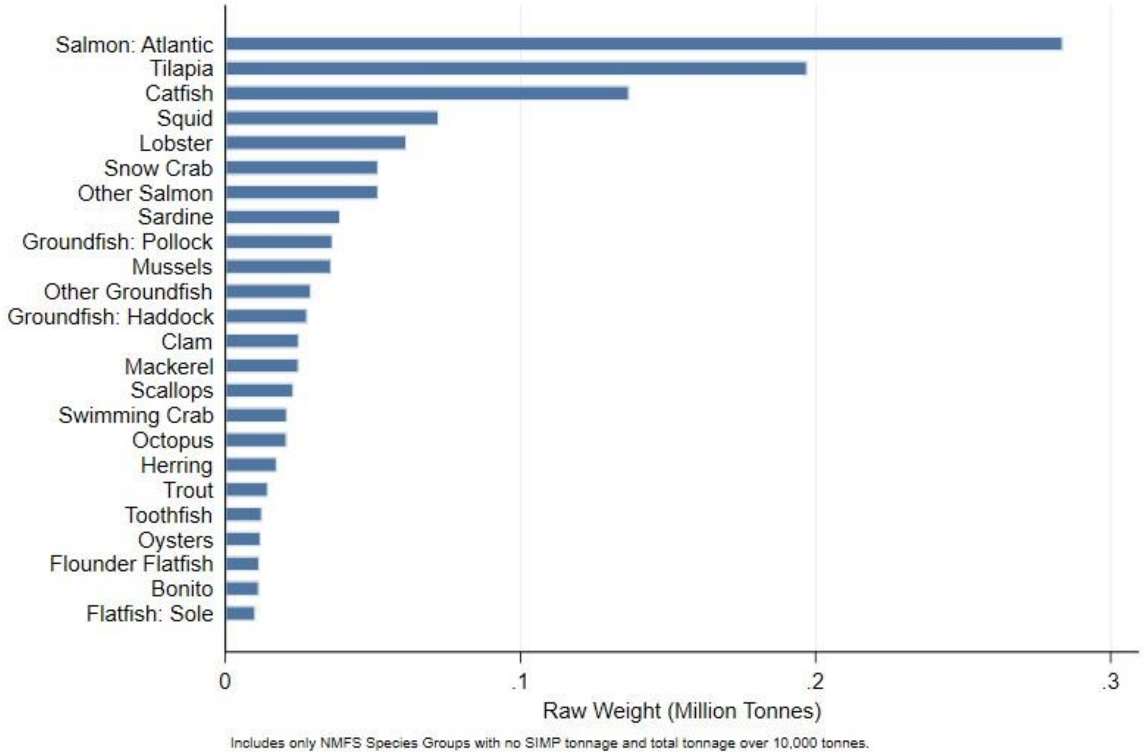

Figure S9: Percentage of NMFS Species Group live-weight import volume covered by SIMP HTS codes, for NMFS Species Groups with nonzero volume covered by SIMP.

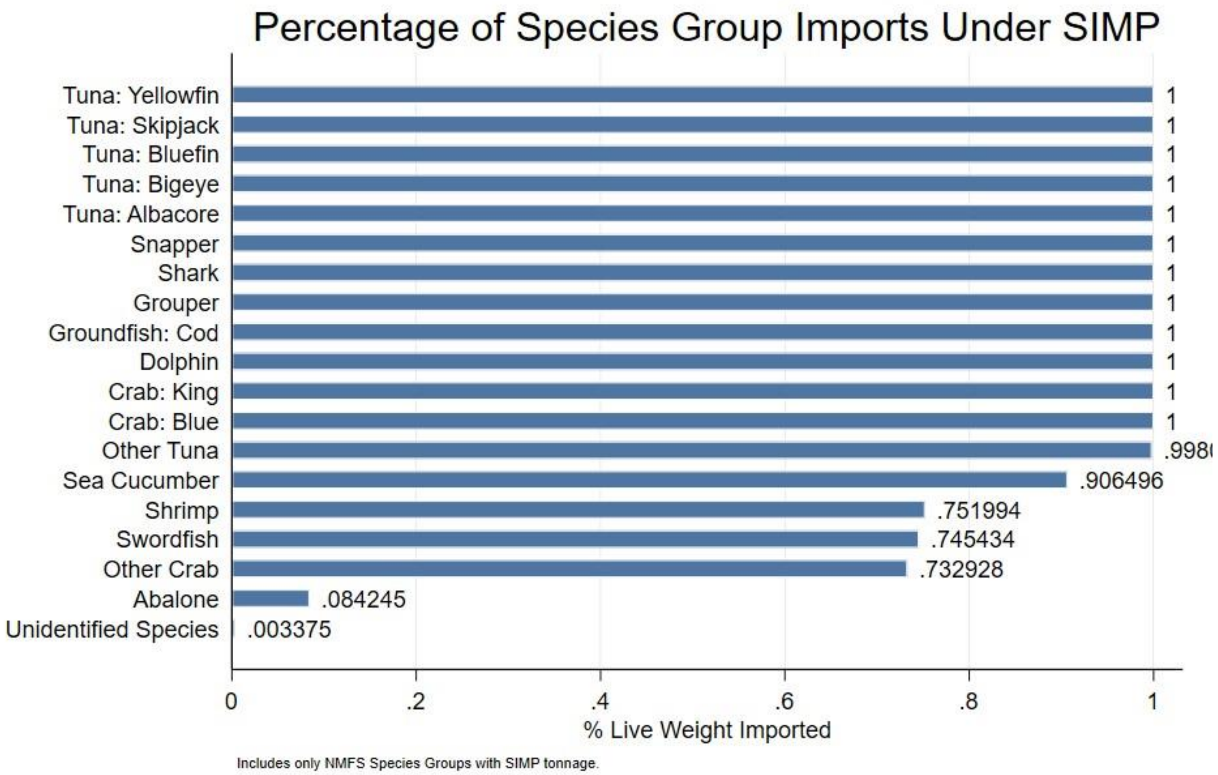

Figure S10: Percentage of NMFS Species Group raw-weight import volume covered by SIMP HTS codes, for NMFS Species Groups with nonzero volume covered by SIMP.

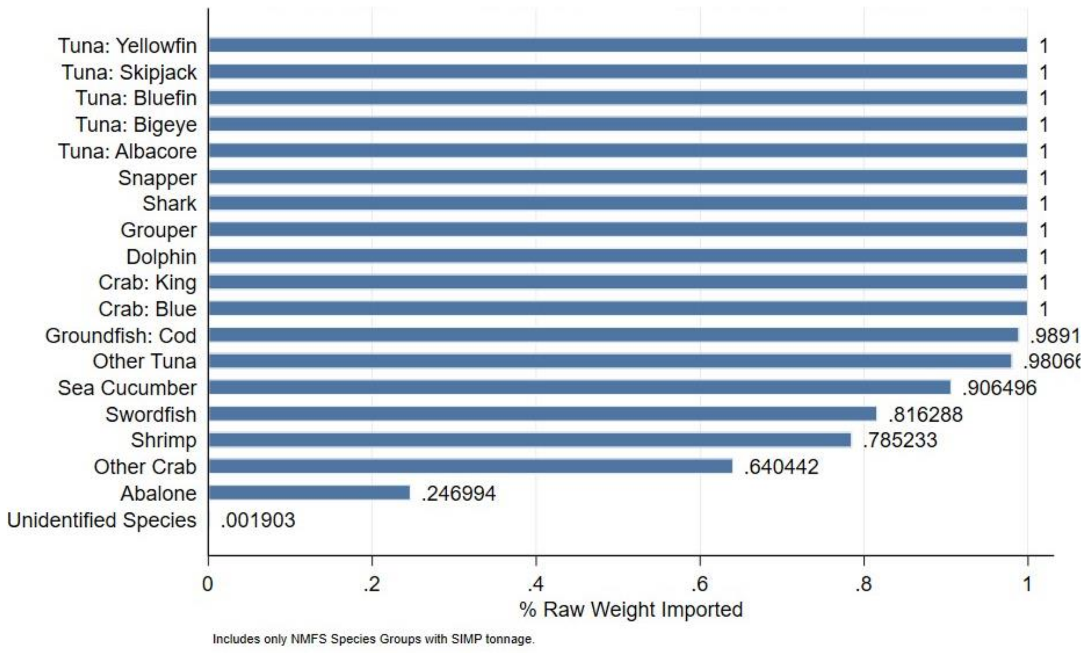

Figure S11: Percentage of NMFS Species Group 2016 import value covered by SIMP HTS codes, for NMFS Species Groups with nonzero value covered by SIMP.

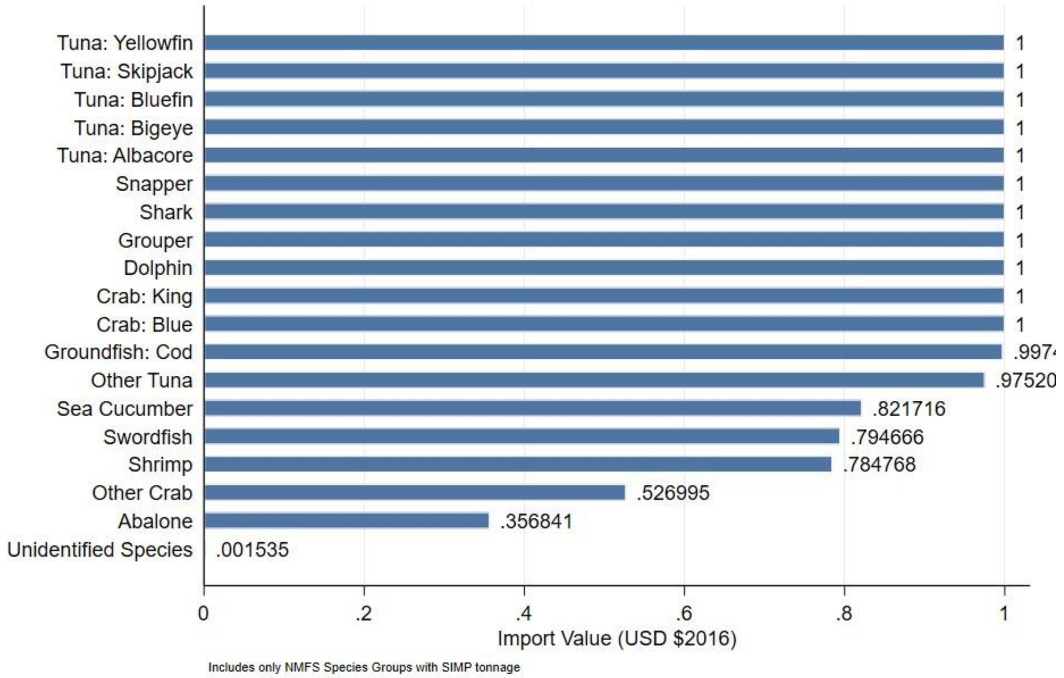

Figure S12: Aggregate estimated raw-weight tonnage and proportion of exports to the U.S. covered by SIMP by country. The proportion of a country's exports to the U.S. covered by SIMP is shown at left. Only exporters of more than 10,000 tonnes of SIMP products are shown.

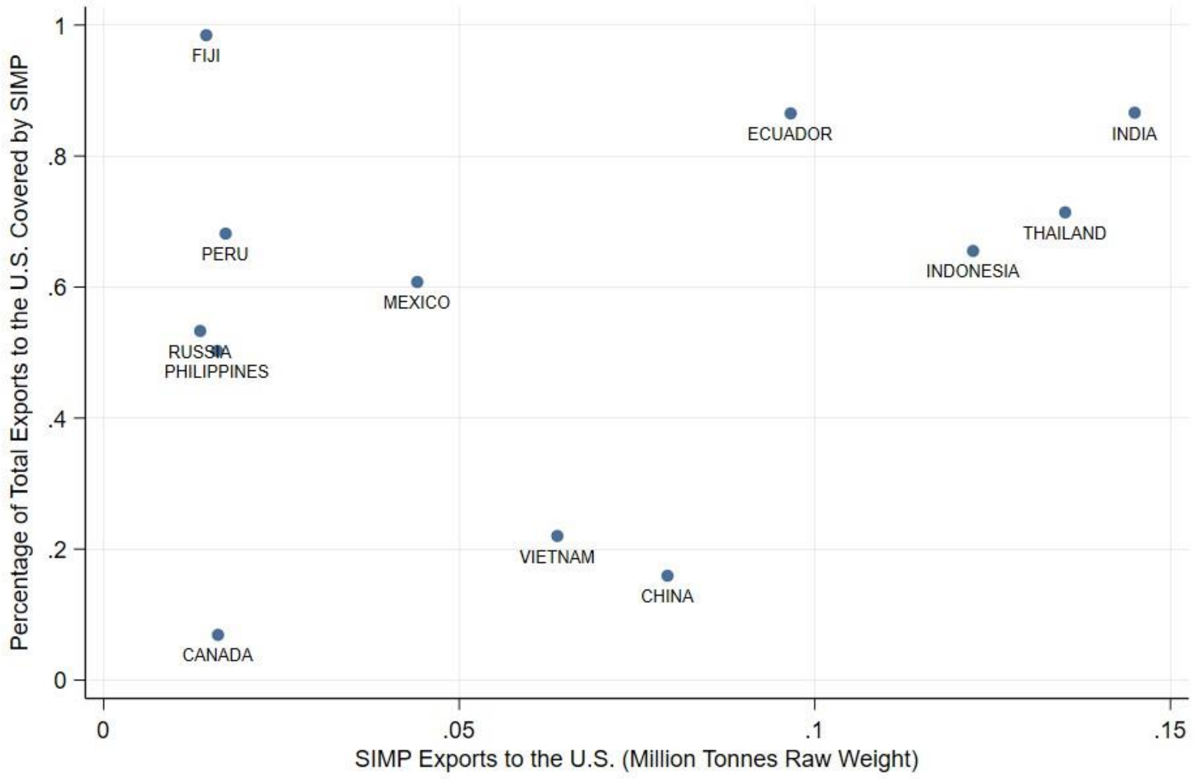

Detailed Data Tables

Table S10: Product names and HTS codes associated with each of the "Other" NMFS species groups as well as the Unidentified species group. A zero in the SIMP field indicated the HTS code is not covered by SIMP; a 1 indicates it is.

Product name	*HTS Code*	*NMFS Species Group*	*SIMP*	*Product Value ($2106)*	*Product Live Weight (tonnes)*	*Product Raw Weight (tonnes)*
AQUATIC INVERTEBRATES NSPF	308900000	Other Aquatic Invertebrates	0	1,834,962	834	167
AQUATIC INVERTEBRATES NSPF PREPARED/PRESERVED	1605690000	Other Aquatic Invertebrates	0	18,534,216	4,417	4,417
CRAB NSPF FROZEN	306144090	Other Crab	1	104,681,094	30,453	11,803
CRAB NSPF LIVE/FRESH/SALTED/BRINE	306244000	Other Crab	0	13,589,136	901	901
CRAB NSPF OTHER PREPARATIONS	1605106090	Other Crab	0	4,095,492	1,145	636
CRAB NSPF OTHER PREPARATIONS IN ATC	1605106010	Other Crab	0	1,054,211	327	182
CRAB PRODUCTS PREPARED DINNERS NOT IN ATC	1605100590	Other Crab	1	1,121,306	235	130
CRABMEAT NSPF FRESH/DRIED/SALTED/BRINE	306242000	Other Crab	0	8,619,868	523	523
CRABMEAT NSPF FROZEN	1605104035	Other Crab	0	19,807,758	2,478	1,377
CRABMEAT NSPF FROZEN	306142000	Other Crab	1	19,807,758	2,478	1,377
CRABMEAT NSPF IN ATC	1605102090	Other Crab	0	43,264,365	4,517	2,510
CRABMEAT NSPF OTHER PREPARATIONS	1605104040	Other Crab	0	24,744,616	3,099	1,722
CRUSTACEANS NSPF FROZEN	306190061	Other Crustaceans	0	9,975,372	1,243	628
CRUSTACEANS NSPF LIVE/FRESH/DRIED/SALTED/ BRINE	306290100	Other Crustaceans	0	617,594	73	73
CRUSTACEANS NSPF PREPARED/PRESERVED	1605401090	Other Crustaceans	0	41,556,564	15,966	6,653

CRUSTACEANS NSPF PRODUCTS PREPARED DINNERS	1605400500	Other Crustaceans	0	3,988,489	1,864	777
FLATFISH NSPF FILLET FRESH	304430090	Other Flatfish	0	11,341,467	1,030	372
FLATFISH NSPF FILLET FROZEN	304835090	Other Flatfish	0	3,658,584	2,132	805
FLATFISH NSPF FRESH	302290190	Other Flatfish	0	2,615,112	381	347
FLATFISH NSPF FROZEN	303390195	Other Flatfish	0	4,248,595	1,623	1,476
FLATFISH NSPF MEAT FRESH	304590060	Other Flatfish	0	105,271	12	9
GROUNDFISH NSPF FILLET BLOCKS FROZEN > 4.5KG	304791090	Other Groundfish	0	2,919,072	1,823	688
GROUNDFISH NSPF FILLET DRIED/SALTED/BRINE	305320090	Other Groundfish	0	22,462,266	23,742	6,314
GROUNDFISH NSPF FILLET FRESH	304440090	Other Groundfish	0	3,942,105	845	305
GROUNDFISH NSPF FILLET FROZEN	304795000	Other Groundfish	0	53,594,078	44,893	16,941
GROUNDFISH NSPF FRESH	302595090	Other Groundfish	0	197,424	56	48
GROUNDFISH NSPF FRESH NOT >6.8KG	302591100	Other Groundfish	0	35,445	12	10
GROUNDFISH NSPF FROZEN	303690000	Other Groundfish	0	2,443,045	873	656
GROUNDFISH NSPF MEAT FRESH	304530090	Other Groundfish	0	40,847,088	0	3,806
GROUNDFISH NSPF MEAT FROZEN NOT > 6.8KG	304959000	Other Groundfish	0	42,278	0	30
GROUNDFISH NSPF MINCED FROZEN > 6.8KG	304951015	Other Groundfish	0	464,181	423	318
MOLLUSCS NSPF FROZEN/DRIED/SALTED/BRINE	307990100	Other Molluscs	0	23,989,058	11,031	2,206
MOLLUSCS NSPF LIVE/FRESH	307910190	Other Molluscs	0	781,163	160	160
MOLLUSCS NSPF PREPARED/PRESERVED	1605596000	Other Molluscs	0	7,606,181	2,383	1,752
MOLLUSCS NSPF PRODUCTS PREPARED DINNERS	1605590500	Other Molluscs	0	1,020,111	402	295

SALMON FILLET BLOCKS FROZEN > 4.5KG	304811000	Other Salmon	0	39,036,392	9,377	5,210
SALMON NSPF CANNED IN OIL	1604112090	Other Salmon	0	1,072,938	396	260
SALMON NSPF CANNED NOT IN OIL	1604114040	Other Salmon	0	4,468,103	851	560
SALMON NSPF FILLET FRESH	304410090	Other Salmon	0	12,964,479	3,246	2,029
SALMON NSPF FILLET FROZEN	304815090	Other Salmon	0	202,702,051	57,841	32,134
SALMON NSPF FRESH	302130062	Other Salmon	0	7,652,389	1,003	879
SALMON NSPF MEAT FRESH	304520020	Other Salmon	0	1,805,535	0	226
SALMON NSPF PREPARED/PRESERVED	1604114050	Other Salmon	0	38,762,914	6,992	4,600
SALMON NSPF SALTED	305694000	Other Salmon	0	159,688	33	22
SALMON PACIFIC NSPF FROZEN	303120062	Other Salmon	0	352,598	40	30
SALMON SMOKED	305410000	Other Salmon	0	107,207,470	11,086	5,279
SALMONIDAE NSPF FRESH	302190000	Other Salmon	0	1,818,809	214	187
SALMONIDAE NSPF FROZEN	303190100	Other Salmon	0	224,218	119	91
SALMONIDAE NSPF MEAT FRESH	304520090	Other Salmon	0	4,305,111	0	330
ANALOG PRODUCT CONTAINING SHELLFISH	1604200590	Other Shellfish	0	3,720,788	2,079	1,034
TUNA NSPF FILLET FROZEN	304870000	Other Tuna	1	324,170,998	72,650	29,060
TUNA NSPF FRESH	302390200	Other Tuna	0	6,169,852	799	688
TUNA NSPF FROZEN	303490200	Other Tuna	1	998,019	354	305
TUNA NSPF IN ATC (FOIL OR FLEXIBLE) IN OIL	1604141010	Other Tuna	1	10,132,986	3,913	1,881
TUNA NSPF IN ATC (FOIL OR FLEXIBLE) NOT IN OIL OVER QUOTA	1604143091	Other Tuna	1	140,335,366	60,919	29,288
TUNA NSPF IN ATC (OTHER) IN OIL	1604141099	Other Tuna	1	21,708,505	11,122	5,347
TUNA NSPF IN ATC (OTHER) NOT IN OIL OVER QUOTA	1604143099	Other Tuna	1	222,441,276	141,442	68,001
TUNA NSPF MEAT FROZEN > 6.8KG	304991090	Other Tuna	0	19,574,870	0	3,141
TUNA NSPF NOT IN A.T.C. NOT IN OIL > 6.8KG	1604144000	Other Tuna	1	290,154,400	125,084	60,137

TUNA NSPF NOT IN A.T.C. NOT IN OIL NOT > 6.8KG	1604145000	Other Tuna	1	2,785,098	351	169
CARP,CATFISH,EELS,NILE PERCH,SNAKEHEAD,TILAPIA FILLET DRIED/SALTED/BRINE	305310000	Unidentified Species	0	7,501,172	7,920	2,106
CARP,CATFISH,EELS,NILE PERCH,SNAKEHEAD,TILAPIA SALTED > 6.8 KG	305645000	Unidentified Species	0	49,204	28	15
CARP,CATFISH,EELS,NILE PERCH,SNAKEHEAD,TILAPIA SALTED NOT > 6.8KG	305641000	Unidentified Species	0	93,312	25	14
CARP,CATFISH,EELS,NILE PERCH,SNAKEHEAD,TILAPIA SMOKED	305440000	Unidentified Species	0	24,638	10	3
CARP,CATFISH,EELS,SNAKEHEAD MEAT FRESH	304510090	Unidentified Species	0	39,443	6	6
CARP,CATFISH,EELS,SNAKEHEAD,TILAPIA FROZEN NOT > 6.8KG	304939000	Unidentified Species	0	91,872	32	32
CARP,CATFISH,EELS,SNAKEHEAD,TILAPIA MEAT FROZEN > 6.8KG	304931090	Unidentified Species	0	322,330	734	143
CARP,CATFISH,EELS,SNAKEHEAD,TILAPIA MINCED FROZEN > 6.8KG	304931010	Unidentified Species	0	2,837	11	2
CARP,EELS,SNAKEHEAD FILLET FRESH	304390000	Unidentified Species	0	1,121,243	374	151
CARP,EELS,SNAKEHEAD FILLET FROZEN	304690000	Unidentified Species	0	11,372,170	4,919	2,216
CLAM,COCKLES,ARK SHELLS NSPF PRODUCTS PREPARED DINNERS	1605560500	Unidentified Species	0	30,343	5	4
CUTTLEFISH,SQUID PRODUCTS PREPARED DINNERS	1605540500	Unidentified Species	0	949,822	287	211
FISH NSPF DRIED	305590000	Unidentified Species	0	17,824,900	6,709	2,103
FISH NSPF FILLET BLOCKS FROZEN > 4.5KG	304891090	Unidentified Species	0	15,472,284	5,651	2,133

FISH NSPF FILLET DRIED/SALTED/BRINE	305396080	Unidentified Species	0	13,993,348	13,935	3,706
FISH NSPF FRESH SCALED NOT >6.8KG	302891140	Unidentified Species	0	60,546	20	17
FISH NSPF HEADS,TAILS,MAWS DRIED/SALTED/BRINE/SMOKED	305720000	Unidentified Species	0	565,971	240	64
FISH NSPF IN ATC IN OIL	1604193100	Unidentified Species	0	3,393,353	752	459
FISH NSPF IN ATC NOT IN OIL	1604192100	Unidentified Species	0	7,480,702	1,980	1,207
FISH NSPF MINCED FROZEN > 6.8KG	304991109	Unidentified Species	0	5,646,864	0	4,215
FISH NSPF PREPARED DINNERS CONTAINING SHELLFISH	1604200510	Unidentified Species	0	5,752,584	1,599	869
FISH NSPF SALTED > 6.8KG	305696001	Unidentified Species	0	454,914	128	69
FISH NSPF SALTED NOT > 6.8KG	305695001	Unidentified Species	0	2,025,295	583	313
FISH NSPF SMOKED	305494041	Unidentified Species	0	48,101,667	16,732	5,055
FISH NSPF SURIMI	304991104	Unidentified Species	0	1,110,921	1,817	353
FRESHWATER FISH NSPF FILLET FRESH	304490015	Unidentified Species	0	7,821,638	1,926	777
FRESHWATER FISH NSPF FILLET FROZEN	304895030	Unidentified Species	0	8,355,989	3,498	1,575
FRESHWATER FISH NSPF FRESH	302895034	Unidentified Species	0	2,595,166	937	837
FRESHWATER FISH NSPF FRESH	302795076	Unidentified Species	0	2,595,166	937	837
FRESHWATER FISH NSPF FROZEN	303890043	Unidentified Species	0	24,572,246	6,750	6,027
FRESHWATER FISH NSPF FROZEN	303290150	Unidentified Species	0	24,572,246	6,750	6,027
FRESHWATER FISH NSPF MEAT FRESH	304590035	Unidentified Species	0	1,596,154	0	421

FRESHWATER FISH NSPF MEAT FROZEN > 6.8KG	304991184	Unidentified Species	0	1,069,549	0	658
FRESHWATER FISH NSPF MEAT FROZEN NOT > 6.8KG	304999192	Unidentified Species	0	1,675,994	0	313
GROUNDFISH COD,CUSK,HADDOCK,HAKE, POLLOCK SMOKED	305494020	Unidentified Species	1	1,234,010	836	253
GROUNDFISH CUSK,HADDOCK FILLET SALTED	305691049	Unidentified Species	0	36,224	16	6
GROUNDFISH CUSK,HADDOCK WHOLE/DRESSED SALTED	305691029	Unidentified Species	0	1,213,452	321	167
GROUNDFISH HAKE,WHITING FROZEN	303660000	Unidentified Species	0	3,331,261	2,297	1,531
MARINE FISH NSPF FILLET FRESH	304490090	Unidentified Species	0	77,666,033	20,289	7,325
MARINE FISH NSPF FILLET FROZEN	304895090	Unidentified Species	0	242,300,120	64,623	24,386
MARINE FISH NSPF FRESH	302895076	Unidentified Species	0	54,819,506	10,663	9,114
MARINE FISH NSPF FROZEN	303890079	Unidentified Species	0	141,329,735	56,690	42,624
MARINE FISH NSPF MEAT FRESH	304590090	Unidentified Species	0	28,661,402	3,339	2,402
MARINE FISH NSPF MEAT FROZEN > 6.8KG	304991194	Unidentified Species	0	3,630,556	0	566
MARINE FISH NSPF MEAT FROZEN NOT > 6.8 KG	304999191	Unidentified Species	0	4,537,216	0	1,845
PERCH FRESHWATER NSPF FRESH	302895025	Unidentified Species	0	1,216,902	552	493
PERCH,PIKE PERCH,YELLOW PIKE FROZEN	303890031	Unidentified Species	0	653,025	360	271
PIKE PERCH,YELLOW PIKE FILLET FROZEN	304895006	Unidentified Species	0	33,520,563	5,569	2,102
PIKE PERCH,YELLOW PIKE FRESH	302895013	Unidentified Species	0	145,586	21	18
PIKE PERCH,YELLOW PIKE MEAT FRESH	304590006	Unidentified Species	0	490,285	60	43

PIKE,PICKEREL FROZEN	303890028	Unidentified Species	0	344,860	136	102
SHAD,STURGEON FRESH	302895049	Unidentified Species	0	2,088	0	0
SHAD,STURGEON FROZEN	303890013	Unidentified Species	0	80,593	26	19
WHITEFISH FILLET FRESH	304490009	Unidentified Species	0	1,097,321	275	99
WHITEFISH FRESH	302895031	Unidentified Species	0	10,490,150	2,388	2,041
WHITEFISH FROZEN	303890037	Unidentified Species	0	3,763,113	1,195	899
WHITEFISH MEAT FRESH	304590025	Unidentified Species	0	1,944,673	532	382

Table S11: Product names and HTS codes associated with byproducts. We dropped records with HTS codes in this list during the data processing stage.

NMFS Species Group	*HTS Code*	*Product Name*
other nonedible	0001009000	TURTLES LIVE
frogs	0001066000	FROG FRESH/FROZEN
frogs	0001076500	FROG MEAT PREPARED/PRESERVED
fish pastes and sauces	0001130100	FISH PASTES,SAUCES CANNED
fish balls	0001130500	FISH BALLS,CAKES,PUDDING CANNED IN OIL
fish balls	0001130800	FISH BALLS,CAKES,PUDDING CANNED NOT IN OIL NOT > 6.8KG
fish balls	0001130820	FISH BALLS,CAKES,PUDDING CANNED NOT IN OIL NOT > 6.8KG
fish balls	0001131100	FISH BALLS,CAKES,PUDDING CANNED OTHER NOT > 6.8KG
fish balls	0001131500	FISH BALLS,CAKES,PUDDING CANNED OTHER > 6.8KG
sticks	0001132000	STICKS,TYPE PRODUCTS COATED NOT COOKED/NOT IN OIL
sticks	0001132100	STICKS,TYPE PRODUCTS COATED
sticks	0001132500	STICKS,TYPE PRODUCTS COATED COOKED/OIL
fish balls	0001138020	FISH BALLS,CAKES,PUDDING,PASTES,SAUCES CANNED
fish pastes and sauces	0001138020	FISH BALLS,CAKES,PUDDING,PASTES,SAUCES CANNED
fish pastes and sauces 2	0001138020	FISH BALLS,CAKES,PUDDING,PASTES,SAUCES CANNED
fish balls	0001138040	FISH BALLS,CAKES,PUDDING,PASTES,SAUCES FRESH/FROZEN
fish pastes and sauces	0001138040	FISH BALLS,CAKES,PUDDING,PASTES,SAUCES FRESH/FROZEN
fish pastes and sauces 2	0001138040	FISH BALLS,CAKES,PUDDING,PASTES,SAUCES FRESH/FROZEN
fish pastes and sauces	0001142000	CRABMEAT NSPF CANNED INCLUDES PASTES/SAUCES
fish oil	0001770200	COD LIVER OIL/FRACTIONS
fish oil	0001770400	FISH NSPF LIVER OIL/FRACTIONS
fish oil	0001771200	ANCHOVY OIL/FRACTIONS
fish oil	0001771400	COD OIL/FRACTIONS
fish oil	0001771600	SHARK OIL/FRACTIONS
fish oil	0001772000	EULACHON OIL/FRACTIONS
fish oil	0001772200	HERRING OIL/FRACTIONS
fish oil	0001772400	MENHADEN OIL/FRACTIONS
fish oil	0001772600	FISH NSPF OIL/FRACTIONS
fish oil	0001773000	SEAL OIL/FRACTIONS
fish oil	0001773200	WHALE SPERM OIL/FRACTIONS CRUDE
fish oil	0001773400	WHALE SPERM OIL/FRACTIONS REFINED
fish oil	0001773600	WHALE NSPF OIL/FRACTIONS
fish oil	0001774000	MARINE ANIMAL NSPF OIL/FRACTIONS
fish oil	0001774500	MARINE ANIMAL NSPF OIL/FRACTIONS

fish oil	0001780500	SOD OIL/FRACTIONS
other edible	0001820500	ANTIPASTO
fish solubles	0001845400	COD LIVER SOLUBLES
animal feed	0001845510	FISH,WHALEMEAT CANNED UNFIT FOR HUMAN CONSUMPTION
animal feed	0001845530	FISH,SHELLFISH MEAL UNFIT FOR HUMAN CONSUMPTION
fish meal	0001845530	FISH,SHELLFISH MEAL UNFIT FOR HUMAN CONSUMPTION
fish solubles	0001845540	FISH NSPF SOLUBLES
animal feed	0001845550	FISH,WHALE PRODUCTS UNFIT FOR HUMAN CONSUMPTION
animal feed	0001845700	FISH,SHELLFISH MEAL UNFIT FOR HUMAN CONSUMPTION
fish meal	0001845700	FISH,SHELLFISH MEAL UNFIT FOR HUMAN CONSUMPTION
other nonedible	0001904700	FISH SCALES CRUDE
fish glue	0001905000	FISH SOUNDS
other nonedible	0001906500	SHELLS MARINE CRUDE
sponges	0001908500	SPONGE GRASS/VELVET/YELLOW
sponges	0001908700	SPONGE SHEEPS WOOL
sponges	0001909000	SPONGE HARDHEAD/REEF
sponges	0001909300	SPONGE NSPF
seaweed nonedible	0001920520	SEAWEED CARAGEENAN CRUDE/GROUND/PULVERIZED
seaweed nonedible	0001920540	SEAWEED NSPF CRUDE/GROUND/PULVERIZED
seaweed nonedible	0001920600	SEAWEED CARAGEENAN CRUDE/GROUND/PULVERIZED
seaweed nonedible	0001920700	SEAWEED CARAGEENAN
fish oil	0004111010	WHALE SPERM OIL/FRACTIONS
agar agar	0004550200	AGAR AGAR
fish glue	0004553600	FISH GLUE VALUED NOT > $0.88/KG
fish glue	0004553800	FISH GLUE VALUED > $0.88/KG
ambergris	0004601000	AMBERGRIS
other nonedible	0004738200	PEARL ESSENCE
other nonedible	0004940600	WAX SPERMACETI
sponges	0007927000	SPONGE NATURAL ARTICLES
frogs	0208200000	FROG LEGS FRESH/FROZEN
reptile	0208500000	REPTILE FRESH/FROZEN
frogs	0208902500	FROG LEGS FRESH/FROZEN
reptile	0210930000	REPTILE MEAT DRIED/SALTED/BRINE/SMOKED
animal feed	0305100000	FISH MEAL FOR HUMAN CONSUMPTION
animal feed	0305102000	FISH MEAL FOR HUMAN CONSUMPTION > 6.8KG
animal feed	0305104000	FISH MEAL FOR HUMAN CONSUMPTION NOT > 6.8KG

animal feed	0309100500	FISH MEAL FOR HUMAN CONSUMPTION > 6.8KG
animal feed	0309109000	FISH MEAL FOR HUMAN CONSUMPTION NOT > 6.8KG
animal feed	0309909000	FISH OR SHELLFISH MEAL FOR HUMAN CONSUMPTION NSPF
sponges	0509000000	SPONGE NATURAL
ambergris	0510002000	AMBERGRIS MAY INCLUDE CASTOREUM,CIVET,MUSK
animal feed	0511910000	FISH,SHELLFISH PRODUCTS UNFIT FOR HUMAN CONSUMPTION
animal feed	0511910090	FISH,SHELLFISH PRODUCTS UNFIT FOR HUMAN CONSUMPTION
sponges	0511993600	SPONGE NATURAL
agar agar	1302310000	AGAR AGAR
seaweed nonedible	1302390000	THICKENERS DERIVED FROM SEAWEED CARAGEENAN
seaweed nonedible	1302390010	THICKENERS DERIVED FROM SEAWEED CARAGEENAN
fish oil	1504100000	FISH NSPF LIVER OIL/FRACTIONS
fish oil	1504102000	COD LIVER OIL/FRACTIONS
fish oil	1504104000	FISH NSPF LIVER OIL/FRACTIONS
fish oil	1504202000	COD OIL/FRACTIONS
fish oil	1504204000	HERRING OIL/FRACTIONS
fish oil	1504206020	MENHADEN OIL/FRACTIONS
fish oil	1504206040	FISH NSPF OIL/FRACTIONS
fish oil	1504207000	FISH NSPF OIL/FRACTIONS
fish oil	1504300000	MARINE ANIMAL NSPF OIL/FRACTIONS
other nonedible	1521904000	WAXES NSPF, MAY INCLUDE SPERMACETI
frogs	1602901000	FROG MEAT
sticks	1604174000	EELS STICKS TYPE PRODUCTS NOT COOKED NOT IN OIL
sticks	1604175000	EELS STICKS TYPE PRODUCTS COOKED OR IN OIL
sticks	1604194000	STICKS,TYPE PRODUCTS COATED NOT COOK NOT IN OIL NOT MINCED
sticks	1604194100	STICKS,TYPE PRODUCTS COATED NOT COOKED NOT IN OIL NOT MINCED
sticks	1604195000	STICKS,TYPE PRODUCTS COATED COOKED OR IN OIL NOT MINCED
sticks	1604195100	STICKS,TYPE PRODUCTS COATED COOKED OR IN OIL NOT MINCED
sticks	1604195500	STICKS,TYPE PRODUCTS COATED NOT MINCED
sticks	1604196000	STICKS,TYPE PRODUCTS NOT COATED IN OIL NOT MINCED > 7KG
sticks	1604196100	STICKS,TYPE PRODUCTS NOT COATED IN OIL NOT MINCED > 7KG
sticks	1604197000	STICKS,TYPE PRODUCTS NOT COATED NOT MINCED
sticks	1604197002	STICKS,TYPE PRODUCTS NOT COATED NOT MINCED
sticks	1604197005	STICKS,TYPE PRODUCTS NOT COATED NOT MINCED
sticks	1604198000	STICKS,TYPE PRODUCTS NOT COATED NOT MINCED NOT > 7KG

sticks	1604198100	STICKS,TYPE PRODUCTS NOT COATED NOT MINCED NOT > 7KG
sticks	1604198200	STICKS,TYPE PRODUCTS NOT COATED NOT MINCED NOT > 7KG
fish pastes and sauces	1604201000	FISH PASTES
fish balls	1604201300	FISH BALLS,CAKES,PUDDING PREPARED/PRESERVED
fish balls	1604201500	FISH BALLS,CAKES,PUDDING IN OIL
fish balls	1604202000	FISH BALLS,CAKES,PUDDING IN ATC NOT IN OIL NOT > 6.8KG
fish balls	1604202500	FISH BALLS,CAKES,PUDDING NOT IN ATC NOT IN OIL NOT > 6.8KG
fish balls	1604203000	FISH BALLS,CAKES,PUDDING NOT IN ATC NOT IN OIL > 6.8KG
sticks	1604204000	STICKS,TYPE PRODUCTS COATED NOT COOKED NOT IN OIL OF MINCED
sticks	1604204500	STICKS,TYPE PRODUCTS COATED OF MINCED
sticks	1604205000	STICKS,TYPE PRODUCTS COATED COOKED OR IN OIL OF MINCED
sticks	1604205010	STICKS,TYPE PRODUCTS COATED COOKED/FROZEN OF MINCED
sticks	1604205090	STICKS,TYPE PRODUCTS COATED NOT COOKED OF MINCED
sticks	1604206000	STICKS,TYPE PRODUCTS NOT COATED OF MINCED
sticks	1604206010	STICKS,TYPE PRODUCTS NOT COATED COOKED/FROZEN OF MINCED
sticks	1604206090	STICKS,TYPE PRODUCTS NOT COATED NOT COOKED OF MINCED
fish pastes and sauces	2103902000	SAUCES DERIVED OR PREPARED FROM FISH
other edible	2104100040	SOUPS,BROTHS BASED ON FISH OR OTHER SEAFOOD
animal feed	2301200000	FISH,SHELLFISH MEAL UNFIT FOR HUMAN CONSUMPTION
fish meal	2301200000	FISH,SHELLFISH MEAL UNFIT FOR HUMAN CONSUMPTION
fish meal	2301200010	HERRING,PILCHARD MEAL UNFIT FOR HUMAN CONSUMPTION
animal feed	2301200090	FISH,SHELLFISH MEAL UNFIT FOR HUMAN CONSUMPTION
fish meal	2301200090	FISH,SHELLFISH MEAL UNFIT FOR HUMAN CONSUMPTION
fish glue	3503001000	FISH GLUE
_other	0004550600	ISINGLASS
species_unidentified	0106900120	LIVE BAIT OTHER THAN WORMS
clam	0001145000	CLAM JUICE CANNED
clam	1603001000	CLAM JUICE
cuttlefish	0001903500	CUTTLEFISH BONE

sturgeon	0001133000	STURGEON ROE FRESH/FROZEN PREPARED/PRESERVED
species_unidentified	0001133500	FISH NSPF ROE BOILED/CANNED
species_unidentified	0001134000	FISH NSPF ROE FRESH/FROZEN
species_unidentified	0001134500	FISH NSPF ROE
herring	0001134510	HERRING ROE
pollock_groundfish	0001134520	GROUNDFISH POLLOCK NSPF ROE
other_salmon	0001134530	SALMON NSPF ROE
sea urchin	0001134540	SEA URCHIN ROE
species_unidentified	0001134590	FISH NSPF ROE
species_unidentified	0302700000	FISH NSPF LIVER,ROE FRESH
sturgeon	0302702000	STURGEON ROE FRESH
species_unidentified	0302704000	FISH NSPF LIVER,ROE FRESH
mullet	0302704010	MULLET ROE FRESH
species_unidentified	0302704090	FISH NSPF LIVER,ROE FRESH
species_unidentified	0302705000	FISH NSPF LIVER,ROE FRESH
sturgeon	0302902000	STURGEON ROE FRESH
mullet	0302904010	MULLET ROE FRESH
species_unidentified	0302904090	FISH NSPF LIVER,ROE FRESH
species_unidentified	0302905000	FISH NSPF LIVER,ROE FRESH
sturgeon	0302912000	STURGEON ROE FRESH
mullet	0302914010	MULLET ROE FRESH
species_unidentified	0302914090	FISH NSPF LIVER,ROE, MILT FRESH
species_unidentified	0302915000	FISH NSPF LIVER,ROE, MILT FRESH
sturgeon	0303802000	STURGEON ROE FROZEN
herring	0303804020	HERRING ROE FROZEN
other_salmon	0303804040	SALMON NSPF ROE FROZEN
pollock_groundfish	0303804050	GROUNDFISH POLLOCK ALASKA ROE FROZEN
mullet	0303804060	MULLET ROE FROZEN
species_unidentified	0303804080	FISH NSPF LIVER,ROE FROZEN
species_unidentified	0303804085	FISH NSPF LIVER,ROE FROZEN
species_unidentified	0303804095	FISH NSPF LIVER,ROE FROZEN
sturgeon	0303902000	STURGEON ROE FROZEN
herring	0303904020	HERRING ROE FROZEN
other_salmon	0303904040	SALMON NSPF ROE FROZEN
pollock_groundfish	0303904050	GROUNDFISH POLLOCK ALASKA ROE FROZEN
mullet	0303904060	MULLET ROE FROZEN
species_unidentified	0303904095	FISH NSPF LIVER,ROE FROZEN
sturgeon	0303912000	STURGEON ROE FROZEN
herring	0303914020	HERRING ROE FROZEN
other_salmon	0303914040	SALMON NSPF ROE FROZEN
pollock_groundfish	0303914050	GROUNDFISH POLLOCK ALASKA ROE FROZEN
mullet	0303914060	MULLET ROE FROZEN

species_unidentified	0303914095	FISH NSPF LIVER,ROE, MILT FROZEN
sturgeon	0305202000	STURGEON ROE CURED
other_salmon	0305204020	SALMON NSPF ROE CURED
herring	0305204040	HERRING ROE CURED
species_unidentified	0305204060	FISH NSPF LIVER,ROE, MILT CURED
species_unidentified	0305204065	FISH NSPF LIVER,ROE, MILT CURED
species_unidentified	0305205000	FISH NSPF LIVER,ROE, MILT CURED
species_unidentified	0305205002	FISH NSPF LIVER,ROE, MILT CURED
sea urchin	0307910021	SEA URCHIN ROE FRESH
sea urchin	0308210021	SEA URCHIN ROE FRESH
krill	0511993030	KRILL ANTARCTIC FOR ANIMAL FEED
oysters	0001144020	OYSTERS SEED
oysters	0307100020	OYSTERS SEED
oysters	0307110020	OYSTERS SEED
shrimp	0001824900	SHRIMP CHIPS
caviar	1604300000	CAVIAR,CAVIAR SUBSTITUTE PREPARED/PRESERVED
caviar	1604302000	CAVIAR
caviar	1604303000	CAVIAR SUBSTITUTE BOILED IN ATC
caviar	1604304000	CAVIAR SUBSTITUTE OTHER PREPARATIONS
caviar	1604310000	CAVIAR
caviar	1604320000	CAVIAR SUBSTITUTE PREPARED/PRESERVED
caviar	1604323000	CAVIAR SUBSTITUTE BOILED IN ATC
caviar	1604324000	CAVIAR SUBSTITUTE OTHER PREPARATIONS
other shellfish	1603009090	FISH,SHELLFISH NSPF JUICE
other shellfish	1603009500	FISH,SHELLFISH NSPF JUICE
seaweed	0001824800	SEAWEED,MARINE PLANTS FOR EDIBLE USE
seaweed	1212200000	SEAWEED AND OTHER ALGAE
seaweed	1212210000	SEAWEED AND OTHER ALGAE FIT FOR HUMAN CONSUMPTION
seaweed	1212290000	SEAWEED AND OTHER ALGAE
coral	0001903000	CORAL CRUDE
coral	0508000000	CORAL,SHELLS,CUTTLEBONE CRUDE/SIMPLY PREPARED
species_unidentified	0511910010	FISH NSPF FERTILIZED EGGS
species_unidentified	1603009010	FISH,SHELLFISH JUICE FROM MEAT
species_unidentified	0302990000	FISH NSPF OTHER EDIBLE OFFAL FRESH
species_unidentified	0303990000	FISH NSPF OTHER EDIBLE OFFAL FROZEN
species_unidentified	0305790000	FISH NSPF OTHER EDIBLE OFFAL
species_unidentified	0309903000	CRUSTACEAN MEAL NSPF DRIED SALTED BRINE
conch	0309905030	CONCH MEAL FROZEN
species_unidentified	0309905090	MOLLUSC MEAL NSPF FROZEN

Table S12: Assignment of 3-alpha codes to the NMFS species groups.

ASFIS 3-alpha code	Common Name (English)	Scientific Name	NMFS Species Group
AAA	adriatic sturgeon	acipenser naccarii	sturgeon
AAB	twobar seabream	acanthopagrus bifasciatus	seabream
AAL	giant mottled eel	anguilla marmorata	eels
AAM	green sturgeon	acipenser medirostris	sturgeon
AAN	fringebarbel sturgeon	acipenser nudiventris	sturgeon
AAQ	new zealand longfin eel	anguilla dieffenbachii	eels
AAR	speckled longfin eel	anguilla reinhardtii	eels
ABZ	small sandeel	ammodytes tobianus	eels
ACH	arctic char	salvelinus alpinus	tilapia
AFQ	pipi wedge clam	paphies australis	clam
AGD	sharktooth moray	gymnothorax maderensis	shark
AGS	spotted sardinella	amblygaster sirm	sardine
AHH	mediterranean sand smelt	atherina hepsetus	smelts
AHN	swallowtail seaperch	anthias anthias	perch nspf
AJM	brine shrimps nei	artemia spp	shrimp
AJQ	common jellyfish	aurelia aurita	jellyfish
AJW	mountain mullet	agonostomus monticola	mullet
AKR	rock bass	ambloplites rupestris	bass
ALK	alaska pollock(=walleye poll.)	gadus chalcogrammus	pollock_groundfish
ALP	alaska plaice	pleuronectes quadrituberculatus	plaice_flatfish
AMS	brine shrimp	artemia salina	shrimp
ANA	argentine anchovy	engraulis anchoita	anchovy
ANB	bay anchovy	anchoa mitchilli	anchovy
ANC	southern african anchovy	engraulis capensis	anchovy
ANE	european anchovy	engraulis encrasicolus	anchovy
ANF	anglerfishes nei	lophiidae	monkfish
ANG	american angler	lophius americanus	monkfish
ANK	blackbellied angler	lophius budegassa	monkfish
ANN	annular seabream	diplodus annularis	seabream
ANR	atlantic sabretooth anchovy	lycengraulis grossidens	anchovy
ANX	anchovies, etc. nei	engraulidae	anchovy
AOM	painted eel	echelus myrus	eels
APB	siberian sturgeon	acipenser baerii	sturgeon
APE	starry sturgeon	acipenser stellatus	sturgeon
APG	danube sturgeon(=osetr)	acipenser gueldenstaedtii	sturgeon
APN	white sturgeon	acipenser transmontanus	sturgeon
APR	sterlet sturgeon	acipenser ruthenus	sturgeon

APU	sturgeon	acipenser sturio	sturgeon
ARE		argentina elongata	herring
ARF	arrowtooth flounder	atheresthes stomias	flounder_flatfish
ARG	argentines	argentina spp	herring
ARK	ark clams nei	arca spp	clam
ARU	greater argentine	argentina silus	herring
ARY	argentine	argentina sphyraena	herring
ASD	allis shad	alosa alosa	shad
ASP	caspian shads	caspialosa spp	shad
ATB	big-scale sand smelt	atherina boyeri	smelts
ATK	okhotsk atka mackerel	pleurogrammus azonus	atka mackerel
ATP	sand smelt	atherina presbyter	smelts
AUD	blacktip sea catfish	arius dussumieri	catfish
AUF	hardhead sea catfish	arius felis	catfish
AUN	giant seacatfish	arius nasutus	catfish
AUR	rough-head sea catfish	arius latiscutatus	catfish
AUX	giant catfish	netuma thalassina	catfish
AVA	atlantic anchoveta	cetengraulis edentulus	anchovy
AVB	western atlantic seabream	archosargus rhomboidalis	seabream
AWE	geelbek croaker	atractoscion aequidens	eels
AWP	gillbacker sea catfish	sciades parkeri	catfish
AWR	softhead sea catfish	amphiarius rugispinis	catfish
AWW	armless snake eel	dalophis imberbis	eels
AWX		arius spp	catfish
AXP	crucifix sea catfish	sciades proops	catfish
BAH	bastard halibut	paralichthys olivaceus	halibut_flatfish
BAJ	japanese seabass	lateolabrax japonicus	sea bass
BAM	mccain's skate	bathyraja maccaini	rays skates
BAP	peruvian rock seabass	paralabrax humeralis	sea bass
BAU	australian bonito	sarda australis	bonito
BAX	bastard halibuts nei	paralichthys spp	halibut_flatfish
BBH	blueback shad	alosa aestivalis	shad
BBM	broad-barred king mackerel	scomberomorus semifasciatus	mackerel
BBS	black scorpionfish	scorpaena porcus	scorpionfish
BCL	butter clam	saxidomus giganteus	clam
BCR	kibonde	chrysichthys brachynema	catfish
BEA	eaton's skate	bathyraja eatonii	rays skates
BEB	coco sea catfish	bagre bagre	catfish
BEC	red sea catfish	bagre pinnimaculatus	catfish
BEI	chilhuil sea catfish	bagre panamensis	catfish
BEM	gafftopsail sea catfish	bagre marinus	catfish
BEP	eastern pacific bonito	sarda chiliensis	bonito

BGJ	bayad	bagrus bajad	catfish
BHY	bathyraja rays nei	bathyraja spp	rays skates
BIC	bighead carp	hypophthalmichthys nobilis	carp
BIP	striped bonito	sarda orientalis	bonito
BJR	roughtail skate	bathyraja trachura	rays skates
BKC	black carp	mylopharyngodon piceus	carp
BKJ	black skipjack	euthynnus lineatus	skipjack_tuna
BLB	blue butterfish	stromateus fiatola	butterfish
BLF	blackfin tuna	thunnus atlanticus	other_tuna
BLL	brill	scophthalmus rhombus	turbot_flatfish
BLS	anadara clams nei	anadara spp	clam
BLT	bullet tuna	auxis rochei	other_tuna
BMU	murray's skate	bathyraja murrayi	rays skates
BOA	bonga shad	ethmalosa fimbriata	shad
BON	atlantic bonito	sarda sarda	bonito
BOP	plain bonito	orcynopsis unicolor	bonito
BPF	kumakuma	brachyplatystoma filamentosum	catfish
BPJ	zebra catfish	brachyplatystoma juruense	catfish
BPT	laulao catfish	brachyplatystoma vaillanti	catfish
BQL	coconut crab	birgus latro	other_crab
BQX	butterfishes nei	stromateus spp	butterfish
BRB	black seabream	spondyliosoma cantharus	seabream
BRD	bearded brotula	brotula barbata	cusk_groundfish
BRS	serra spanish mackerel	scomberomorus brasiliensis	mackerel
BRU	southern rays bream	brama australis	rays skates
BSB	black seabass	centropristis striata	sea bass
BSC	bluespotted seabream	pagrus caeruleostictus	seabream
BSE	seabasses nei	dicentrarchus spp	sea bass
BSR	brazilian sardinella	sardinella brasiliensis	sardine
BSS	european seabass	dicentrarchus labrax	sea bass
BSX	groupers, seabasses nei	serranidae	grouper
BSZ	argentine seabass	acanthistius brasilianus	sea bass
BTG	gulf butterfishes nei	peprilus spp	butterfish
BUT	atlantic butterfish	peprilus triacanthus	butterfish
BUX	butterfishes, pomfrets nei	stromateidae	butterfish
BWB	starry butterfish	stromateus stellatus	butterfish
BXP		brachyplatystoma rousseauxii	catfish
BYE	dark-belly skate	bathyraja meridionalis	rays skates
BYI	sandpaper skate	bathyraja interrupta	rays skates
BYR	kerguelen sandpaper skate	bathyraja irrasa	rays skates
BZB	broadnose skate	bathyraja brachyurops	rays skates
BZM	patagonian skate	bathyraja macloviana	rays skates
BZQ	flounders nei	platichthys spp	flounder_flatfish

BZX	bonitos nei	sarda spp	bonito
CAA	atlantic wolffish	anarhichas lupus	wolffish
CAB	northern wolffish	anarhichas denticulatus	wolffish
CAF	catfishes nei	ictalurus spp	catfish
CAG	glass catfishes	kryptopterus spp	catfish
CAN	naked catfishes	bagrus spp	catfish
CAP	capelin	mallotus villosus	capelin
CAS	spotted wolffish	anarhichas minor	wolffish
CAT	wolffishes(=catfishes) nei	anarhichas spp	wolffish
CAX	sea catfishes nei	ariidae	catfish
CBA	cobia	rachycentron canadum	cobia
CBT	philippine catfish	clarias batrachus	catfish
CDA	threadfin shad	dorosoma petenense	shad
CER	cero	scomberomorus regalis	mackerel
CES	pike icefish	champsocephalus esox	pike
CET	wedge sole	dicologlossa cuneata	sole_flatfish
CEX	cusk-eels nei	genypterus spp	cusk_groundfish
CFS	hong kong catfish	clarias fuscus	catfish
CFW	pompano dolphinfish	coryphaena equiselis	dolphin
CGE	west african geryon	chaceon maritae	other_crab
CGM	africa-bighead catfish, hybrid	clarias gariepinus x c. macrocephalus	catfish
CGZ	conger eels nei	conger spp	eels
CHE	masu(=cherry) salmon	oncorhynchus masou	other_salmon
CHG	chacunda gizzard shad	anodontostoma chacunda	shad
CHI	chinook(=spring=king) salmon	oncorhynchus tshawytscha	chinook_salmon
CHO	cape hope squid	loligo reynaudii	squid
CHP	pacific sardine	sardinops sagax	sardine
CHR	chars nei	salvelinus spp	tilapia
CHU	chum(=keta=dog) salmon	oncorhynchus keta	chum_salmon
CIL	spotted flounder	citharus linguatula	flounder_flatfish
CJM	chilean jack mackerel	trachurus murphyi	horse mackerel_jack
CKI	araucanian herring	strangomera bentincki	herring
CLB	atlantic surf clam	spisula solidissima	clam
CLH	northern quahog(=hard clam)	mercenaria mercenaria	clam
CLI	lingcod	ophiodon elongatus	lingcod
CLM	macha clam	mesodesma donacium	clam
CLN	mudfish	clarias anguillaris	catfish
CLP	herrings, sardines nei	clupeidae	sardine
CLQ	ocean quahog	arctica islandica	clam
CLR	atl.jackknife(=atl.razor clam)	ensis leei	clam

CLT	stimpson's surf clam	spisula polynyma	clam
CLV	venus clams nei	veneridae	clam
CLX	clams, etc. nei	bivalvia	clam
CLZ	north african catfish	clarias gariepinus	catfish
CMA	asian clam	corbicula fluminea	clam
CMG	mrigal carp	cirrhinus mrigala	carp
CMJ	japanese corbicula	corbicula japonica	clam
CMM	manila clam	corbicula manilensis	clam
CMR	mediterranean shore crab	carcinus aestuarii	other_crab
CNS	bloch's gizzard shad	nematalosa nasus	shad
CNU	whitefin wolf-herring	chirocentrus nudus	herring
COA	american conger	conger oceanicus	eels
COE	european conger	conger conger	eels
COH	coho(=silver) salmon	oncorhynchus kisutch	coho_salmon
COM	narrow-barred spanish mackerel	scomberomorus commerson	mackerel
CON	stromboid conchs nei	strombus spp	conch
COO	queen conch	aliger gigas	conch
COS	argentine conger	conger orbignianus	eels
COX	conger eels, etc. nei	congridae	eels
COZ	cockles nei	cardiidae	cockles
CRA	marine crabs nei	brachyura	other_crab
CRC	giant swimcrab	callinectes toxotes	swimming_crab
CRE	edible crab	cancer pagurus	other_crab
CRG	green crab	carcinus maenas	other_crab
CRJ	jonah crab	cancer borealis	other_crab
CRK	atlantic rock crab	cancer irroratus	other_crab
CRQ	queen crab	chionoecetes opilio	snow_crab
CRR	red crab	chaceon quinquedens	other_crab
CRS	portunus swimcrabs nei	portunus spp	swimming_crab
CRW	palinurid spiny lobsters nei	palinurus spp	lobster
CRZ	dana swimcrab	callinectes danae	swimming_crab
CSC	hooded oyster	saccostrea cuccullata	oysters
CSI	slipper cupped oyster	crassostrea iredalei	oysters
CSR	bagrid catfish	chrysichthys nigrodigitatus	catfish
CST	black catfishes nei	chrysichthys spp	catfish
CSY	upsidedown catfishes	synodontis spp	catfish
CTB	common two-banded seabream	diplodus vulgaris	seabream
CTC	common cuttlefish	sepia officinalis	cuttlefish
CTL	cuttlefish, bobtail squids nei	sepiidae, sepiolidae	squid
CTO	torpedo-shaped catfishes nei	clarias spp	catfish

CTR	dwarf bobtail squid	sepiola rondeletii	squid
CUB	black cusk-eel	genypterus maculatus	cusk_groundfish
CUC	red cusk-eel	genypterus chilensis	cusk_groundfish
CUI	caspian shad	alosa caspia	shad
CUL	slender conger	uroconger lepturus	eels
CUS	pink cusk-eel	genypterus blacodes	cusk_groundfish
CVB	tanner crab	chionoecetes bairdi	snow_crab
CVD	pencil urchins	cidaridae	sea urchin
CWC	karanteen seabream	crenidens crenidens	seabream
CWE	mola rock crab	metacarcinus edwardsii	other_crab
CWG	prussian carp	carassius gibelio	carp
CWJ	red snow crab	chionoecetes japonicus	snow_crab
CZJ	blue squat lobster	cervimunida johni	lobster
CZM	right-handed hermit crabs nei	paguridae	other_crab
DAB	common dab	limanda limanda	other_flatfish
DAS	chinese gizzard shad	clupanodon thrissa	shad
DBM	double-lined mackerel	grammatorcynus bilineatus	mackerel
DEA	angolan dentex	dentex angolensis	seabream
DEC	common dentex	dentex dentex	seabream
DEL	large-eye dentex	dentex macrophthalmus	seabream
DEM	morocco dentex	dentex maroccanus	seabream
DEN	canary dentex	dentex canariensis	seabream
DEP	pink dentex	dentex gibbosus	seabream
DES	deep-sea smelt	glossanodon semifasciatus	smelts
DEX	dentex nei	dentex spp	seabream
DGQ	specklefin cusk-eel	lepophidium negropinna	cusk_groundfish
DHZ	ocellated wedge sole	dicologlossa hexophthalma	sole_flatfish
DIG	south american silver porgy	diplodus argenteus	seabream
DIH	spottail seabream	diplodus holbrooki	seabream
DKC	crocus giant clam	tridacna crocea	clam
DKX	elongate giant clam	tridacna maxima	clam
DMI	marble grouper	dermatolepis inermis	grouper
DNC	congo dentex	dentex congoensis	seabream
DOB	dorab wolf-herring	chirocentrus dorab	herring
DOD	dotted gizzard shad	konosirus punctatus	shad
DON	donax clams	donax spp	clam
DOS	wolf-herrings nei	chirocentrus spp	herring
DOT	dogtooth tuna	gymnosarda unicolor	other_tuna
DPG	granulated catfish	pterodoras granulosus	catfish
DPQ	new zealand smooth skate	dipturus innominatus	rays skates
DPV	yellownose skate	dipturus chilensis	rays skates

DQX	gizzard shad nei	dorosoma spp	shad
DTT	yellowback seabream	dentex tumifrons	seabream
DUN	dungeness crab	cancer magister	dungeness_crab
DWS	deep-water sharks nei	elasmobranchii	shark
DYM	atlantic thorny oyster	spondylus americanus	oysters
DZX	thorny oysters nei	spondylus spp	oysters
EAA	regan's anchovy	anchoa argentivittata	anchovy
EAE	eigenmann's anchovy	anchoa eigenmannia	anchovy
EAG	eagle rays nei	myliobatidae	rays skates
EAM	marini's anchovy	anchoa marinii	anchovy
EAN	longnose anchovy	anchoa nasus	anchovy
EAP	panama anchovy	anchoa panamensis	anchovy
EAR	short anchovy	anchoa curta	anchovy
EBS	brilliant pomfret	eumegistus illustris	turbot_flatfish
ECT	shorthead anchovy	encrasicholina heteroloba	anchovy
EDT	musky octopus	eledone moschata	octopus
EIK	warty crab	eriphia verrucosa	other_crab
EJB	african cuttlefish	sepia bertheloti	cuttlefish
EJE	elegant cuttlefish	sepia elegans	cuttlefish
EKK	southern hardshell clam	mercenaria campechiensis	clam
ELA	american eel	anguilla rostrata	eels
ELE	european eel	anguilla anguilla	eels
ELJ	japanese eel	anguilla japonica	eels
ELS	whitespotted conger	conger myriaster	eels
ELU	short-finned eel	anguilla australis	eels
ELX	river eels nei	anguilla spp	eels
END	devis' anchovy	encrasicholina devisi	anchovy
ENF	stridulating stone crab	menippe frontalis	other_crab
ENJ	crimson seabream	evynnis japonica	seabream
ENL	lake malawi sardine	engraulicypris sardella	sardine
ENP	broad-striped anchovy	anchoa hepsetus	anchovy
ENR	anchovies nei	engraulis spp	anchovy
EOI	horned octopus	eledone cirrhosa	octopus
EOJ	petrale sole	eopsetta jordani	sole_flatfish
EOL	southern lemon sole	pelotretis flavilatus	sole_flatfish
EQE	pod razor shell	ensis ensis	clam
EQH	giant jackknife	ensis macha	clam
EQI	sword razor shell	ensis siliqua	clam
EQK	arched razor shell	ensis magnus	clam
ERP	american harvestfish	peprilus paru	butterfish
ERS	chinese mitten crab	eriocheir sinensis	other_crab
ESN	hardenberg's anchovy	stolephorus insularis	anchovy
ESU	common hairfin anchovy	setipinna tenuifilis	anchovy

ESW	spotty-face anchovy	stolephorus waitei	anchovy
ESY	scaly hairfin anchovy	setipinna taty	anchovy
EUL	eulachon	thaleichthys pacificus	smelts
EXF	tiger-dragon grouper, hybrid	e. fuscoguttatus x e. lanceolatus	grouper
EYB	baelama anchovy	thryssa baelama	anchovy
EZS	slender rockfish	scorpaena elongata	ocean perch_groundfish
FAG	rooster venus	paphia gallus	clam
FCC	crucian carp	carassius carassius	carp
FCG	grass carp(=white amur)	ctenopharyngodon idella	carp
FCH	hoven's carp	leptobarbus hoeveni	carp
FCN	nilem carp	osteochilus hasselti	carp
FCP	common carp	cyprinus carpio	carp
FFH	e.pacific black goastshark	hydrolagus melanophasma	shark
FGI	european green frog	rana ridibunda	frog
FGY	pig frog	rana grylio	frog
FHC	seabass	centropristis philadelphica	sea bass
FLD	windowpane flounder	scophthalmus aquosus	flounder_flatfish
FLE	european flounder	platichthys flesus	flounder_flatfish
FLS	summer flounder	paralichthys dentatus	flounder_flatfish
FLT	asian swamp eel	monopterus albus	eels
FLW	winter flounder	pseudopleuronectes americanus	flounder_flatfish
FLX	flatfishes nei	pleuronectiformes	other_flatfish
FOK	american bull frog	rana catesbeiana	frog
FOR	forkbeard	phycis phycis	hake_groundfish
FOX	forkbeards nei	phycis spp	hake_groundfish
FPE	european perch	perca fluviatilis	perch nspf
FPI	northern pike	esox lucius	pike
FPP	pike-perch	sander lucioperca	sauger
FPY	american yellow perch	perca flavescens	yellow perch
FRG	frogs	rana spp	frog
FRI	frigate tuna	auxis thazard	other_tuna
FRZ	frigate and bullet tunas	auxis thazard, a. rochei	other_tuna
FSA	sand flounders nei	rhombosolea spp	flounder_flatfish
FTS	flathead sole	hippoglossoides elassodon	sole_flatfish
FXG	smooth scallop	flexopecten glaber	scallops
GAZ	gazami crab	portunus trituberculatus	other_crab
GDW	gonate squids nei	gonatidae	squid
GER	chaceon geryons nei	chaceon spp	other_crab
GFB	greater forkbeard	phycis blennoides	hake_groundfish
GFZ	chinese mystery snail	cipangopaludina chinensis	snail
GGG	beaked salmon	gonorynchus gonorynchus	other_salmon

GHL	greenland halibut	reinhardtius hippoglossoides	halibut_flatfish
GIP	barramundi(=giant seaperch)	lates calcarifer	perch nspf
GIS	jumbo flying squid	dosidicus gigas	squid
GLZ	rex sole	glyptocephalus zachirus	sole_flatfish
GNP	slobbering catfish	brachyplatystoma platynemum	catfish
GPE	longfin hake	phycis chesteri	hake_groundfish
GRC	greenland cod	gadus ogac	cod_groundfish
GRN	blue grenadier	macruronus novaezelandiae	hake_groundfish
GRO	groundfishes nei	actinopterygii	other_groundfish
GSU	silver seabream	pagrus auratus	seabream
GUT	indo-pacific king mackerel	scomberomorus guttatus	mackerel
GWQ	ringeye conger	paraconger californiensis	eels
GYP	peruvian sea catfish	galeichthys peruvianus	catfish
HAD	haddock	melanogrammus aeglefinus	haddock_groundfish
HAI	indian halibut	psettodes erumei	halibut_flatfish
HAL	atlantic halibut	hippoglossus hippoglossus	halibut_flatfish
HAP	pacific halibut	hippoglossus stenolepis	halibut_flatfish
HBJ	pacific golden crab	chaceon bicolor	other_crab
HBN	southwest atlantic red crab	chaceon notialis	other_crab
HBU	loweye catfishes nei	hypophthalmus spp	catfish
HBY	porthole shovelnose catfish	hemisorubim platyrhynchos	catfish
HBZ	hair crab	erimacrus isenbeckii	other_crab
HCA	asiatic hard clam	meretrix meretrix	clam
HCG	scaled herring	harengula jaguana	herring
HCJ	japanese hard clam	meretrix lusoria	clam
HCL	imperial surf clam	pseudocardium sybillae	clam
HCT	pacific flatiron herring	harengula thrissina	herring
HCX	hard clams nei	meretrix spp	clam
HEB	african catfish	heterobranchus bidorsalis	catfish
HEL	sampa	heterobranchus longifilis	catfish
HEP	pacific herring	clupea pallasii	herring
HER	atlantic herring	clupea harengus	herring
HES	bluestripe herring	herklotsichthys quadrimaculat.	herring
HFB	golden deepsea crab	chaceon fenneri	other_crab
HGL	diamond turbot	pleuronichthys guttulatus	turbot_flatfish
HIL	hilsa shad	tenualosa ilisha	shad
HIP	bear paw clam	hippopus hippopus	clam
HIX	kelee shad	hilsa kelee	shad
HKB	benguela hake	merluccius polli	hake_groundfish
HKC	cape hakes	merluccius capensis, m.paradoxus	hake_groundfish
HKD	blacksaddle herring	herklotsichthys dispilonotus	herring

HKE	european hake	merluccius merluccius	hake_groundfish
HKK	shallow-water cape hake	merluccius capensis	hake_groundfish
HKM	senegalese hake	merluccius senegalensis	hake_groundfish
HKN	southern hake	merluccius australis	hake_groundfish
HKO	deep-water cape hake	merluccius paradoxus	hake_groundfish
HKP	argentine hake	merluccius hubbsi	hake_groundfish
HKR	red hake	urophycis chuss	hake_groundfish
HKS	silver hake	merluccius bilinearis	hake_groundfish
HKU	brazilian codling	urophycis brasiliensis	hake_groundfish
HKW	white hake	urophycis tenuis	hake_groundfish
HKX	hakes nei	merluccius spp	hake_groundfish
HKZ	merluccid hakes nei	merlucciidae	hake_groundfish
HMC	cape horse mackerel	trachurus capensis	horse mackerel_jack
HMG	greenback horse mackerel	trachurus declivis	horse mackerel_jack
HMM	mediterranean horse mackerel	trachurus mediterraneus	horse mackerel_jack
HMZ	cunene horse mackerel	trachurus trecae	horse mackerel_jack
HNG	black sole	achlyopa nigra	sole_flatfish
HNQ	pink geryon	chaceon macphersoni	other_crab
HOF	offshore silver hake	merluccius albidus	hake_groundfish
HOM	atlantic horse mackerel	trachurus trachurus	horse mackerel_jack
HPN	highwaterman catfish	hypophthalmus edentatus	catfish
HSC	horseshoe crab	limulus polyphemus	other_crab
HSL	suckermouth catfish	hypostomus plecostomus	catfish
HTF	stinging catfish	heteropneustes fossilis	catfish
HUH	beluga	huso huso	sturgeon
HUM	atka mackerel	pleurogrammus monopterygius	atka mackerel
HUS	yellow snapper	lutjanus argentiventris	snapper
HVJ	japanese smelt	hypomesus nipponensis	smelts
HWP	giant stone crab	homalaspis plana	other_crab
HXP	silver, bighead carps nei	hypophthalmichthys spp	carp
HYM	zamurito	calophysus macropterus	catfish
IAH	pharaoh cuttlefish	sepia pharaonis	cuttlefish
IAR	pink cuttlefish	sepia orbignyana	cuttlefish
IAX	cuttlefishes nei	sepia spp	cuttlefish
IBA	velvet fan lobster	ibacus alticrenatus	lobster
IBC	japanese fan lobster	ibacus ciliatus	lobster
ICI	true sole	heteromycteris proboscideus	sole_flatfish
IDR	rayed pearl oyster	pinctada radiata	oysters
IIM	splittail bass	hemanthias peruanus	bass
ILF	zucchini catfish	isorineloricaria festae	catfish
ILL	shortfin squids nei	illex spp	squid
IOD	blue-leg swimcrab	liocarcinus depurator	swimming_crab

IOS	indian oil sardine	sardinella longiceps	sardine
IPF	flounders nei	hippoglossina spp	flounder_flatfish
IPL	butterfly perch	caesioperca lepidoptera	perch nspf
IPO	flathead catfish	pylodictis olivaris	catfish
IQL		chrysichthys platycephalus	catfish
IQM		chrysichthys sianenna	catfish
IQN		chrysichthys stappersii	catfish
IRQ	sleek lates	lates stappersii	perch nspf
ISC	iceland scallop	chlamys islandica	scallops
ISI	butter sole	isopsetta isolepis	sole_flatfish
ITC	white catfish	ameiurus catus	catfish
ITE	brown bullhead	ameiurus nebulosus	catfish
ITF	blue catfish	ictalurus furcatus	catfish
ITM	black bullhead	ameiurus melas	catfish
ITP	channel catfish	ictalurus punctatus	catfish
ITW	common bobtail squid	sepietta oweniana	squid
IUB	western atlantic brief squid	lolliguncula brevis	squid
IYI	bigmouth sanddab	citharichthys gilberti	other_flatfish
IYM	speckled sanddab	citharichthys stigmaeus	other_flatfish
IYO	pacific sanddab	citharichthys sordidus	other_flatfish
JAA	blue jack mackerel	trachurus picturatus	horse mackerel_jack
JAD	norwegian skate	dipturus nidarosiensis	rays skates
JAG	velez ray	rostroraja velezi	rays skates
JAI	brown ray	raja miraletus	rays skates
JAJ	southern spider crab	jacquinotia edwardsii	other_crab
JAN	japanese anchovy	engraulis japonicus	anchovy
JAR	rough ray	raja radula	rays skates
JAT	rough skate	dipturus nasutus	rays skates
JAX	jack and horse mackerels nei	trachurus spp	horse mackerel_jack
JAY	speckled ray	raja polystigma	rays skates
JBZ	african bullhead	lophiobagrus cyclurus	catfish
JCX	maja spider crabs nei	maja spp	other_crab
JDV	common spider crab	maja brachydactyla	other_crab
JEL	jellyfishes nei	rhopilema spp	jellyfish
JFE	clearnose skate	rostroraja eglanteria	rays skates
JFQ	ecuatorial ray	rostroraja equatorialis	rays skates
JFR	california ray	beringraja inornata	rays skates
JFV	leopard skate	rajella leopardus	rays skates
JFY	madeiran ray	raja maderensis	rays skates
JJM	japanese jack mackerel	trachurus japonicus	horse mackerel_jack
JPC	chilean sandperch	pinguipes chilensis	perch nspf

JRH	deep-water ray	rajella bathyphila	rays skates
JRI	big skate	beringraja binoculata	rays skates
JRS	mediterranean starry ray	raja asterias	rays skates
JRT	spotback skate	atlantoraja castelnaui	rays skates
JRY	eyespot skate	atlantoraja cyclophora	rays skates
JSC	yesso scallop	mizuhopecten yessoensis	scallops
JSN	southern rock lobster	jasus novaehollandiae	lobster
JSP	st.paul rock lobster	jasus paulensis	lobster
JSS	japanese sardinella	sardinella zunasi	sardine
JTX	conch shells nei	strombidae	conch
KAF	kamchatka flounder	atheresthes evermanni	flounder_flatfish
KAQ	golden king crab	lithodes aequispina	king_crab
KAW	kawakawa	euthynnus affinis	other_tuna
KCA	king crab	lithodes ferox	king_crab
KCF	globose king crab	paralomis formosa	king_crab
KCI	blue king crab	paralithodes platypus	king_crab
KCM	subantarctic stone crab	lithodes murrayi	other_crab
KCP	kingklip	genypterus capensis	cusk_groundfish
KCR	southern king crab	lithodes santolla	king_crab
KCT	stone king crab	lithodes maja	king_crab
KCU	red stone crab	paralomis aculeata	other_crab
KCV	antarctic stone crab	paralomis spinosissima	other_crab
KCX	king crabs, stone crabs nei	lithodidae	king_crab
KCY	brown king crab	paralithodes brevipes	king_crab
KCZ	king crabs nei	lithodes spp	king_crab
KDG	giant land crab	cardisoma guanhumi	other_crab
KDK	mouthless land crab	cardisoma crassum	other_crab
KEF	deep-sea red crab	chaceon affinis	other_crab
KEM	portly spider crab	libinia emarginata	other_crab
KGM	king mackerel	scomberomorus cavalla	mackerel
KGX	seerfishes nei	scomberomorus spp	mackerel
KPC	batwing coral crab	carpilius corallinus	other_crab
KPG	shamefaced crab	calappa granulata	other_crab
KPM	clown crab	carpilius maculatus	other_crab
KRI	antarctic krill	euphausia superba	krill
KRX	antarctic krill nei	euphausia spp	krill
KSI	damsel bass	hemanthias signifer	bass
KSY	klein's sole	synapturichthys kleinii	sole_flatfish
KVV	red vermillion crab	paralomis verrilli	other_crab
LAT	lake trout(=char)	salvelinus namaycush	trout
LBA	american lobster	homarus americanus	lobster
LBC	cape rock lobster	jasus lalandii	lobster
LBE	european lobster	homarus gammarus	lobster

LBS	homarus lobsters nei	homarus spp	lobster
LBT	tristan da cunha rock lobster	jasus tristani	lobster
LDB	four-spot megrim	lepidorhombus boscii	other_flatfish
LEF	lefteye flounders nei	bothidae	flounder_flatfish
LEM	lemon sole	microstomus kitt	sole_flatfish
LEZ	megrims nei	lepidorhombus spp	other_flatfish
LIO	velvet swimcrab	necora puber	swimming_crab
LJA	african red snapper	lutjanus agennes	snapper
LJB	two-spot red snapper	lutjanus bohar	snapper
LJC	colorado snapper	lutjanus colorado	snapper
LJE	african brown snapper	lutjanus dentatus	snapper
LJF	dory snapper	lutjanus fulviflamma	snapper
LJG	humpback red snapper	lutjanus gibbus	snapper
LJH	john's snapper	lutjanus johnii	snapper
LJI	grey snapper	lutjanus griseus	snapper
LJJ	dog snapper	lutjanus jocu	snapper
LJK	one-spot snapper	lutjanus monostigma	snapper
LJM	mahogany snapper	lutjanus mahogoni	snapper
LJN	mutton snapper	lutjanus analis	snapper
LJO	gorean snapper	lutjanus goreensis	snapper
LJP	schoolmaster snapper	lutjanus apodus	snapper
LJR	mullet snapper	lutjanus aratus	snapper
LJS	spotted rose snapper	lutjanus guttatus	snapper
LJT	crimson snapper	lutjanus erythropterus	snapper
LJU	blackfin snapper	lutjanus buccanella	snapper
LJV	blacktail snapper	lutjanus fulvus	snapper
LJW	pacific dog snapper	lutjanus novemfasciatus	snapper
LJY	cubera snapper	lutjanus cyanopterus	snapper
LLZ	chinese longsnout catfish	leiocassis longirostris	catfish
LMS	mud spiny lobster	panulirus polyphagus	lobster
LOA	australian spiny lobster	panulirus cygnus	lobster
LOF	juan fernandez rock lobster	jasus frontalis	lobster
LOG	green rock lobster	jasus verreauxi	lobster
LOJ	longlegged spiny lobster	panulirus longipes	lobster
LOK	scalloped spiny lobster	panulirus homarus	lobster
LOQ	craylets, squat lobsters nei	galatheidae	lobster
LOR	red rock lobster	jasus edwardsii	lobster
LOS	slipper lobsters nei	scyllaridae	lobster
LOT	longtail tuna	thunnus tonggol	other_tuna
LOX	lobsters nei	reptantia	lobster
LOY	royal spiny lobster	panulirus regius	lobster

LQL	pelagic red crab	pleuroncodes planipes	other_crab
LRN	blunt-toothed african catfish	clarias ngamensis	catfish
LSL	forktail lates	lates microlepis	perch nspf
LSM	bigeye lates	lates mariae	perch nspf
LSV	herring scad	alepes vari	herring
LTA	little tunny(=atl.black skipj)	euthynnus alletteratus	other_tuna
LTG	tanganyika lates	lates angustifrons	perch nspf
LTJ	silk snapper	lutjanus vivanus	snapper
LTM	lake tanganyika sardine	limnothrissa miodon	sardine
LUB	emperor red snapper	lutjanus sebae	snapper
LUF	yellow-lined snapper	lutjanus rufolineatus	snapper
LUJ	brownstripe red snapper	lutjanus vitta	snapper
LVG	papuan black snapper	lutjanus goldiei	snapper
LVK	common bluestripe snapper	lutjanus kasmira	snapper
LVN	golden african snapper	lutjanus fulgens	snapper
LWE	blackspot snapper	lutjanus ehrenbergii	snapper
LWN	blueline snapper	lutjanus coeruleolineatus	snapper
LWP	pacific red snapper	lutjanus peru	snapper
LWQ	five-lined snapper	lutjanus quinquelineatus	snapper
LWT	timor snapper	lutjanus timorensis	snapper
LYW	slender sole	lyopsetta exilis	sole_flatfish
LZH	abu mullet	liza abu	mullet
LZI	greenback mullet	planiliza subviridis	mullet
LZJ	humphead snapper	lutjanus sanguineus	snapper
LZK	klunzinger's mullet	liza klunzingeri	mullet
LZS	leaping mullet	liza saliens	mullet
LZV	squaretail mullet	liza vaigiensis	mullet
MAA	blue mackerel	scomber australasicus	mackerel
MAC	atlantic mackerel	scomber scombrus	mackerel
MAD	yellow-eye mullet	aldrichetta forsteri	mullet
MAE	spotted eagle ray	aetobatus narinari	rays skates
MAG	smooth mactra	mactra glabrata	clam
MAL	malabar blood snapper	lutjanus malabaricus	snapper
MAN	mantas, devil rays nei	mobulidae	rays skates
MAS	pacific chub mackerel	scomber japonicus	mackerel
MAT	mactra surf clams nei	mactridae	clam
MAW	west african spanish mackerel	scomberomorus tritor	mackerel
MAX	mackerels nei	scombridae	mackerel
MAZ	scomber mackerels nei	scomber spp	mackerel
MCA	golden perch	macquaria ambigua	perch nspf

MDI	flatwhiskered catfish	pinirampus pirinampu	catfish
MEC	new zealand lobster	metanephrops challengeri	lobster
MEG	megrim	lepidorhombus whiffiagonis	other_flatfish
MFX	cannonball jellyfish	stomolophus meleagris	jellyfish
MGA	golden grey mullet	chelon auratus	mullet
MGC	thinlip grey mullet	chelon ramada	mullet
MGI	parassi mullet	mugil incilis	mullet
MGU	white mullet	mugil curema	mullet
MHH	whiskered sole	monochirus hispidus	sole_flatfish
MIP	dover sole	microstomus pacificus	sole_flatfish
MKG	thickback sole	microchirus variegatus	sole_flatfish
MLB	goldsilk seabream	acanthopagrus berda	seabream
MLM	blackhead seabream	acanthopagrus schlegeli	seabream
MLR	thicklip grey mullet	chelon labrosus	mullet
MMM	antarctic armless flounder	mancopsetta maculata	flounder_flatfish
MNZ	monkfishes nei	lophius spp	monkfish
MOD	horse mussels nei	modiolus spp	mussels
MON	angler(=monk)	lophius piscatorius	monkfish
MPC	electric catfish	malapterurus electricus	catfish
MPO	bull ray	aetomylaeus bovinus	rays skates
MPS	largemouth black bass	micropterus salmoides	bass
MQL	knobby swimcrab	macropipus tuberculatus	swimming_crab
MQO	large trough shell	mactra murchisoni	clam
MRB	cownose ray	rhinoptera bonasus	rays skates
MRG	panama hake	merluccius angustimanus	hake_groundfish
MRK	foureyed sole	microchirus ocellatus	sole_flatfish
MRM	lusitanian cownose ray	rhinoptera marginata	rays skates
MRR	ticon cownose ray	rhinoptera brasiliensis	rays skates
MRS	pacific cownose ray	rhinoptera steindachneri	rays skates
MSD	mackerel scad	decapterus macarellus	mackerel
MSX	sea mussels nei	mytilidae	mussels
MTV	globose clam	mactra veneriformis	clam
MUA	bobo mullet	joturus pichardi	mullet
MUB	lebranche mullet	mugil liza	mullet
MUC	mud carp	cirrhinus molitorella	carp
MUD	indo-pacific swamp crab	scylla serrata	other_crab
MUF	flathead grey mullet	mugil cephalus	mullet
MUL	mullets nei	mugilidae	mullet
MUN	taquilla clams	mulinia spp	clam
MUO	leaping african mullet	mugil capurrii	mullet
MUR	surmullet	mullus surmuletus	mullet
MVA	shortspine african angler	lophius vaillanti	monkfish
MVJ	blackfin goosefish	lophius gastrophysus	monkfish

MVO	devil anglerfish	lophius vomerinus	monkfish
MWF	metanephrops lobsters nei	metanephrops spp	lobster
MWY	fire eel	mastacembelus erythrotaenia	eels
MXI	channel-clinging crab	maguimithrax spinosissimus	other_crab
MXT	harbour spidercrab	mithrax armatus	other_crab
MYL	common eagle ray	myliobatis aquila	rays skates
MYN	asian redtail catfish	hemibagrus nemurus	catfish
MYV	mytilus mussels nei	mytilus spp	mussels
MYZ	so-iuy mullet	mugil soiuy	mullet
NAC	wolf-eel	anarrhichthys ocellatus	eels
NBZ	nimble spray crab	percnon gibbesi	other_crab
NCL	short neck clams nei	paphia spp	clam
NDN	grand ark	anadara grandis	clam
NDQ	antique ark	anadara antiquata	clam
NDZ	blood ark	anadara ovalis	clam
NEA	andaman lobster	metanephrops andamanicus	lobster
NEM	mozambique lobster	metanephrops mozambicus	lobster
NEP	norway lobster	nephrops norvegicus	lobster
NHA	north pacific hake	merluccius productus	hake_groundfish
NIP	nile perch	lates niloticus	nile perch
NKR	norwegian krill	meganyctiphanes norvegica	krill
NLG	spotted spiny lobster	panulirus guttatus	lobster
NLI	banded spiny lobster	panulirus marginatus	lobster
NPA	californian anchovy	engraulis mordax	anchovy
NPH	japanese spanish mackerel	scomberomorus niphonius	mackerel
NQT	black ark	anadara tuberculosa	clam
NRQ	brown ark	anadara similis	clam
NSD	spiny-back eel	notacanthus sexspinis	eels
NUG	green spiny lobster	panulirus gracilis	lobster
NUJ	japanese spiny lobster	panulirus japonicus	lobster
NUP	pronghorn spiny lobster	panulirus penicillatus	lobster
NUQ	anomuran decapods nei	anomura	shrimp
NUR	ornate spiny lobster	panulirus ornatus	lobster
NUV	painted spiny lobster	panulirus versicolor	lobster
NYD	curlfin sole	pleuronichthys decurrens	sole_flatfish
NYR	three-spot flounder	ancylopsetta dendritica	flounder_flatfish
NYV	hornyhead turbot	pleuronichthys verticalis	turbot_flatfish
OAL	senegalese sole	solea senegalensis	sole_flatfish
OAM	adriatic sole	pegusa impar	sole_flatfish
OAO	ovate sole	solea ovata	sole_flatfish
OCC	common octopus	octopus vulgaris	octopus
OCH	chilean oyster	ostrea chilensis	oysters
OCL	black brotula	cherublemma emmelas	cusk_groundfish

OCM	horned and musky octopuses	eledone spp	octopus
OCN	white-spotted octopus	callistoctopus macropus	octopus
OCT	octopuses, etc. nei	octopodidae	octopus
OCZ	octopuses nei	octopus spp	octopus
ODL	boxlip mullet	oedalechilus labeo	mullet
ODV	periscope crab	podophthalmus vigil	other_crab
OEA	blue tilapia	oreochromis aureus	tilapia
OEB	blackthroat seaperch	doederleinia berycoides	perch nspf
OFC	webbed flying squid	ommastrephes caroli	squid
OFE	orangeback flying squid	sthenoteuthis pteropus	squid
OFJ	neon flying squid	ommastrephes bartramii	squid
OGZ	gasar cupped oyster	crassostrea tulipa	oysters
OHO	kariba tilapia	oreochromis mortimeri	tilapia
OHT	pacific eyed flounder	bothus constellatus	flounder_flatfish
OIH	tilapia shiranus	oreochromis shiranus	tilapia
OIJ	greater hooked squid	moroteuthopsis ingens	squid
OIT		oreochromis tanganicae	tilapia
OJD	indian squid	loligo duvauceli	squid
OJE	swordtip squid	loligo edulis	squid
OKB	butter catfish	ompok bimaculatus	catfish
OMM	flying squids nei	ommastrephes spp	squid
OMZ	ommastrephidae squids nei	ommastrephidae	squid
ONA	golden trout	oncorhynchus aguabonita	trout
OOH	punctuated snake-eel	ophichthus remiger	eels
OPH	cusk-eels, brotulas nei	ophidiidae	cusk_groundfish
OPI	longfin snake-eel	pisodonophis cancrivorus	eels
OPM	middling thread herring	opisthonema medirastre	herring
OPP	pacific ocean perch	sebastes alutus	ocean perch_groundfish
OPU	slender thread herring	opisthonema bulleri	herring
OQJ	pygmy octopus	octopus joubini	octopus
OQM	red sea mantis shrimp	erugosquilla massavensis	shrimp
OQT	spider octopus	octopus salutii	octopus
OQY	mexican four-eyed octopus	octopus maya	octopus
ORC	pacific salmons nei	oncorhynchus spp	other_salmon
ORM	longfin tilapia	oreochromis macrochir	tilapia
ORY	orange roughy	hoplostethus atlanticus	orange roughy
OSG	pudgy cuskeel	spectrunculus grandis	cusk_groundfish
OST	flat and cupped oysters nei	ostreidae	oysters
OTQ	caribbean reef octopus	octopus briareus	octopus
OUB	wide-eyed flounder	bothus podas	flounder_flatfish

OUK	african squid	alloteuthis africana	squid
OUL	european common squid	alloteuthis subulata	squid
OUM	midsize squid	alloteuthis media	squid
OUN	leopard flounder	bothus pantherinus	flounder_flatfish
OUQ	pacific thorny oyster	spondylus princeps	oysters
OUW	alloteuthis squids nei	alloteuthis spp	squid
OWX	snake eels nei	ophichthidae	eels
OXP	butterfish, greenbone	odax pullus	butterfish
OXW	blue-nile tilapia, hybrid	oreochromis aureus x o. niloticus	tilapia
OYA	american cupped oyster	crassostrea virginica	oysters
OYC	cupped oysters nei	crassostrea spp	oysters
OYF	european flat oyster	ostrea edulis	oysters
OYG	pacific cupped oyster	magallana gigas	oysters
OYH	olympia oyster	ostrea conchaphila	oysters
OYI	indian backwater oyster	crassostrea madrasensis	oysters
OYM	mangrove cupped oyster	crassostrea rhizophorae	oysters
OYR	cortez oyster	crassostrea corteziensis	oysters
OYS	sydney cupped oyster	saccostrea glomerata	oysters
OYX	flat oysters nei	ostrea spp	oysters
PAC	common pandora	pagellus erythrinus	seabream
PAG	softshell red crab	paralomis granulosa	other_crab
PAI		paralomis spp	other_crab
PAM	mississippi paddlefish	polyodon spathula	sturgeon
PAR	red pandora	pagellus bellottii	seabream
PAX	pandoras nei	pagellus spp	seabream
PCR	tanner crabs nei	chionoecetes spp	snow_crab
PCX	pike-congers nei	muraenesox spp	pike
PCY	round whitefish	prosopium cylindraceum	whitefish
PEH	redtail catfish	phractocephalus hemioliopterus	catfish
PEW	white perch	morone americana	bass
PEX	freshwater perches nei	lates spp	perch nspf
PGA	panga seabream	pterogymnus laniarius	seabream
PGP	pangas catfish	pangasius pangasius	catfish
PGS	striped catfish	pangasianodon hypophthalmus	catfish
PGZ	pangas catfishes nei	pangasius spp	catfish
PHA	south pacific hake	merluccius gayi	hake_groundfish
PII	striped eel catfish	plotosus lineatus	eels
PIL	european pilchard(=sardine)	sardina pilchardus	sardine
PIN	pink(=humpback) salmon	oncorhynchus gorbuscha	pink_salmon
PJH	chilean jagged lobster	projasus bahamondei	lobster
PJM	pacific jack mackerel	trachurus symmetricus	horse mackerel_jack
PJR	brazilian sandperch	pinguipes brasilianus	perch nspf

PKP	trout sweetlips	plectorhinchus pictus	trout
PLA	amer. plaice(=long rough dab)	hippoglossoides platessoides	other_flatfish
PLE	european plaice	pleuronectes platessa	plaice_flatfish
PLN	european whitefish	coregonus lavaretus	whitefish
PLZ	righteye flounders nei	pleuronectidae	flounder_flatfish
PNM	blacklip pearl oyster	pinctada margaritifera	oysters
PNX	silverlip pearl oyster	pinctada maxima	oysters
POI	offshore rockfish	pontinus kuhlii	ocean perch_groundfish
POK	saithe(=pollock)	pollachius virens	pollock_groundfish
POL	pollack	pollachius pollachius	pollock_groundfish
POS	southern blue whiting	micromesistius australis	blue whiting_groundfish
PPO	pacific pompano	peprilus simillimus	butterfish
PQG	carrot squat lobster	pleuroncodes monodon	lobster
PQQ	equal-sized surf clam	spisula aequilatera	clam
PQZ		pangasius djambal	catfish
PRD	antarctic octopuses	pareledone spp	octopus
PSB	spiny turbot	psettodes bennettii	turbot_flatfish
PSG	glacial squid	psychroteuthis glacialis	squid
PSL	pink spiny lobster	palinurus mauritanicus	lobster
PSM	pond smelt	hypomesus olidus	smelts
PTE	penguin wing oyster	pteria penguin	oysters
PTS	pacific littleneck clam	protothaca staminea	clam
PUN	gray eel-catfish	plotosus canius	eels
PVQ	senegalese smooth swimcrab	sanquerus validus	swimming_crab
PWG	waigieu seaperch	psammoperca waigiensis	perch nspf
PWQ	starry flounder	platichthys stellatus	flounder_flatfish
PYS	raphael catfish	platydoras costatus	catfish
PZZ	southern rock bass	paralabrax callaensis	bass
QFP	ohrid trout	salmo letnica	trout
QLE	rough mantis shrimp	squilla empusa	shrimp
QPH	henslow�s swimming crab	polybius henslowii	swimming_crab
QPT	tehuelche scallop	aequipecten tehuelchus	scallops
QQX	giant clams nei	tridacna spp	clam
QSC	queen scallop	aequipecten opercularis	scallops
QSX	todarodes flying squids nei	todarodes spp	squid
QYP	amazon sailfin catfish	pterygoplichthys pardalis	catfish
QYR	greenspotted rockfish	sebastes chlorostictus	ocean perch_groundfish
QYS	gopher rockfish	sebastes carnatus	ocean perch_groundfish

QYT	cowcod	sebastes levis	ocean perch_groundfish
QYV	bank rockfish	sebastes rufus	ocean perch_groundfish
RAB	short mackerel	rastrelliger brachysoma	mackerel
RAE	european razor clam	solen marginatus	clam
RAF	island mackerel	rastrelliger faughni	mackerel
RAG	indian mackerel	rastrelliger kanagurta	mackerel
RAJ	rays and skates nei	rajidae	rays skates
RAL	slender rainbow sardine	dussumieria elopsoides	sardine
RAP	pacific razor clam	siliqua patula	clam
RAQ	spanner crab	ranina ranina	other_crab
RAS	rainbow sardine	dussumieria acuta	sardine
RAX	indian mackerels nei	rastrelliger spp	mackerel
RCW	red swamp crawfish	procambarus clarkii	crawfish
REA	redbanded seabream	pagrus auriga	seabream
REB	beaked redfish	sebastes mentella	ocean perch_groundfish
REC	cape redfish	sebastes capensis	ocean perch_groundfish
RED	atlantic redfishes nei	sebastes spp	ocean perch_groundfish
REG	golden redfish	sebastes norvegicus	ocean perch_groundfish
REL	king of herrings	regalecus glesne	herring
REN	acadian redfish	sebastes fasciatus	ocean perch_groundfish
REQ	patagonian redfish	sebastes oculatus	ocean perch_groundfish
RES	mangrove red snapper	lutjanus argentimaculatus	snapper
REV	red seabream	pagrus major	seabream
RFA	whiteleg skate	amblyraja taaf	rays skates
RFC	darkblotched rockfish	sebastes crameri	ocean perch_groundfish
RFE	english sole	pleuronectes vetulus	sole_flatfish
RGI	smooth butterfly ray	gymnura micrura	rays skates
RGL	spiny butterfly ray	gymnura altavela	rays skates
RGM	california butterfly ray	gymnura marmorata	rays skates
RIE	fine flounder	paralichthys adspersus	flounder_flatfish
RIQ	brown rockfish	sebastes auriculatus	ocean perch_groundfish
RJA	white skate	rostroraja alba	rays skates
RJB	blue skate	dipturus batis	rays skates
RJC	thornback ray	raja clavata	rays skates
RJD	little skate	leucoraja erinacea	rays skates

RJE	small-eyed ray	raja microocellata	rays skates
RJF	shagreen ray	leucoraja fullonica	rays skates
RJG	arctic skate	amblyraja hyperborea	rays skates
RJH	blonde ray	raja brachyura	rays skates
RJI	sandy ray	leucoraja circularis	rays skates
RJK	sailray	rajella lintea	rays skates
RJL	barndoor skate	dipturus laevis	rays skates
RJM	spotted ray	raja montagui	rays skates
RJN	cuckoo ray	leucoraja naevus	rays skates
RJO	longnosed skate	dipturus oxyrinchus	rays skates
RJP	soft skate	malacoraja spinacidermis	rays skates
RJQ	spinetail ray	bathyraja spinicauda	rays skates
RJR	starry ray	amblyraja radiata	rays skates
RJS	smooth skate	malacoraja senta	rays skates
RJT	winter skate	leucoraja ocellata	rays skates
RJU	undulate ray	raja undulata	rays skates
RJY	round ray	rajella fyllae	rays skates
RKQ	noah's ark	arca noae	clam
RMG	black rockfish	sebastes melanops	ocean perch_groundfish
RMQ	south american catfish	rhamdia quelen	catfish
RMV	manta rays	mobula spp	rays skates
RNM	small scale mud carp	cirrhinus microlepis	carp
ROA	stout bobtail squid	rossia macrosoma	squid
ROC	pacific rock crab	cancer productus	other_crab
ROJ	pacific sandperch	prolatilus jugularis	perch nspf
ROS	rock sole	lepidopsetta bilineata	sole_flatfish
ROY	white bass	morone chrysops	bass
RPG	red porgy	pagrus pagrus	seabream
RPJ	redstripe rockfish	sebastes proriger	ocean perch_groundfish
RPN	sea snails	rapana spp	snail
RPW	veined rapa whelk	rapana venosa	snail
RQZ	turkey wing	arca zebra	clam
RRH	red-eye round herring	etrumeus teres	herring
RRN	sculptured mitten lobster	parribacus antarcticus	lobster
RRV	yelloweye rockfish	sebastes ruberrimus	ocean perch_groundfish
RRW	rio skate	rioraja agassizi	rays skates
RSC	rough scad	trachurus lathami	horse mackerel_jack
RSE	red scorpionfish	scorpaena scrofa	scorpionfish
RSS	goldlined seabream	rhabdosargus sarba	seabream
RTE	round ribbontail ray	taeniurops meyeni	rays skates

RTZ	graery threadfin seabass	cratinus agassizii	sea bass
RVB	redbanded rockfish	sebastes babcocki	ocean perch_groundfish
RVM	blackgill rockfish	sebastes melanostomus	ocean perch_groundfish
RVN	china rockfish	sebastes nebulosus	ocean perch_groundfish
RVR	aurora rockfish	sebastes aurora	ocean perch_groundfish
RVT	stripetail rockfish	sebastes saxicola	ocean perch_groundfish
RVZ	sharpchin rockfish	sebastes zacentrus	ocean perch_groundfish
RXR	hawk-wing conch	strombus raninus	conch
RXS	haffara seabream	rhabdosargus haffara	seabream
RYH	banded eagle ray	aetomylaeus niehofii	rays skates
SAA	round sardinella	sardinella aurita	sardine
SAB	sablefish	anoplopoma fimbria	sablefish
SAE	madeiran sardinella	sardinella maderensis	sardine
SAG	goldstripe sardinella	sardinella gibbosa	sardine
SAH	blackchin tilapia	sarotherodon melanotheron	tilapia
SAL	atlantic salmon	salmo salar	atlantic_salmon
SAM	bali sardinella	sardinella lemuru	sardine
SAN	sandeels(=sandlances) nei	ammodytes spp	eels
SAR	mango tilapia	sarotherodon galilaeus	tilapia
SAS	scaled sardines	harengula spp	sardine
SAV	volga pikeperch	sander volgensis	sauger
SBA	axillary seabream	pagellus acarne	seabream
SBC	bocaccio rockfish	sebastes paucispinis	ocean perch_groundfish
SBG	gilthead seabream	sparus aurata	seabream
SBH	striped bass, hybrid	morone chrysops x m. saxatilis	bass
SBM	duckbill catfish	sorubim lima	catfish
SBP	pargo breams nei	pagrus spp	seabream
SBR	blackspot seabream	pagellus bogaraveo	seabream
SBS	saddled seabream	oblada melanura	seabream
SBX	porgies, seabreams nei	sparidae	seabream
SBY	silvergray rockfish	sebastes brevispinis	ocean perch_groundfish
SBZ	zebra seabream	diplodus cervinus	seabream
SCA	american sea scallop	placopecten magellanicus	scallops
SCB	atlantic bay scallop	argopecten irradians	scallops
SCC	calico scallop	argopecten gibbus	scallops
SCD	blue swimming crab	portunus pelagicus	swimming_crab

SCE	great atlantic scallop	pecten maximus	scallops
SCF	turbots nei	scophthalmidae	turbot_flatfish
SCG	weathervane scallop	patinopecten caurinus	scallops
SCH	pacific calico scallop	argopecten ventricosus	scallops
SCO	scorpionfishes, redfishes nei	scorpaenidae	scorpionfish
SCQ	peruvian calico scallop	argopecten purpuratus	scallops
SCR	spinous spider crab	maja squinado	other_crab
SCS	scorpionfishes, rockfishes nei	scorpaena spp	scorpionfish
SCX	scallops nei	pectinidae	scallops
SCY	lesser slipper lobster	scyllarus arctus	lobster
SCZ	new zealand scallop	pecten novaezelandiae	scallops
SDC	basketwork eel	diastobranchus capensis	eels
SDT	chili sea catfish	notarius troschelii	catfish
SDW	brown sea catfish	sciades dowii	catfish
SED	olive rockfish	sebastes serranoides	ocean perch_groundfish
SEQ	rosy rockfish	sebastes rosaceus	ocean perch_groundfish
SFD	splitnose rockfish	sebastes diploproa	ocean perch_groundfish
SFE	greenstriped rockfish	sebastes elongatus	ocean perch_groundfish
SFI	sevan trout	salmo ischchan	trout
SFJ	copper rockfish	sebastes caurinus	ocean perch_groundfish
SFK	shortbelly rockfish	sebastes jordani	ocean perch_groundfish
SFL	korean rockfish	sebastes schlegeli	ocean perch_groundfish
SFO	starry rockfish	sebastes constellatus	ocean perch_groundfish
SFP	treefish	sebastes serriceps	ocean perch_groundfish
SFT	rougheye rockfish	sebastes aleutianus	ocean perch_groundfish
SFV	norway redfish	sebastes viviparus	ocean perch_groundfish
SFW	vermilion rockfish	sebastes miniatus	ocean perch_groundfish
SFY	blue rockfish	sebastes mystinus	ocean perch_groundfish
SGO	chilipepper rockfish	sebastes goodei	ocean perch_groundfish
SHA	american shad	alosa sapidissima	shad

SHC	pontic shad	alosa immaculata	shad
SHD	allis and twaite shads	alosa alosa, a. fallax	shad
SHG	american gizzard shad	dorosoma cepedianum	shad
SHH	hickory shad	alosa mediocris	shad
SHR	sharpsnout seabream	diplodus puntazzo	seabream
SHZ	shads nei	alosa spp	shad
SIB	muddy arrowtooth eel	ilyophis brunneus	eels
SIE	pacific sierra	scomberomorus sierra	mackerel
SIL	silversides(=sand smelts) nei	atherinidae	smelts
SIQ	player scorpionfish	scorpaena histrio	scorpionfish
SIX	sardinellas nei	sardinella spp	sardine
SJA	great mediterranean scallop	pecten jacobaeus	scallops
SKA	raja rays nei	raja spp	rays skates
SKH	various sharks nei	selachimorpha (pleurotremata)	shark
SKX	sharks, rays, skates, etc. nei	elasmobranchii	shark
SLC	caribbean spiny lobster	panulirus argus	lobster
SLD	santer seabream	cheimerius nufar	seabream
SLF	carpenter seabream	argyrozona argyrozona	seabream
SLL	slinger seabream	chrysoblephus puniceus	seabream
SLN	natal spiny lobster	palinurus delagoae	lobster
SLO	common spiny lobster	palinurus elephas	lobster
SLS	southern spiny lobster	palinurus gilchristi	lobster
SLT	slender tuna	allothunnus fallai	other_tuna
SLV	tropical spiny lobsters nei	panulirus spp	lobster
SLX	salmonoids nei	salmonoidei	other_salmon
SLZ	salmonids nei	salmonidae	other_salmon
SMC	smoothmouth sea catfish	arius heudelotii	catfish
SME	european smelt	osmerus eperlanus	smelts
SMR	rainbow smelt	osmerus mordax	smelts
SMX	smelts nei	osmerus spp, hypomesus spp	smelts
SNA	snappers nei	lutjanus spp	snapper
SNC	southern red snapper	lutjanus purpureus	snapper
SNL	lane snapper	lutjanus synagris	snapper
SNQ	small red scorpionfish	scorpaena notata	scorpionfish
SNU	russell's snapper	lutjanus russelli	snapper
SOA	southeast atlantic soles nei	austroglossus spp	sole_flatfish
SOC	sockeye(=red) salmon	oncorhynchus nerka	sockeye_salmon
SOE	mud sole	austroglossus pectoralis	sole_flatfish
SOI	razor clams, knife clams nei	solenidae	clam
SOL	common sole	solea solea	sole_flatfish

SOM	wels(=som) catfish	silurus glanis	catfish
SOS	sand sole	pegusa lascaris	sole_flatfish
SOT	spottail spiny turbot	psettodes belcheri	turbot_flatfish
SOW	west coast sole	austroglossus microlepis	sole_flatfish
SOX	soles nei	soleidae	sole_flatfish
SOY	so-iny (redlip) mullet	liza haematocheilus	mullet
SPG	canary rockfish	sebastes pinniger	ocean perch_groundfish
SPH	sheepshead	archosargus probatocephalus	seabream
SPU	spotted seabass	dicentrarchus punctatus	sea bass
SQA	argentine shortfin squid	illex argentinus	squid
SQC	common squids nei	loligo spp	squid
SQE	european flying squid	todarodes sagittatus	squid
SQF	veined squid	loligo forbesii	squid
SQG	angolan flying squid	todarodes angolensis	squid
SQI	northern shortfin squid	illex illecebrosus	squid
SQJ	japanese flying squid	todarodes pacificus	squid
SQL	longfin squid	dorytheutis (amerigo) pealeii	squid
SQM	broadtail shortfin squid	illex coindetii	squid
SQO	opalescent inshore squid	loligo opalescens	squid
SQP	patagonian squid	dorytheutis (amerigo) gahi	squid
SQR	european squid	loligo vulgaris	squid
SQS	sevenstar flying squid	martialia hyadesi	squid
SQU	various squids nei	loliginidae, ommastrephidae	squid
SQZ	inshore squids nei	loliginidae	squid
SRG	sargo breams nei	diplodus spp	seabream
SRH	silver-stripe round herring	spratelloides gracilis	herring
SRO	amur catfish	silurus asotus	catfish
SRR	antarctic starry skate	amblyraja georgiana	rays skates
SSC	southern australia scallop	pecten fumatus	scallops
SSD	surf clams nei	spisula spp	clam
SSK	kaup's arrowtooth eel	synaphobranchus kaupii	eels
SSM	atlantic spanish mackerel	scomberomorus maculatus	mackerel
SSW	angola rockfish	scorpaena angolensis	ocean perch_groundfish
STA	three spotted tilapia	oreochromis andersonii	tilapia
STB	striped bass	morone saxatilis	bass
STC	black stone crab	menippe mercenaria	other_crab
STL	buccaneer anchovy	encrasicholina punctifer	anchovy
STO	stolephorus anchovies nei	stolephorus spp	anchovy
STS	streaked seerfish	scomberomorus lineolatus	mackerel
STT	stingrays, butterfly rays nei	dasyatidae	rays skates
STU	sturgeons nei	acipenseridae	sturgeon

STV	walleye	sander vitreus	pickerel
SUS	surf smelt	hypomesus pretiosus	smelts
SVC	silver carp	hypophthalmichthys molitrix	carp
SVF	brook trout	salvelinus fontinalis	trout
SVV	spotted-fin rockfish	scorpaena stephanica	ocean perch_groundfish
SVX	stomatopods nei	stomatopoda	shrimp
SWA	white seabream	diplodus sargus	seabream
SWD	yellowmouth rockfish	sebastes reedi	ocean perch_groundfish
SWJ	red sea seabream	diplodus noct	seabream
SWM	swimming crabs, etc. nei	portunidae	swimming_crab
SXR	marbled swamp eel	synbranchus marmoratus	eels
SZC	sauger	sander canadensis	sauger
SZH	sobaity seabream	sparidentex hasta	seabream
TCL	taca clam	leukoma thaca	clam
TDD	smooth giant clam	tridacna derasa	clam
TDG	giant clam	tridacna gigas	clam
TDQ	lesser flying squid	todaropsis eblanae	squid
TDS	fluted giant clam	tridacna squamosa	clam
TEJ	giant seabass	stereolepis gigas	sea bass
TFP	antarctic flying squid	todarodes filippovae	squid
THA	atlantic thread herring	opisthonema oglinum	herring
THE	tigerperches nei	terapontidae	perch nspf
THI	congo sea catfish	cathorops fuerthii	catfish
THP	pacific thread herring	opisthonema libertate	herring
THQ	flathead lobster	thenus orientalis	lobster
THS	thickback soles nei	microchirus spp	sole_flatfish
THX	thread herrings nei	opisthonema spp	herring
TLL	sabaki tilapia	oreochromis spilurus	tilapia
TLM	mozambique tilapia	oreochromis mossambicus	tilapia
TLN	nile tilapia	oreochromis niloticus	tilapia
TLP	tilapias nei	oreochromis spp	tilapia
TLR	redbreast tilapia	coptodon rendalli	tilapia
TLZ	redbelly tilapia	tilapia zillii	tilapia
TMB	southwest atlantic butterfish	stromateus brasiliensis	butterfish
TOA	antarctic toothfish	dissostichus mawsoni	toothfish
TOD	electric rays nei	torpedinidae	rays skates
TOE	torpedo rays	torpedo spp	rays skates
TOL	toli shad	tenualosa toli	shad
TOP	patagonian toothfish	dissostichus eleginoides	toothfish
TOX	tonguefishes	cynoglossidae	other_flatfish

TQB	southern lobsterette	thymops birsteini	lobster
TQU	pacific horse clam	tresus nuttallii	clam
TQZ	pacific horse clams nei	tresus spp	clam
TRO	trouts nei	salmo spp	trout
TRR	rainbow trout	oncorhynchus mykiss	trout
TRS	sea trout	salmo trutta	trout
TSD	twaite shad	alosa fallax	shad
TSQ	wellington flying squid	nototodarus sloanii	squid
TTO	electric ray	tetronarce nobiliana	rays skates
TTR	marbled electric ray	torpedo marmorata	rays skates
TUR	turbot	scophthalmus maximus	turbot_flatfish
TUX	tuna-like fishes nei	scombroidei	other_tuna
TWN	longtail shad	tenualosa macrura	shad
TWS	madamango sea catfish	cathorops spixii	catfish
TXE	telescope snail	telescopium telescopium	snail
TZY	spiny scorpionfish	trachyscorpia echinata	scorpionfish
UCD	mangrove ghost crab	ucides occidentalis	other_crab
UCG	west african fiddler crab	uca tangeri	other_crab
UCH	chilean sea urchin	loxechinus albus	sea urchin
UDQ	swarming squat lobster	munida gregaria	lobster
UDV	club scallop	pseudamussium clavatum	scallops
UGU	unihorn octopus	scaeurgus unicirrhus	octopus
UHA	largetooth flounder	pseudorhombus arsius	flounder_flatfish
UHB	robust clubhook squid	onykia robusta	squid
UHG	malayan flounder	pseudorhombus malayanus	flounder_flatfish
UHJ	javan flounder	pseudorhombus javanicus	flounder_flatfish
UHL	bigfin reef squid	sepioteuthis lessoniana	squid
UHX	hooked squids nei	onykia spp	squid
UKB	black sea urchin	arbacia lixula	sea urchin
UKQ	purple pacific sea urchin	echinometra vanbrunti	sea urchin
ULO	solid surf clam	spisula solida	clam
ULT	subtruncate surf clam	spisula subtruncata	clam
UMP	asian moon scallop	amusium pleuronectes	scallops
UMZ	mazatlan sole	achirus mazatlanus	sole_flatfish
UOP	blue mud shrimp	upogebia pugettensis	shrimp
UPE	peruvian flounder	etropus peruvianus	flounder_flatfish
UPR	argentinian sandperch	pseudopercis semifasciata	perch nspf
URC	sea urchins nei	strongylocentrotus spp	sea urchin
URM	stony sea urchin	paracentrotus lividus	sea urchin
URQ	rugose squat lobster	munida rugosa	lobster
URS	european edible sea urchin	echinus esculentus	sea urchin
URW	arabian whip lobster	puerulus sewelli	lobster
URX	sea urchins, etc. nei	echinoidea	sea urchin

USK	tusk(=cusk)	brosme brosme	cusk_groundfish
UTV	network sole	achirus scutum	sole_flatfish
UZX	stumpnoses nei	rhabdosargus spp	seabream
VAS	tete sea catfish	ariopsis seemanni	catfish
VCC	fringelip mullet	crenimugil crenilabis	mullet
VEP	pacific anchoveta	cetengraulis mysticetus	anchovy
VET	anchoveta(=peruvian anchovy)	engraulis ringens	anchovy
VLO	spiny lobsters nei	palinuridae	lobster
VMA	atlantic chub mackerel	scomber colias	mackerel
VMH	bluespot mullet	valamugil seheli	mullet
VSC	variegated scallop	mimachlamys varia	scallops
WBM	goatsbeard brotula	brotula multibarbata	cusk_groundfish
WHB	blue whiting(=poutassou)	micromesistius poutassou	blue whiting_groundfish
WHF	whitefishes nei	coregonus spp	whitefish
WHG	whiting	merlangius merlangus	whiting_groundfish
WHL	lake(=common) whitefish	coregonus clupeaformis	whitefish
WHS	sillago-whitings	sillaginidae	whiting_groundfish
WIO	cyclope sole	pegusa triophthalma	sole_flatfish
WIT	witch flounder	glyptocephalus cynoglossus	flounder_flatfish
WIX	heterobranchus catfish nei	heterobranchus spp	catfish
WIY	ordway's brotula	brotula ordwayi	cusk_groundfish
WLL	oriental sole	brachirus orientalis	sole_flatfish
WPM	pacific harvestfish	peprilus medius	butterfish
WRO	widow rockfish	sebastes entomelas	ocean perch_groundfish
WRR	whitehead's round herring	etrumeus whiteheadi	herring
WSN	white stumpnose	rhabdosargus globiceps	seabream
WZA	cadenat's sole	pegusa cadenati	sole_flatfish
XAX	eels, morays, congers nei	anguilliformes	eels
XBX		paralabrax spp	sea bass
XHC	hetero-clarias catfish, hybrid	h. longifilis x c. gariepinus	catfish
XMB	psammobatis sand skates nei	psammobatis spp	rays skates
XPX	frogfishes nei	antennariidae	frog
XSX	scorpionfishes, gurnards nei	scorpaeniformes	scorpionfish
XYL	fantail flounder	xystreurys liolepis	flounder_flatfish
YAR	green mud crab	scylla paramamosain	other_crab
YAT	purple mud crab	scylla tranquebarica	other_crab
YBX	charybdis crabs nei	charybdis spp	other_crab
YCH	yellow catfish	pelteobagrus fulvidraco	catfish

YEL	yellowtail flounder	limanda ferruginea	flounder_flatfish
YES	yellowfin sole	limanda aspera	sole_flatfish
YEZ	great sandeel	hyperoplus lanceolatus	eels
YFL	yellow striped flounder	pseudopleuronectes herzenst.	flounder_flatfish
YJN	star-studded grouper	hyporthodus niphobles	grouper
YLA	spanish slipper lobster	scyllarides aequinoctialis	lobster
YLL	mediterranean slipper lobster	scyllarides latus	lobster
YLO	ridged slipper lobster	scyllarides nodifer	lobster
YLP	god's flounder	cyclopsetta panamensis	flounder_flatfish
YLQ	toothed flounder	cyclopsetta querna	flounder_flatfish
YLU	blunt slipper lobster	scyllarides squammosus	lobster
YLW	orange mud crab	scylla olivacea	other_crab
YMW	deep water sole	bathysolea profundicola	sole_flatfish
YNN	silver seatrout	cynoscion nothus	trout
YNU	portuguese sole	dagetichthys lusitanicus	sole_flatfish
YNY	guinean sole	synaptura cadenati	sole_flatfish
YQP	salema butterfish	peprilus snyderi	butterfish
YRO	yellowtail rockfish	sebastes flavidus	ocean perch_groundfish
YSB	gulf flounder	paralichthys albigutta	flounder_flatfish
YSE	pacific sand sole	psettichthys melanostictus	sole_flatfish
YSF	california flounder	paralichthys californicus	flounder_flatfish
YSH	southern flounder	paralichthys lethostigma	flounder_flatfish
YSO	fourspot flounder	paralichthys oblongus	flounder_flatfish
YSW	speckled flounder	paralichthys woolmani	flounder_flatfish
YTT	schoolmaster gonate squid	berryteuthis magister	squid
YUR	diamondback squid	thysanoteuthis rhombus	squid
YWF	yellowfin seabream	acanthopagrus latus	seabream
ZGC	mediterranean sand eel	gymnammodytes cicerelus	eels
ZGS	smooth sandeel	gymnammodytes semisquamatus	eels
ZHB	east asian bullfrog	hoplobatrachus rugulosus	frog
ZPS	speckled scorpionfish	pontinus sierra	scorpionfish
ZUZ	gilded catfish	zungaro zungaro	catfish
ZYE	delicate scallop	zygochlamys delicatula	scallops
ZYP	patagonian scallop	zygochlamys patagonica	scallops
HJV	pink abalone	haliotis corrugata	abalone
HAZ	black abalone	haliotis cracherodii	abalone
HRW	southern green abalone	haliotis fulgens	abalone
ABF	red abalone	haliotis rufescens	abalone
HSW	white abalone	haliotis sorenseni	abalone
HTW	threaded abalone	haliotis assimilis	abalone
LIY	donkey's ear abalone	haliotis asinina	abalone

ABJ	japanese abalone	haliotis discus	abalone
ABG	giant abalone	haliotis gigantea	abalone
LGX	glistening abalone	haliotis glabra	abalone
ABP	perlemoen abalone	haliotis midae	abalone
LGW	oval abalone	haliotis ovina	abalone
ABR	blacklip abalone	haliotis rubra	abalone
HLT	tuberculate abalone	haliotis tuberculata	abalone
ABM	small abalone	haliotis diversicolor	abalone
LQQ	planate abalone	haliotis planata	abalone
LQW	variable abalone	haliotis varia	abalone
AIZ	pinto abalone	haliotis kamtschatkana	abalone
AJF		haliotis spadicea	abalone
DZS	rainbow abalone	haliotis iris	abalone
UQK	greenlip abalone	haliotis laevigata	abalone
UQL	roe's abalone	haliotis roei	abalone
UQM		haliotis scalaris	abalone
UQN		haliotis cyclobates	abalone
ABX	abalones nei	haliotis spp	abalone
PCO	pacific cod	gadus macrocephalus	cod_groundfish
COD	atlantic cod	gadus morhua	cod_groundfish
CDZ	northern cods nei	gadus spp	cod_groundfish
CRB	blue crab	callinectes sapidus	blue_crab
CAL	callinectes swimcrabs nei	callinectes spp	blue_crab
KCD	red king crab	paralithodes camtschaticus	king_crab
KCS	king crabs	paralithodes spp	king_crab
DOL	common dolphinfish	coryphaena hippurus	dolphin
EHG	redmouth grouper	aethaloperca rogaa	grouper
AYG	slender grouper	anyperodon leucogrammicus	grouper
UFT	goldribbon soapfish	aulacocephalus temmincki	grouper
RNL	pink maomao	caprodon longimanus	grouper
CFF	peacock hind	cephalopholis argus	grouper
CFZ	golden hind	cephalopholis aurantia	grouper
CVK	chocolate hind	cephalopholis boenak	grouper
CFL	graysby	cephalopholis cruentata	grouper
CFY	bluespotted hind	cephalopholis cyanostigma	grouper
CFJ	coney	cephalopholis fulva	grouper
CFH	yellowfin hind	cephalopholis hemistiktos	grouper
CWI	garish hind	cephalopholis igarashiensis	grouper
UKR	leopard hind	cephalopholis leopardus	grouper
CFM	freckled hind	cephalopholis microprion	grouper
CFI	coral hind	cephalopholis miniata	grouper
CFQ	niger hind	cephalopholis nigri	grouper
CZU	duskyfin hind	cephalopholis nigripinnis	grouper

CFX	sixblotch hind	cephalopholis sexmaculata	grouper
EFT	tomato hind	cephalopholis sonnerati	grouper
CWR	strawberry hind	cephalopholis spiloparaea	grouper
IIX		cephalopholis spp	grouper
EFA	bluespotted seabass	cephalopholis taeniops	grouper
CWU	darkfin hind	cephalopholis urodeta	grouper
MPV	humpback grouper	cromileptes altivelis	grouper
DED	leather bass	dermatolepis dermatolepis	grouper
DEV	smooth grouper	dermatolepis striolata	grouper
DLY	bighead sand perch	diplectrum euryplectrum	grouper
PES	sand perch	diplectrum formosum	grouper
DLK	highfin sand perch	diplectrum labarum	grouper
DLM	mexican sand perch	diplectrum macropoma	grouper
DLX	torpedo sand perch	diplectrum maximum	grouper
DLA	inshore sand perch	diplectrum pacificum	grouper
DLD	pond perch	diplectrum radiale	grouper
EFU		epinephelides armatus	grouper
EFC	rooster hind	epinephelus acanthistius	grouper
EFD	rock hind	epinephelus adscensionis	grouper
GPW	white grouper	epinephelus aeneus	grouper
EPA	hong kong grouper	epinephelus akaara	grouper
EFB	white-edged grouper	epinephelus albomarginatus	grouper
EFY	banded grouper	epinephelus amblycephalus	grouper
GPS	spotted grouper	epinephelus analogus	grouper
EFN	catface grouper	epinephelus andersoni	grouper
EPR	areolate grouper	epinephelus areolatus	grouper
EFW	yellow grouper	epinephelus awoara	grouper
EFK	duskytail grouper	epinephelus bleekeri	grouper
EFE	longtooth grouper	epinephelus bruneus	grouper
EWC	whitespotted grouper	epinephelus caeruleopunctatus	grouper
EFJ	dogtooth grouper	epinephelus caninus	grouper
EWZ	moustache grouper	epinephelus chabaudi	grouper
EFH	brownspotted grouper	epinephelus chlorostigma	grouper
EPF	olive grouper	epinephelus cifuentesi	grouper
ENI	orange-spotted grouper	epinephelus coioides	grouper
EPK	goldblotch grouper	epinephelus costae	grouper
EPY	speckled blue grouper	epinephelus cyanopodus	grouper
ESE	saddletail grouper	epinephelus daemelii	grouper
ELD	spinycheek grouper	epinephelus diacanthus	grouper
EED	speckled hind	epinephelus drummondhayi	grouper
EWE	dotted grouper	epinephelus epistictus	grouper
EEF	rock grouper	epinephelus fasciatomaculosus	grouper
EEA	blacktip grouper	epinephelus fasciatus	grouper

EWV	barred-chest grouper	epinephelus faveatus	grouper
EEV	blue-and-yellow grouper	epinephelus flavocaeruleus	grouper
EEL	yellowedge grouper	epinephelus flavolimbatus	grouper
EWF	brown-marbled grouper	epinephelus fuscoguttatus	grouper
EEG	dungat grouper	epinephelus goreensis	grouper
EEU	red hind	epinephelus guttatus	grouper
EEI	haifa grouper	epinephelus haifensis	grouper
EEE	bridled grouper	epinephelus heniochus	grouper
EEX	starspotted grouper	epinephelus hexagonatus	grouper
EWI	marquesan grouper	epinephelus irroratus	grouper
EET	atlantic goliath grouper	epinephelus itajara	grouper
EEB	starry grouper	epinephelus labriformis	grouper
EEN	giant grouper	epinephelus lanceolatus	grouper
EES	striped grouper	epinephelus latifasciatus	grouper
EWW	longspine grouper	epinephelus longispinis	grouper
EEM	snubnose grouper	epinephelus macrospilos	grouper
EEC	highfin grouper	epinephelus maculatus	grouper
EEJ	speckled grouper	epinephelus magniscuttis	grouper
MAR	malabar grouper	epinephelus malabaricus	grouper
GPD	dusky grouper	epinephelus marginatus	grouper
EZP	one-blotch grouper	epinephelus melanostigma	grouper
EER	honeycomb grouper	epinephelus merra	grouper
EWM	netfin grouper	epinephelus miliaris	grouper
GPR	red grouper	epinephelus morio	grouper
EEP	comet grouper	epinephelus morrhua	grouper
EWU	white-blotched grouper	epinephelus multinotatus	grouper
EEY	misty grouper	epinephelus mystacinus	grouper
ELG	warsaw grouper	epinephelus nigritus	grouper
EZN	star-studded grouper	epinephelus niphobles	grouper
EFV	snowy grouper	epinephelus niveatus	grouper
EWO	eightbar grouper	epinephelus octofasciatus	grouper
EWP	dot-dash grouper	epinephelus poecilonotus	grouper
EWY	smallscaled grouper	epinephelus polylepis	grouper
EEK	camouflage grouper	epinephelus polyphekadion	grouper
EWG	striped-fin grouper	epinephelus posteli	grouper
EEQ	hawaiian grouper	epinephelus quernus	grouper
EZQ	pacific goliath grouper	epinephelus quinquefasciatus	grouper
EFQ	longfin grouper	epinephelus quoyanus	grouper
EZR	oblique-banded grouper	epinephelus radiatus	grouper
EWR	red-tipped grouper	epinephelus retouti	grouper
EPV	halfmoon grouper	epinephelus rivulatus	grouper
EIF	convict grouper	epinephelus septemfasciatus	grouper
EFX	sixbar grouper	epinephelus sexfasciatus	grouper

EZO	foursaddle grouper	epinephelus spilotoceps	grouper
GPX	groupers nei	epinephelus spp	grouper
EIT	black-dotted grouper	epinephelus stictus	grouper
EPZ	epaulet grouper	epinephelus stoliczkae	grouper
GPN	nassau grouper	epinephelus striatus	grouper
EWS	summan grouper	epinephelus summana	grouper
EPT	greasy grouper	epinephelus tauvina	grouper
EIR	threespot grouper	epinephelus trimaculatus	grouper
EWT	reticulate grouper	epinephelus tuamotuensis	grouper
EWL	potato grouper	epinephelus tukula	grouper
EIU	wavy-lined grouper	epinephelus undulosus	grouper
GCA	masked grouper	gracila albomarginata	grouper
YHP	sevenbar grouper	hyporthodus ergastularius	grouper
MTI	comb grouper	mycteroperca acutirostris	grouper
MAB	black grouper	mycteroperca bonaci	grouper
MKC	venezuelan grouper	mycteroperca cidi	grouper
MKF	island grouper	mycteroperca fusca	grouper
MKN	yellowmouth grouper	mycteroperca interstitialis	grouper
MKJ	gulf grouper	mycteroperca jordani	grouper
MKM	gag	mycteroperca microlepis	grouper
MKH	scamp	mycteroperca phenax	grouper
MKR	leopard grouper	mycteroperca rosacea	grouper
MKU	mottled grouper	mycteroperca rubra	grouper
GPB	brazilian groupers nei	mycteroperca spp	grouper
MKT	tiger grouper	mycteroperca tigris	grouper
MKV	yellowfin grouper	mycteroperca venenosa	grouper
GBS	broomtail grouper	mycteroperca xenarcha	grouper
EME	squaretail coralgrouper	plectropomus areolatus	grouper
EML	blacksaddled coralgrouper	plectropomus laevis	grouper
EMO	leopard coralgrouper	plectropomus leopardus	grouper
PLM	spotted coralgrouper	plectropomus maculatus	grouper
EMW	highfin coralgrouper	plectropomus oligacanthus	grouper
EMU	roving coralgrouper	plectropomus pessuliferus	grouper
EMN	marbled coralgrouper	plectropomus punctatus	grouper
IWX	coralgroupers nei	plectropomus spp	grouper
OOW	golden grouper	saloptia powelli	grouper
TSR	oval grouper	triso dermopterus	grouper
VRA	white-edged lyretail	variola albimarginata	grouper
VRL	yellow-edged lyretail	variola louti	grouper
SNR	northern red snapper	lutjanus campechanus	snapper
SNX	snappers nei	lutjanidae	snapper
YYC		actinopyga agassizii	sea cucumber
KUE	deep-water redfish	actinopyga echinites	sea cucumber

YVV		actinopyga lecanora	sea cucumber
KUY	surf redfish	actinopyga mauritiana	sea cucumber
KUQ	hairy blackfish	actinopyga miliaris	sea cucumber
YGP	panning's blackfish	actinopyga palauensis	sea cucumber
YGS	new caledonia blackfish	actinopyga spinea	sea cucumber
CUJ	japanese sea cucumber	apostichopus japonicus	sea cucumber
TKV	warty sea cucumber	apostichopus parvimensis	sea cucumber
JPN	furry sea cucumber	astichopus multifidus	sea cucumber
KZA		athyonidium chilensis	sea cucumber
JPO	brown mottled sea cucumber	australostichopus mollis	sea cucumber
KUW	leopard fish	bohadschia argus	sea cucumber
JLZ		bohadschia atra	sea cucumber
KUH	chalky cucumber	bohadschia marmorata	sea cucumber
BDX	brownspotted sandfish	bohadschia similis	sea cucumber
JTQ		bohadschia subrubra	sea cucumber
BDV	brown sandfish	bohadschia vitiensis	sea cucumber
KHG		cucumaria frondosa	sea cucumber
KHE		cucumaria japonica	sea cucumber
KGH		cucumaria kerguelensis	sea cucumber
HZT		heterocucumis godeffroyi	sea cucumber
JBN		holothuria arenacava	sea cucumber
JCE		holothuria arenicola	sea cucumber
JHG		holothuria arguinensis	sea cucumber
HFA	lollyfish	holothuria atra	sea cucumber
JCH		holothuria cinerascens	sea cucumber
HHW	snakefish sea cucumber	holothuria coluber	sea cucumber
HFE	pinkfish	holothuria edulis	sea cucumber
JCI	red snakefish	holothuria flavomaculata	sea cucumber
JCJ		holothuria fuscocinerea	sea cucumber
HFF	white teatfish	holothuria fuscogilva	sea cucumber
HOZ	elephant trunkfish	holothuria fuscopunctata	sea cucumber
JCK		holothuria hilla	sea cucumber
HFI	bottleneck sea cucumber	holothuria impatiens	sea cucumber
JCL		holothuria kefersteini	sea cucumber
JCO		holothuria lessoni	sea cucumber
HFQ	white threads fish	holothuria leucospilota	sea cucumber
JCQ		holothuria mexicana	sea cucumber
HFN	black teatfish	holothuria nobilis	sea cucumber
JCW		holothuria notabilis	sea cucumber
JDB		holothuria pardalis	sea cucumber
JDD		holothuria pervicax	sea cucumber
HFC	sandfish	holothuria scabra	sea cucumber

JDE		holothuria spinifera	sea cucumber
WBX		holothuria spp	sea cucumber
HFT	cotton spinner	holothuria tubulosa	sea cucumber
JDG		holothuria whitmaei	sea cucumber
CUX	sea cucumbers nei	holothuroidea	sea cucumber
HIZ	four-sided sea cucumber	isostichopus badionotus	sea cucumber
JCF	giant sea cucumber	isostichopus fuscus	sea cucumber
MZH		molpadia musculus	sea cucumber
QHO		paradota marionensis	sea cucumber
TKG	giant red sea cucumber	parastichopus californicus	sea cucumber
TVK		parastichopus tremulus	sea cucumber
EHV	blackspotted sea cucumber	pearsonothuria graeffei	sea cucumber
JGQ		pseudocnus laevigatus	sea cucumber
QEY		pseudostichopus peripatus	sea cucumber
QAB		psolidium poriferum	sea cucumber
QAG		psolus ephippifer	sea cucumber
QAK		psolus paradubiosus	sea cucumber
RXO		staurocucumis liouvillei	sea cucumber
JCC	greenfish	stichopus chloronotus	sea cucumber
JNG		stichopus herrmanni	sea cucumber
KUN	selenka's sea cucumber	stichopus horrens	sea cucumber
JPQ		stichopus monotuberculatus	sea cucumber
JPR		stichopus naso	sea cucumber
JPT	ocellated sea cucumber	stichopus ocellatus	sea cucumber
JPU		stichopus pseudohorrens	sea cucumber
JCR	royal cucumber	stichopus regalis	sea cucumber
JCV	curryfish	stichopus variegatus	sea cucumber
JPW		stichopus vastus	sea cucumber
TFQ	prickly redfish	thelenota ananas	sea cucumber
HLX	amber fish	thelenota anax	sea cucumber
JDZ		thelenota rubralineata	sea cucumber
TXQ		trachythyone muricata	sea cucumber
ACN	hooktooth dogfish	aculeola nigra	shark
PTH	pelagic thresher	alopias pelagicus	shark
THR	thresher sharks nei	alopias spp	shark
BTH	bigeye thresher	alopias superciliosus	shark
ALV	thresher	alopias vulpinus	shark
CSA		apristurus acanutus	shark
CSU	atlantic ghost catshark	apristurus atlanticus	shark
CSN	brown catshark	apristurus brunneus	shark
CSQ	hoary catshark	apristurus canutus	shark
CSG		apristurus gibbosus	shark

CSW	longfin catshark	apristurus herklotsi	shark
APD	smallbelly catshark	apristurus indicus	shark
APV	broadnose catshark	apristurus investigatoris	shark
CSJ	japanese catshark	apristurus japonicus	shark
CSZ	longnose catshark	apristurus kampae	shark
APQ	iceland catshark	apristurus laurussonii	shark
CSF	longhead catshark	apristurus longicephalus	shark
CSX	flathead catshark	apristurus macrorhynchus	shark
APM		apristurus macrostomus	shark
APF	madeira catshark	apristurus maderensis	shark
APA	ghost catshark	apristurus manis	shark
APX	smalleye catshark	apristurus microps	shark
APY		apristurus micropterygeus	shark
APW	largenose catshark	apristurus nasutus	shark
APK	smallfin catshark	apristurus parvipinnis	shark
APZ	spatulasnout catshark	apristurus platyrhynchus	shark
APP	deep-water catshark	apristurus profundorum	shark
CSV	broadgill catshark	apristurus riveri	shark
APC	saldanha catshark	apristurus saldanha	shark
APJ	pale catshark	apristurus sibogae	shark
ASI	south china catshark	apristurus sinensis	shark
ASO	spongehead catshark	apristurus spongiceps	shark
API	deep-water catsharks	apristurus spp	shark
ASE	panama ghost catshark	apristurus stenseni	shark
ASW	borneo catshark	apristurus verweyi	shark
ASY	australian spotted catshark	asymbolus analis	shark
AXM		asymbolus spp	shark
ASV	gulf catshark	asymbolus vincenti	shark
ATM	australian marbled catshark	atelomycterus macleayi	shark
ATY	coral catshark	atelomycterus marmoratus	shark
AUK	kanakorum catshark	aulohalaelurus kanakorum	shark
AUL	australian blackspot catshark	aulohalaelurus labiosus	shark
OBW	blind shark	brachaelurus waddi	shark
RSK	requiem sharks nei	carcharhinidae	shark
CVX	ground sharks	carcharhiniformes	shark
CCN	blacknose shark	carcharhinus acronotus	shark
ALS	silvertip shark	carcharhinus albimarginatus	shark
CCA	bignose shark	carcharhinus altimus	shark
CCY	graceful shark	carcharhinus amblyrhynchoides	shark
AML	grey reef shark	carcharhinus amblyrhynchos	shark
CCF	pigeye shark	carcharhinus amboinensis	shark

CCX	borneo shark	carcharhinus borneensis	shark
BRO	copper shark	carcharhinus brachyurus	shark
CCB	spinner shark	carcharhinus brevipinna	shark
CCC	nervous shark	carcharhinus cautus	shark
CCD	whitecheek shark	carcharhinus dussumieri	shark
FAL	silky shark	carcharhinus falciformis	shark
CCZ	creek whaler	carcharhinus fitzroyensis	shark
CCG	galapagos shark	carcharhinus galapagensis	shark
CCK	pondicherry shark	carcharhinus hemiodon	shark
CCO	finetooth shark	carcharhinus isodon	shark
CCJ		carcharhinus leiodon	shark
CCE	bull shark	carcharhinus leucas	shark
CCL	blacktip shark	carcharhinus limbatus	shark
OCS	oceanic whitetip shark	carcharhinus longimanus	shark
CCM	hardnose shark	carcharhinus macloti	shark
BLR	blacktip reef shark	carcharhinus melanopterus	shark
DUS	dusky shark	carcharhinus obscurus	shark
CCV	caribbean reef shark	carcharhinus perezi	shark
CCP	sandbar shark	carcharhinus plumbeus	shark
CCR	smalltail shark	carcharhinus porosus	shark
CCI	blackspot shark	carcharhinus sealei	shark
CCS	night shark	carcharhinus signatus	shark
CCQ	spottail shark	carcharhinus sorrah	shark
CWZ	carcharhinus sharks nei	carcharhinus spp	shark
CCU	australian blacktip shark	carcharhinus tilstoni	shark
CCW	blacktail reef shark	carcharhinus wheeleri	shark
CCT	sand tiger shark	carcharias taurus	shark
LOU	indian sand tiger	carcharias tricuspidatus	shark
WSH	great white shark	carcharodon carcharias	shark
CEA	needle dogfish	centrophorus acus	shark
GVA	blackfin gulper shark	centrophorus atromarginatus	shark
GUP	gulper shark	centrophorus granulosus	shark
CEU	dumb gulper shark	centrophorus harrissoni	shark
GVI	black gulper shark	centrophorus isodon	shark
CPL	lowfin gulper shark	centrophorus lusitanicus	shark
CEM	smallfin gulper shark	centrophorus moluccensis	shark
CEK	taiwan gulper shark	centrophorus niaukang	shark
CWO	gulper sharks nei	centrophorus spp	shark
GUQ	leafscale gulper shark	centrophorus squamosus	shark
CEE	mosaic gulper shark	centrophorus tessellatus	shark
CPU	little gulper shark	centrophorus uyato	shark
CFB	black dogfish	centroscyllium fabricii	shark
CYG	granular dogfish	centroscyllium granulatum	shark

CYK	bareskin dogfish	centroscyllium kamoharai	shark
CYN	combtooth dogfish	centroscyllium nigrum	shark
CYT	ornate dogfish	centroscyllium ornatum	shark
CYR	whitefin dogfish	centroscyllium ritteri	shark
YCX	centroscyllium dogfishes nei	centroscyllium spp	shark
CYO	portuguese dogfish	centroscymnus coelolepis	shark
CYP	longnose velvet dogfish	centroscymnus crepidater	shark
CYY	shortnose velvet dogfish	centroscymnus cryptacanthus	shark
CYW	roughskin dogfish	centroscymnus owstoni	shark
CYU	plunket shark	centroscymnus plunketi	shark
CZI		centroscymnus spp	shark
CPF	reticulated swellshark	cephaloscyllium fasciatum	shark
CPS	draughtsboard shark	cephaloscyllium isabellum	shark
CPT	australian swellshark	cephaloscyllium laticeps	shark
CPN	whitefinned swellshark	cephaloscyllium nascione	shark
CPA	indian swellshark	cephaloscyllium silasi	shark
CPH	balloon shark	cephaloscyllium sufflans	shark
CPB	blotchy swell shark	cephaloscyllium umbratile	shark
CPV	swellshark	cephaloscyllium ventriosum	shark
CPC	lollipop catshark	cephalurus cephalus	shark
BSK	basking shark	cetorhinus maximus	shark
HCM	hooktooth shark	chaenogaleus macrostoma	shark
ORA	arabian carpetshark	chiloscyllium arabicum	shark
KYL	burmese bambooshark	chiloscyllium burmensis	shark
ORP	bluespotted bambooshark	chiloscyllium caerulopunctatum	shark
ORR	grey bambooshark	chiloscyllium griseum	shark
YYL	hasselt's bambooshark	chiloscyllium hasselti	shark
ORI	slender bambooshark	chiloscyllium indicum	shark
ORH	whitespotted bambooshark	chiloscyllium plagiosum	shark
ORB	brownbanded bambooshark	chiloscyllium punctatum	shark
OQX	bamboosharks nei	chiloscyllium spp	shark
HWR	african frilled shark	chlamydoselachus africana	shark
HXC	frilled shark	chlamydoselachus anguineus	shark
CHZ	roughskin spurdog	cirrhigaleus asper	shark
CHF	mandarin dogfish	cirrhigaleus barbifer	shark
OPC	barbelthroat carpetshark	cirrhoscyllium expolitum	shark
OPF	taiwan saddled carpetshark	cirrhoscyllium formosanum	shark
OPJ	saddle carpetshark	cirrhoscyllium japonicum	shark
CPE	harlequin catshark	ctenacis fehlmanni	shark
SCK	kitefin shark	dalatias licha	shark

DCA	birdbeak dogfish	deania calcea	shark
SDH	rough longnose dogfish	deania hystricosa	shark
SDU	arrowhead dogfish	deania profundorum	shark
SDQ	longsnout dogfish	deania quadrispinosa	shark
DNA	deania dogfishes nei	deania spp	shark
SHB	bramble shark	echinorhinus brucus	shark
ECK	prickly shark	echinorhinus cookei	shark
PEB	cuban ribbontail catshark	eridacnis barbouri	shark
PEA	pygmy ribbontail catshark	eridacnis radcliffei	shark
PED	african ribbontail catshark	eridacnis sinuans	shark
ETB	blurred smooth lantern shark	etmopterus bigelowi	shark
ETH	shorttail lanternshark	etmopterus brachyurus	shark
ETU	lined lanternshark	etmopterus bullisi	shark
ETE		etmopterus compagnoi	shark
ETO	combtoothed lanternshark	etmopterus decacuspidatus	shark
ETI	broadbanded lanternshark	etmopterus gracilispinis	shark
ETM	southern lanternshark(lucifer)	etmopterus granulosus	shark
ETN	carribean lanternshark	etmopterus hillianus	shark
ETF	blackbelly lanternshark	etmopterus lucifer	shark
ETL	mollers lantern shark	etmopterus molleri	shark
ETT	african lanternshark	etmopterus polli	shark
ETR	great lanternshark	etmopterus princeps	shark
ETP	smooth lanternshark	etmopterus pusillus	shark
ETZ	fringfin lanternshark	etmopterus schultzi	shark
ETQ	thorny lanternshark	etmopterus sentosus	shark
ETX	velvet belly	etmopterus spinax	shark
ETK	splendid lanternshark	etmopterus splendidus	shark
SHL	lanternsharks nei	etmopterus spp	shark
ETJ	brown lanternshark	etmopterus unicolor	shark
ETV	hawaiian lanternshark	etmopterus villosus	shark
ETW	green lanternshark	etmopterus virens	shark
ORE	tasselled wobbegong	eucrossorhinus dasypogon	shark
EUZ	taillight shark	euprotomicroides zantedeschia	shark
EUP	pygmy shark	euprotomicrus bispinatus	shark
EUB	winghead shark	eusphyra blochii	shark
TFM	whiskery shark	furgaleus macki	shark
TIG	tiger shark	galeocerdo cuvier	shark
GAG	tope shark	galeorhinus galeus	shark
GAA	roughtail catshark	galeus arae	shark
GHA	atlantic sawtail catshark	galeus atlanticus	shark
GAB	australian sawtail catshark	galeus boardmani	shark

GAE	gecko catshark	galeus eastmani	shark
GAO		galeus longirostris	shark
SHO	blackmouth catshark	galeus melastomus	shark
GAM	mouse catshark	galeus murinus	shark
GAN	broadfin sawtail catshark	galeus nipponensis	shark
GAP	peppered catshark	galeus piperatus	shark
GAQ	african sawtail catshark	galeus polli	shark
GAI	blacktip sawtail catshark	galeus sauteri	shark
GAH	dwarf sawtail catshark	galeus schultzi	shark
GAU	crest-tail catsharks nei	galeus spp	shark
ORX	short-tail nurse shark	ginglymostoma brevicaudatum	shark
GNC	nurse shark	ginglymostoma cirratum	shark
GNG	nurse sharks nei	ginglymostoma spp	shark
CGA	ganges shark	glyphis gangeticus	shark
CGG	speartooth shark	glyphis glyphis	shark
TGF	sailback houndshark	gogolia filewoodi	shark
CPG	slender smooth-hound	gollum attenuatus	shark
HAA	arabian catshark	halaelurus alcocki	shark
HAB	speckled catshark	halaelurus boesemani	shark
HAE	blackspotted catshark	halaelurus buergeri	shark
HAN	dusky catshark	halaelurus canescens	shark
HAO	new zealand catshark	halaelurus dawsoni	shark
HAH	bristly catshark	halaelurus hispidus	shark
HAV	spotless catshark	halaelurus immaculatus	shark
HAY	lined catshark	halaelurus lineatus	shark
HAG	mud catshark	halaelurus lutarius	shark
HAK	tiger catshark	halaelurus natalensis	shark
HAQ	quagga catshark	halaelurus quagga	shark
HPE	puffadder shyshark	haploblepharus edwardsii	shark
HPF	brown shyshark	haploblepharus fuscus	shark
HPP	dark shyshark	haploblepharus pictus	shark
HEH	sicklefin weasel shark	hemigaleus microstoma	shark
HEE	snaggletooth shark	hemipristis elongata	shark
ORF	indonesia speckled carpetshark	hemiscyllium freycineti	shark
ORK	papuan epaulette shark	hemiscyllium hallstromi	shark
ORN	epaulette shark	hemiscyllium ocellatum	shark
ORQ	hooded carpetshark	hemiscyllium strahani	shark
ORW	speckled carpetshark	hemiscyllium trispeculare	shark
THJ	japanese topeshark	hemitriakis japanica	shark
THL	whitefin topeshark	hemitriakis leucoperiptera	shark
HXT	sharpnose sevengill shark	heptranchias perlo	shark
HDQ	bullhead sharks	heterodontiformes	shark

HEF	horn shark	heterodontus francisci	shark
HEG	crested bullhead shark	heterodontus galeatus	shark
HEJ	japanese bullhead shark	heterodontus japonicus	shark
HEM	mexican hornshark	heterodontus mexicanus	shark
HEK	port jackson shark	heterodontus portusjacksoni	shark
HEQ	galapagos bullhead shark	heterodontus quoyi	shark
HEA	whitespotted bullhead shark	heterodontus ramalheira	shark
HEZ	zebra bullhead shark	heterodontus zebra	shark
OBH	bluegray carpetshark	heteroscyllium colcloughi	shark
HYY	longnose pygmy shark	heteroscymnoides marleyi	shark
HXW	frilled and cow sharks	hexanchiformes	shark
SBL	bluntnose sixgill shark	hexanchus griseus	shark
HXN	bigeyed sixgill shark	hexanchus nakamurai	shark
HOP	african spotted catshark	holohalaelurus punctatus	shark
HOR	izak catshark	holohalaelurus regani	shark
THH	blacktipe tope	hypogaleus hyugaensis	shark
TIK	longnose houndshark	iago garricki	shark
TIO	bigeye houndshark	iago omanensis	shark
ISB	cookie cutter shark	isistius brasiliensis	shark
ISP	largetooth cookiecutter shark	isistius plutodus	shark
CIO	daggernose shark	isogomphodon oxyrhynchus	shark
SMA	shortfin mako	isurus oxyrinchus	shark
LMA	longfin mako	isurus paucus	shark
MAK	mako sharks	isurus spp	shark
LMT	broadfin shark	lamiopsis temmincki	shark
LMD	salmon shark	lamna ditropis	shark
POR	porbeagle	lamna nasus	shark
MSK	mackerel sharks,porbeagles nei	lamnidae	shark
LMZ	mackerel sharks	lamniformes	shark
CLL	barbeled houndshark	leptocharias smithii	shark
CLD	sliteye shark	loxodon macrorhinus	shark
LMP	megamouth shark	megachasma pelagios	shark
LMO	goblin shark	mitsukurina owstoni	shark
CTU	gummy shark	mustelus antarcticus	shark
SDS	starry smooth-hound	mustelus asterias	shark
CTN	grey smooth-hound	mustelus californicus	shark
CTI	dusky smooth-hound	mustelus canis	shark
CTD	sharptooth smooth-hound	mustelus dorsalis	shark
CTF	striped smooth-hound	mustelus fasciatus	shark
CTE	spotless smooth-hound	mustelus griseus	shark

CTK	brown smooth-hound	mustelus henlei	shark
CTJ	smalleye smooth-hound	mustelus higmani	shark
MTL	spotted estuary smooth-hound	mustelus lenticulatus	shark
MUU	sicklefin smooth-hound	mustelus lunulatus	shark
MTZ	starspotted smooth-hound	mustelus manazo	shark
MTE	speckled smooth-hound	mustelus mento	shark
MTM	arabian smooth-hound	mustelus mosis	shark
SMD	smooth-hound	mustelus mustelus	shark
MTR	narrowfin smooth-hound	mustelus norrisi	shark
MUP	whitespotted smooth-hound	mustelus palumbes	shark
MPT	blackspotted smooth-hound	mustelus punctulatus	shark
SDP	narrownose smooth-hound	mustelus schmitti	shark
SDV	smooth-hounds nei	mustelus spp	shark
MUW	humpback smooth-hound	mustelus whitneyi	shark
CNX	whitenose shark	nasolamia velox	shark
ORZ	tawny nurse shark	nebrius ferrugineus	shark
NGA	sicklefin lemon shark	negaprion acutidens	shark
NGB	lemon shark	negaprion brevirostris	shark
NTC	broadnose sevengill shark	notorynchus cepedianus	shark
LOO	smalltooth sand tiger	odontaspis ferox	shark
ODH	bigeye sand tiger shark	odontaspis noronhai	shark
OCX	carpet sharks	orectolobiformes	shark
ORT	spotted wobbegong	orectolobus maculatus	shark
ORO	ornate wobbegong	orectolobus ornatus	shark
ORV	northern wobbegong	orectolobus wardi	shark
OXB	prickly dogfish	oxynotus bruniensis	shark
OXC	caribbean roughshark	oxynotus caribbaeus	shark
OXY	angular roughshark	oxynotus centrina	shark
OXZ	japanese roughshark	oxynotus japonicus	shark
OXN	sailfin roughshark	oxynotus paradoxus	shark
HEC	whitetip weasel shark	paragaleus leucolomatus	shark
HEI	atlantic weasel shark	paragaleus pectoralis	shark
IEI	slender weasel shark	paragaleus randalli	shark
HEN	straight-tooth weasel shark	paragaleus tengi	shark
OPY	collared carpetshark	parascyllium collare	shark
OPE	rusty carpetshark	parascyllium ferrugineum	shark
OPV	necklace carpetshark	parascyllium variolatum	shark
PAH	campeche catshark	parmaturus campechiensis	shark
PAE	mcmillan's cat shark	parmaturus macmillani	shark

PAV	blackgill catshark	parmaturus melanobranchius	shark
PAW	salamander shark	parmaturus pilosus	shark
PAY	filetail catshark	parmaturus xaniurus	shark
PEU	onefin catshark	pentanchus profundicolus	shark
PPW	sixgill sawshark	pliotrema warreni	shark
POU	striped catshark	poroderma africanum	shark
POE	barbeled catshark	poroderma marleyi	shark
POH	leopard catshark	poroderma pantherinum	shark
BSH	blue shark	prionace glauca	shark
PPC	longnose sawshark	pristiophorus cirratus	shark
PPJ	japanese sawshark	pristiophorus japonicus	shark
PPU	shortnose sawshark	pristiophorus nudipinnis	shark
PPH	bahamas sawshark	pristiophorus schroederi	shark
PWS	sawsharks nei	pristiophorus spp	shark
CPY	graceful catshark	proscyllium habereri	shark
PSK	crocodile shark	pseudocarcharias kamoharai	shark
PTM	false catshark	pseudotriakis microdon	shark
RHN	whale shark	rhincodon typus	shark
RHA	milk shark	rhizoprionodon acutus	shark
RHL	brazilian sharpnose shark	rhizoprionodon lalandii	shark
RHU	pacific sharpnose shark	rhizoprionodon longurio	shark
RHX	grey sharpnose shark	rhizoprionodon oligolinx	shark
RHR	caribbean sharpnose shark	rhizoprionodon porosus	shark
RHZ	sharpnose sharks nei	rhizoprionodon spp	shark
RHY	australian sharpnose shark	rhizoprionodon taylori	shark
RHT	atlantic sharpnose shark	rhizoprionodon terraenovae	shark
SHV	narrowmouthed catshark	schroederichthys bivius	shark
SHY	redspotted catshark	schroederichthys chilensis	shark
SHU	narrowtail catshark	schroederichthys maculatus	shark
SHN	slender catshark	schroederichthys tenuis	shark
SLA	spadenose shark	scoliodon laticaudus	shark
SYX	catsharks, etc. nei	scyliorhinidae	shark
SYB	polkadot catshark	scyliorhinus besnardi	shark
SYA	boa catshark	scyliorhinus boa	shark
SYC	small-spotted catshark	scyliorhinus canicula	shark
SYP	yellowspotted catshark	scyliorhinus capensis	shark
SYE	west african catshark	scyliorhinus cervigoni	shark
SYG	brownspotted catshark	scyliorhinus garmani	shark
SYH	freckled catshark	scyliorhinus haeckelii	shark
SYU	whitesaddled catshark	scyliorhinus hesperius	shark
SYM	blotched catshark	scyliorhinus meadi	shark
SYF	chain catshark	scyliorhinus retifer	shark
SCL	catsharks, nursehounds nei	scyliorhinus spp	shark

SYT	nursehound	scyliorhinus stellaris	shark
SYK		scyliorhinus tokubee	shark
SYZ	cloudy catshark	scyliorhinus torazame	shark
SYI	dwarf catshark	scyliorhinus torrei	shark
TSK	flapnose houndshark	scylliogaleus quecketti	shark
YSA	whitetail dogfish	scymnodalatias albicauda	shark
YSS	sherwood dogfish	scymnodalatias sherwoodi	shark
YSM	largespine velvet dogfish	scymnodon macracanthus	shark
SYO	smallmouth knifetooth dogfish	scymnodon obscurus	shark
SYR	knifetooth dogfish	scymnodon ringens	shark
QUX	scymnodon dogfishes nei	scymnodon spp	shark
SSQ	velvet dogfish	scymnodon squamulosus	shark
GSK	greenland shark	somniosus microcephalus	shark
SON	pacific sleeper shark	somniosus pacificus	shark
SOR	little sleeper shark	somniosus rostratus	shark
SSN	scalloped bonnethead	sphyrna corona	shark
SPV	whitefin hammerhead	sphyrna couardi	shark
SPL	scalloped hammerhead	sphyrna lewini	shark
SPE	scoophead	sphyrna media	shark
SPK	great hammerhead	sphyrna mokarran	shark
SPN	hammerhead sharks nei	sphyrna spp	shark
SPJ	bonnethead	sphyrna tiburo	shark
SPQ	smalleye hammerhead	sphyrna tudes	shark
SPZ	smooth hammerhead	sphyrna zygaena	shark
SPY	hammerhead sharks, etc. nei	sphyrnidae	shark
DGX	dogfish sharks nei	squalidae	shark
DGH	dogfishes and hounds nei	squalidae, scyliorhinidae	shark
SHX	dogfish sharks, etc. nei	squaliformes	shark
QUA	smalleye pygmy shark	squaliolus aliae	shark
QUL	spined pygmy shark	squaliolus laticaudus	shark
DGS	picked dogfish	squalus acanthias	shark
QUB	longnose spurdog	squalus blainville	shark
QUC	cuban dogfish	squalus cubensis	shark
QUJ	japanese spurdog	squalus japonicus	shark
DOP	shortnose spurdog	squalus megalops	shark
QUN	blacktailed spurdog	squalus melanurus	shark
QUK	shortspine spurdog	squalus mitsukurii	shark
QUR	cyrano spurdog	squalus rancureli	shark
DGZ	dogfishes nei	squalus spp	shark
QYW	spotted spiny dogfish	squalus suckleyi	shark
SUA	sawback angelshark	squatina aculeata	shark

SUF	african angelshark	squatina africana	shark
SUG	argentine angelshark	squatina argentina	shark
SUU	australian angelshark	squatina australis	shark
SUC	pacific angelshark	squatina californica	shark
SUD	sand devil	squatina dumeril	shark
SUO	taiwan angleshark	squatina formosa	shark
SWV	angular angel shark	squatina guggenheim	shark
SUJ	japanese angelshark	squatina japonica	shark
SUL	clouded angelshark	squatina nebulosa	shark
SZJ	hidden angel shark	squatina occulta	shark
SUT	smoothback angelshark	squatina oculata	shark
AGN	angelshark	squatina squatina	shark
SUE	ornate angelshark	squatina tergocellata	shark
SUN	ocellated angelshark	squatina tergocellatoides	shark
ASK	angelsharks, sand devils nei	squatinidae	shark
OSF	zebra shark	stegostoma fasciatum	shark
ORS	cobbler wobbegong	sutorectus tentaculatus	shark
TRB	whitetip reef shark	triaenodon obesus	shark
TRK	houndsharks, smoothhounds nei	triakidae	shark
TTA	sharpfin houndshark	triakis acutipinna	shark
TTM	spotted houndshark	triakis maculata	shark
TTE	sharptooth houndshark	triakis megalopterus	shark
TTY	banded houndshark	triakis scyllium	shark
LES	leopard shark	triakis semifasciata	shark
FYU	peaked shrimp	acanthephyra curtirostris	shrimp
FQQ		acanthephyra eximia	shrimp
FBX	subantarctic ruby prawn nei	acanthephyra spp	shrimp
EKC	aviu shrimp	acetes americanus	shrimp
EKA	australian paste shrimp	acetes australis	shrimp
ESH	northern mauxia shrimp	acetes chinensis	shrimp
EKR	tsivakihini paste shrimp	acetes erythraeus	shrimp
EKN	jawla paste shrimp	acetes indicus	shrimp
EKI	taiwan mauxia shrimp	acetes intermedius	shrimp
AKS	akiami paste shrimp	acetes japonicus	shrimp
EKE	southern mauxia shrimp	acetes serrulatus	shrimp
EKS	alamang shrimp	acetes sibogae	shrimp
ESV	jembret shrimp	acetes vulgaris	shrimp
FEI	flathead snapping shrimp	alpheus bisincisus	shrimp
FEB	teppo snapping shrimp	alpheus brevicristatus	shrimp
FED	forceps snapping shrimp	alpheus digitalis	shrimp

FEU	nymph snapping shrimp	alpheus euphrosyne	shrimp
FEL	red snapping shrimp	alpheus glaber	shrimp
FEG	daisy snapping shrimp	alpheus gracilipes	shrimp
FEE	bigclaw snapping shrimp	alpheus heterochaelis	shrimp
FEH	armed snapping shrimp	alpheus hoplocheles	shrimp
FEJ	japanese snapping shrimp	alpheus japonicus	shrimp
FEP	bristle snapping shrimp	alpheus spongiarum	shrimp
FET	queensland snapping shrimp	alpheus stephensoni	shrimp
FES	coral snapping shrimp	alpheus sublucanus	shrimp
GJL	kuro shrimp	argis lar	shrimp
ARS	giant red shrimp	aristaeomorpha foliacea	shrimp
AHW	indian red shrimp	aristaeomorpha woodmasoni	shrimp
ARI	aristeid shrimps nei	aristeidae	shrimp
AJA	arabian red shrimp	aristeus alcocki	shrimp
ARA	blue and red shrimp	aristeus antennatus	shrimp
ANJ	purplehead gamba prawn	aristeus antillensis	shrimp
AJN	smooth red shrimp	aristeus semidentatus	shrimp
AXR	aristeus shrimps nei	aristeus spp	shrimp
ARV	striped red shrimp	aristeus varidens	shrimp
AVD	stout red shrimp	aristeus virilis	shrimp
ASH	argentine stiletto shrimp	artemesia longinaris	shrimp
GBH	gabon shrimp	atya gabonensis	shrimp
YAJ	basket shrimp	atya innocous	shrimp
TYW	koros shrimp	atya pilipes	shrimp
YAC	camacuto shrimp	atya scabra	shrimp
TYV	soldier brush shrimp	atya spinipes	shrimp
YAU	ekusa shrimp	atya sulcatipes	shrimp
YSU	moluccas brush shrimp	atyopsis moluccensis	shrimp
YEO	orange shrimp	atypopenaeus formosus	shrimp
YET	periscope shrimp	atypopenaeus stenodactylus	shrimp
BGQ	northern blunt-tail shrimp	bentheogennema borealis	shrimp
BIQ	tanner's deep-water shrimp	benthesicymus tanneri	shrimp
MYQ	sabre prawn	campylonotus rathbunae	shrimp
RKI	african caridina	caridina africana	shrimp
CID	sawtooth caridina	caridina denticulata	shrimp
RKE	malagasy caridina	caridina edulis	shrimp
RKR	needlenose caridina	caridina gracilirostris	shrimp
RKV	smooth caridina	caridina laevis	shrimp
RKN	common caridina	caridina nilotica	shrimp
RKP	bengal caridina	caridina propinqua	shrimp
CZX	caridina shrimps nei	caridina spp	shrimp

RKK	tonkin caridina	caridina tonkinensis	shrimp
RKW	pugnose caridina	caridina weberi	shrimp
HLQ	green shrimp	chlorotocus crassicornis	shrimp
CGF	japanese sand shrimp	crangon affinis	shrimp
GQA	alaska shrimp	crangon alaskensis	shrimp
GQC	gray shrimp	crangon communis	shrimp
CSH	common shrimp	crangon crangon	shrimp
GQF	california shrimp	crangon franciscorum	shrimp
GQN	blacktailed shrimp	crangon nigricauda	shrimp
GQI	bay shrimp	crangon nigromaculata	shrimp
GQS	sand shrimp	crangon septemspinosa	shrimp
CNZ	crangon shrimps nei	crangon spp	shrimp
CRN	crangonid shrimps nei	crangonidae	shrimp
YFN	changallo shrimp	cryphiops caementarius	shrimp
YPJ	catherine shrimp	cryptopenaeus catherinae	shrimp
DKB	whip shrimp	dichelopandalus bonnieri	shrimp
UUL	yamato shrimp	eualus leptognathus	shrimp
UUM	greenland shrimp	eualus macilentus	shrimp
UUI	iso shrimp	eualus sinensis	shrimp
XHE	hunter shrimp	exhippolysmata ensirostris	shrimp
XHH	companion shrimp	exhippolysmata hastatoides	shrimp
XHO	cock shrimp	exhippolysmata oplophoroides	shrimp
XPA	cipango prawn	exopalaemon annandalei	shrimp
EXC	ridgetail prawn	exopalaemon carinicauda	shrimp
EXN	mamtom prawn	exopalaemon mani	shrimp
EXO	siberian prawn	exopalaemon modestus	shrimp
EXT	oriental prawn	exopalaemon orientalis	shrimp
EXY	roshna prawn	exopalaemon styliferus	shrimp
EXU	vietnamese crest prawn	exopalaemon vietnamicus	shrimp
GFT	armoured shrimp	glyphocrangon alata	shrimp
IER		glyphocrangon dentata	shrimp
GOQ	goblin prawn	glyphocrangon lowryi	shrimp
GFI	panama armoured shrimp	glyphocrangon sicaria	shrimp
GFS	spiny armoured shrimp	glyphocrangon spinulosa	shrimp
GFC	vicary armoured shrimp	glyphocrangon vicaria	shrimp
GFU	kangaroo shrimp	glyphus marsupialis	shrimp
HDS	trident shrimp	hadropenaeus lucasii	shrimp
HJD	chilean knife shrimp	haliporoides diomedeae	shrimp
JAQ	jack-knife shrimp	haliporoides sibogae	shrimp
KNI	knife shrimps nei	haliporoides spp	shrimp
KNS	knife shrimp	haliporoides triarthrus	shrimp
HTB	shortspine shrimp	heptacarpus brevirostris	shrimp
HFU	toy shrimp	heptacarpus futilirostris	shrimp

HUG	flexed shrimp	heptacarpus geniculatus	shrimp
HSQ	tsuno shrimp	heptacarpus pandaloides	shrimp
HKV	dorodotes shrimp	heterocarpoides levicarina	shrimp
HQF	three-spined nylon shrimp	heterocarpus affinis	shrimp
HKI	madagascar nylon shrimp	heterocarpus dorsalis	shrimp
HKF	armed nylon shrimp	heterocarpus ensifer	shrimp
HKJ	humpback nylon shrimp	heterocarpus gibbosus	shrimp
HVQ		heterocarpus grimaldii	shrimp
HKY	japanese nylon shrimp	heterocarpus hayashii	shrimp
HQO	panama nylon shrimp	heterocarpus hostilis	shrimp
HKT	smooth nylon shrimp	heterocarpus laevigatus	shrimp
HQV	short-spined nylon shrimp	heterocarpus parvispina	shrimp
CHS	chilean nylon shrimp	heterocarpus reedi	shrimp
HPQ	mino nylon shrimp	heterocarpus sibogae	shrimp
HZK	nylon shrimps nei	heterocarpus spp	shrimp
HTQ	scarred nylon shrimp	heterocarpus tricarinatus	shrimp
HUV	northern nylon shrimp	heterocarpus vicarius	shrimp
HPW	indian nylon shrimp	heterocarpus woodmasoni	shrimp
YMP	painted harlequin shrimp	hymenocera picta	shrimp
YMF	pacific ambereye	hymenodora frontalis	shrimp
HMQ	veiled shrimp	hymenopenaeus aequalis	shrimp
HUD	doris veiled shrimp	hymenopenaeus doris	shrimp
HZM	hymenopenaeus shrimps nei	hymenopenaeus spp	shrimp
LUQ	hoso shrimp	latreutes acicularis	shrimp
LYX	medusa shrimp	latreutes anoplonyx	shrimp
LSQ	platenose shrimp	latreutes laminirostris	shrimp
LTZ	flatnose shrimp	latreutes planirostris	shrimp
LNQ	indian small prawn	leandrites indicus	shrimp
UFU	ganges delta prawn	leptocarpus fluminicola	shrimp
UPO	bombay prawn	leptocarpus potamiscus	shrimp
LKG	lesser glass shrimp	leptochela gracilis	shrimp
NCK		lipkebe holthuisi	shrimp
LKH	wellington shrimp	lipkius holthuisi	shrimp
YSN	common cleaner shrimp	lysmata amboinensis	shrimp
YSK	lined shrimp	lysmata californica	shrimp
YSD	cardinal shrimp	lysmata debelius	shrimp
YMS	monaco shrimp	lysmata seticaudata	shrimp
YMV	indian lined shrimp	lysmata vittata	shrimp
HUK	cinnamon river prawn	macrobrachium acanthurus	shrimp
HJE	noumea river prawn	macrobrachium aemulum	shrimp
MBA	amazonian river prawn	macrobrachium amazonicum	shrimp
HJM	cauque river prawn	macrobrachium americanum	shrimp

HJT	koua river prawn	macrobrachium australe	shrimp
HJI	birma river prawn	macrobrachium birmanicum	shrimp
HJL	new caledonia river prawn	macrobrachium caledonicum	shrimp
MBK	painted river prawn	macrobrachium carcinus	shrimp
HJR	ganges river prawn	macrobrachium choprai	shrimp
HJY	kaira river prawn	macrobrachium dayanum	shrimp
HDX	congo river prawn	macrobrachium dux	shrimp
HQU	rough river prawn	macrobrachium equidens	shrimp
HJN	sweet river prawn	macrobrachium esculentum	shrimp
HFO	crane river prawn	macrobrachium formosense	shrimp
HJG	greybeard river prawn	macrobrachium geron	shrimp
HGJ	hawaii river prawn	macrobrachium grandimanus	shrimp
BKO	hancock's river prawn	macrobrachium hancocki	shrimp
HHQ	cascade river prawn	macrobrachium heterochirus	shrimp
HJJ	orana river prawn	macrobrachium idae	shrimp
HDK	slender river prawn	macrobrachium idella	shrimp
BKI	striped river prawn	macrobrachium intermedium	shrimp
BKA	jaro river prawn	macrobrachium jaroense	shrimp
BKV	java river prawn	macrobrachium javanicum	shrimp
BKK	agar river prawn	macrobrachium jelskii	shrimp
BKL	kuncho river prawn	macrobrachium lamarrei	shrimp
BKN	philippine river prawn	macrobrachium lanceifrons	shrimp
MKK	riceland prawn	macrobrachium lanchesteri	shrimp
BKR	monkey river prawn	macrobrachium lar	shrimp
BKT	scissor river prawn	macrobrachium latidactylus	shrimp
BKS	mountain river prawn	macrobrachium latimanus	shrimp
BKE	malayam scale prawn	macrobrachium lepidactyloides	shrimp
BKY	madagascar scale prawn	macrobrachium lepidactylus	shrimp
BKB	brackish river prawn	macrobrachium macrobrachion	shrimp
MBM	monsoon river prawn	macrobrachium malcolmsonii	shrimp
BKD	knobtooth prawn	macrobrachium mammilodactylus	shrimp
MMZ	shortleg river prawn	macrobrachium mirabile	shrimp
MNX	oriental river prawn	macrobrachium nipponense	shrimp
BIK	western river prawn	macrobrachium occidentale	shrimp
BKH	ohio river prawn	macrobrachium ohione	shrimp
BKF	buchura river prawn	macrobrachium olfersii	shrimp
BKQ	patsa river prawn	macrobrachium patsa	shrimp
BJP	muff prawn	macrobrachium pilimanus	shrimp
BJD	volta river prawn	macrobrachium raridens	shrimp
RQD	shortfinger river shrimp	macrobrachium rathbunae	shrimp
PRF	giant river prawn	macrobrachium rosenbergii	shrimp
BJE	hairy river prawn	macrobrachium rude	shrimp
BJM	goda river prawn	macrobrachium scabriculum	shrimp

BJG	sunda river prawn	macrobrachium sintangense	shrimp
PPF	river prawns nei	macrobrachium spp	shrimp
BJQ	suriname river prawn	macrobrachium surinamicum	shrimp
BJL	longarm river prawn	macrobrachium tenellum	shrimp
BJJ	forest river prawn	macrobrachium trompii	shrimp
BJY	dimua river prawn	macrobrachium villosimanus	shrimp
BJH	african river prawn	macrobrachium vollenhovenii	shrimp
MPF	swimming shrimp	macropetasma africana	shrimp
MPZ	salmon shrimp	mesopaeneus tropicalis	shrimp
MRX	nutshell shrimp	metacrangon procax	shrimp
MJV	tora velvet shrimp	metapenaeopsis acclivis	shrimp
MMD	rice velvet shrimp	metapenaeopsis andamanensis	shrimp
MJB	whiskered velvet shrimp	metapenaeopsis barbata	shrimp
MQB	beebe's velvet shrimp	metapenaeopsis beebei	shrimp
MJD	reef shrimp	metapenaeopsis borradailei	shrimp
MJI	scout velvet shrimp	metapenaeopsis crassissima	shrimp
MDJ	kishi velvet shrimp	metapenaeopsis dalei	shrimp
MJG	caribbean velvet shrimp	metapenaeopsis goodei	shrimp
MJJ	minstrel shrimp	metapenaeopsis hilarula	shrimp
MKO	insular velvet shrimp	metapenaeopsis kishinouyei	shrimp
MEQ	humpback shrimp	metapenaeopsis lamellata	shrimp
MLX	broad velvet shrimp	metapenaeopsis lata	shrimp
MMX	mining shrimp	metapenaeopsis mineri	shrimp
NMJ	mogi velvet shrimp	metapenaeopsis mogiensis	shrimp
NMU	northern velvet shrimp	metapenaeopsis novaeguineae	shrimp
NMN	southern velvet shrimp	metapenaeopsis palmensis	shrimp
NMF	philip velvet shrimp	metapenaeopsis philippii	shrimp
NMQ	pink velvet shrimp	metapenaeopsis rosea	shrimp
NMY	fiddler shrimp	metapenaeopsis stridulans	shrimp
MEX	tolo velvet shrimp	metapenaeopsis toloensis	shrimp
MWL	velvet shrimp	metapenaeopsis wellsi	shrimp
MTJ	jinga shrimp	metapenaeus affinis	shrimp
MEK	fire shrimp	metapenaeus alcocki	shrimp
MAQ	spiny greasyback shrimp	metapenaeus anchistus	shrimp
MBB	greentail shrimp	metapenaeus bennettae	shrimp
MPB	yellow shrimp	metapenaeus brevicornis	shrimp
MSQ	wood shrimp	metapenaeus conjunctus	shrimp
MTD	western school shrimp	metapenaeus dalli	shrimp
MSY	demons prawn	metapenaeus demani	shrimp
MPD	kadal shrimp	metapenaeus dobsoni	shrimp
MQE	york shrimp	metapenaeus eboracensis	shrimp
MTG	fine shrimp	metapenaeus elegans	shrimp
ENS	endeavour shrimp	metapenaeus endeavouri	shrimp

MPE	greasyback shrimp	metapenaeus ensis	shrimp
MJS	emerald shrimp	metapenaeus insolitus	shrimp
MJE	middle shrimp	metapenaeus intermedius	shrimp
SHI	shiba shrimp	metapenaeus joyneri	shrimp
MJK	ginger shrimp	metapenaeus kutchensis	shrimp
MJY	bird shrimp	metapenaeus lysianassa	shrimp
MPM	eastern school shrimp	metapenaeus macleayi	shrimp
MPN	speckled shrimp	metapenaeus monoceros	shrimp
MMQ	moyebi shrimp	metapenaeus moyebi	shrimp
MTQ	papua shrimp	metapenaeus papuensis	shrimp
MET	metapenaeus shrimps nei	metapenaeus spp	shrimp
MNG	peregrine shrimp	metapenaeus stebbingi	shrimp
MSW	sulu shrimp	metapenaeus suluensis	shrimp
MQT	stork shrimp	metapenaeus tenuipes	shrimp
MVE	crimson coral shrimp	microprosthema semilaeve	shrimp
DCP	natantian decapods nei	natantia	shrimp
WJX		nematocarcinidae	shrimp
NCF	african spider shrimp	nematocarcinus africanus	shrimp
NCZ	spider shrimp	nematocarcinus agassizii	shrimp
NYX	spider prawns nei	nematocarcinus spp	shrimp
NLH	west african estuarine prawn	nematopalaemon hastatus	shrimp
NLC	whitebelly prawn	nematopalaemon schmitti	shrimp
NLN	spider prawn	nematopalaemon tenuipes	shrimp
NCM		notocrangon antarcticus	shrimp
NJP	japanese spinyridge	notostomus japonicus	shrimp
OGR	telescope shrimp	ogyrides orientalis	shrimp
VWX		oplophoridae	shrimp
QAX		oplophorus spp	shrimp
PAA	baltic prawn	palaemon adspersus	shrimp
MOZ	mangrove prawn	palaemon concinnus	shrimp
PNQ	rockpool prawn	palaemon elegans	shrimp
PVK	chinese ditch prawn	palaemon gravieri	shrimp
LHK	tropical river prawn	palaemon hancocki	shrimp
LHY	hilton shrimp	palaemon hiltoni	shrimp
PIQ	delta prawn	palaemon longirostris	shrimp
PMZ	migrant prawn	palaemon macrodactylus	shrimp
MMV	zaire prawn	palaemon maculatus	shrimp
ONQ	caribbean bait prawn	palaemon northropi	shrimp
OOJ	gladiator prawn	palaemon ortmanni	shrimp
OUI	indian bait prawn	palaemon pacificus	shrimp
OAP	potitinga prawn	palaemon pandaliformis	shrimp
PMW	lake prawn	palaemon paucidens	shrimp

LMW	barred grass shrimp	palaemon ritteri	shrimp
CPR	common prawn	palaemon serratus	shrimp
OSK	carpenter prawn	palaemon serrifer	shrimp
QPX	palaemon shrimps nei	palaemon spp	shrimp
OXH	posidonia prawn	palaemon xiphias	shrimp
LMQ	pond shrimp	palaemonetes antennarius	shrimp
LKK	mississippi grass shrimp	palaemonetes kadiakensis	shrimp
MPQ	eastern grass shrimp	palaemonetes paludosus	shrimp
LKI	lagoon shrimp	palaemonetes schmitti	shrimp
MSZ	chinese grass shrimp	palaemonetes sinensis	shrimp
TKZ	tonkin grass shrimp	palaemonetes tonkinensis	shrimp
PVR	atlantic ditch shrimp	palaemonetes varians	shrimp
PVV	marsh shrimp	palaemonetes vulgaris	shrimp
PZY	ebro shrimp	palaemonetes zariquieyi	shrimp
PPZ	freshwater prawns, shrimps nei	palaemonidae	shrimp
PAL	palaemonid shrimps nei	palaemonidae	shrimp
PDZ	pandalid shrimps nei	pandalidae	shrimp
NDD	sidestripe shrimp	pandalopsis dispar	shrimp
NDJ	morotoge shrimp	pandalopsis japonica	shrimp
NDP	pandalopsis shrimps nei	pandalopsis spp	shrimp
DLS	deep-water bigeye shrimp	pandalus amplus	shrimp
PRA	northern prawn	pandalus borealis	shrimp
DUD	dock shrimp	pandalus danae	shrimp
DUJ	humpy shrimp	pandalus goniurus	shrimp
PYX	coonstripe shrimp	pandalus hypsinotus	shrimp
PJK	ocean shrimp	pandalus jordani	shrimp
DUK	hokkai shrimp	pandalus kessleri	shrimp
AES	aesop shrimp	pandalus montagui	shrimp
DLN	botan shrimp	pandalus nipponensis	shrimp
PWY	spot shrimp	pandalus platyceros	shrimp
PAN	pandalus shrimps nei	pandalus spp	shrimp
PSH	pacific shrimps nei	pandalus spp, pandalopsis spp	shrimp
NTF	hinged shrimp	pantomus affinis	shrimp
GNZ	areolated horned shrimp	paracrangon areolata	shrimp
PVJ	narwal shrimp	parapandalus narval	shrimp
NDS	oriental narwal shrimp	parapandalus spinipes	shrimp
NAK	hawknose shrimp	parapenaeopsis acclivirostris	shrimp
NRJ	arafura shrimp	parapenaeopsis arafurica	shrimp
GUS	guinea shrimp	parapenaeopsis atlantica	shrimp
NIB	bally shrimp	parapenaeopsis balli	shrimp
NPN	coral shrimp	parapenaeopsis cornuta	shrimp
NPI	coromandel shrimp	parapenaeopsis coromandelica	shrimp

NIG	thin shrimp	parapenaeopsis gracillima	shrimp
NAW	spear shrimp	parapenaeopsis hardwickii	shrimp
NAH	dog shrimp	parapenaeopsis hungerfordi	shrimp
NAX	torpedo shrimp	parapenaeopsis maxillipedo	shrimp
NIN	dwarf shrimp	parapenaeopsis nana	shrimp
NPB	parole shrimp	parapenaeopsis probata	shrimp
NAP	rainbow shrimp	parapenaeopsis sculptilis	shrimp
NPP	parapenaeopsis shrimps nei	parapenaeopsis spp	shrimp
NAY	kiddi shrimp	parapenaeopsis stylifera	shrimp
NIT	smoothshell shrimp	parapenaeopsis tenella	shrimp
NIU	uncta shrimp	parapenaeopsis uncta	shrimp
NIV	adonis shrimp	parapenaeopsis venusta	shrimp
NRA	australian rose shrimp	parapenaeus australiensis	shrimp
NRF	neptune rose shrimp	parapenaeus fissurus	shrimp
NRI	explorer rose shrimp	parapenaeus investigatoris	shrimp
NRK	lancer rose shrimp	parapenaeus lanceolatus	shrimp
NRG	flamingo shrimp	parapenaeus longipes	shrimp
DPS	deep-water rose shrimp	parapenaeus longirostris	shrimp
NRP	rose shrimp	parapenaeus politus	shrimp
NRR	domino shrimp	parapenaeus sextuberculatus	shrimp
NRY	parapenaeus shrimps nei	parapenaeus spp	shrimp
YKO	nuka shrimp	paratya compressa	shrimp
FAJ	japanese glass shrimp	pasiphaea japonica	shrimp
FAM	pink glass shrimp	pasiphaea multidentata	shrimp
FAV	white glass shrimp	pasiphaea sivado	shrimp
FAC	crimson pasiphaeid	pasiphaea tarda	shrimp
PEZ	penaeid shrimps nei	penaeidae	shrimp
NIA	scythe shrimp	penaeopsis balssi	shrimp
NIE	four-spined needle shrimp	penaeopsis eduardoi	shrimp
PJE	needle shrimp	penaeopsis rectacuta	shrimp
NIS	megalops shrimp	penaeopsis serrata	shrimp
ABS	northern brown shrimp	penaeus aztecus	shrimp
PNB	redspotted shrimp	penaeus brasiliensis	shrimp
CSP	crystal shrimp	penaeus brevirostris	shrimp
YPS	yellowleg shrimp	penaeus californiensis	shrimp
EKU	witch prawn	penaeus canaliculatus	shrimp
FLP	fleshy prawn	penaeus chinensis	shrimp
APS	northern pink shrimp	penaeus duorarum	shrimp
PRB	brown tiger prawn	penaeus esculentus	shrimp
PNI	indian white prawn	penaeus indicus	shrimp
KUP	kuruma prawn	penaeus japonicus	shrimp
TGS	caramote prawn	penaeus kerathurus	shrimp

WKP	western king prawn	penaeus latisulcatus	shrimp
ELY	red-spot king prawn	penaeus longistylus	shrimp
PNJ	aloha prawn	penaeus marginatus	shrimp
PBA	banana prawn	penaeus merguiensis	shrimp
GIT	giant tiger prawn	penaeus monodon	shrimp
SOP	southern pink shrimp	penaeus notialis	shrimp
WWP	western white shrimp	penaeus occidentalis	shrimp
PPS	sao paulo shrimp	penaeus paulensis	shrimp
REP	redtail prawn	penaeus penicillatus	shrimp
PNP	eastern king prawn	penaeus plebejus	shrimp
PNT	southern white shrimp	penaeus schmitti	shrimp
TIP	green tiger prawn	penaeus semisulcatus	shrimp
PST	northern white shrimp	penaeus setiferus	shrimp
ESS	false white prawn	penaeus silasi	shrimp
PEN	penaeus shrimps nei	penaeus spp	shrimp
PNS	blue shrimp	penaeus stylirostris	shrimp
PNU	southern brown shrimp	penaeus subtilis	shrimp
PNV	whiteleg shrimp	penaeus vannamei	shrimp
LAA	argentine red shrimp	pleoticus muelleri	shrimp
RRS	royal red shrimp	pleoticus robustus	shrimp
WPX	pleoticus shrimps nei	pleoticus spp	shrimp
LKC	lesser striped shrimp	plesionika acanthonotus	shrimp
LKL	gondwana striped shrimp	plesionika alcocki	shrimp
LKN	catalonian striped shrimp	plesionika antigai	shrimp
EKB	scarlet longbeak shrimp	plesionika beebei	shrimp
EKO	giant longbeak shrimp	plesionika carinirostris	shrimp
LKW	striped soldier shrimp	plesionika edwardsii	shrimp
LKS	striped gladiator shrimp	plesionika ensis	shrimp
LKJ	italian deep-sea shrimp	plesionika gigliolii	shrimp
LKO	arrow shrimp	plesionika heterocarpus	shrimp
LKT	golden shrimp	plesionika martia	shrimp
EKX	mexican longbeak shrimp	plesionika mexicana	shrimp
XKX	plesionika shrimps nei	plesionika spp	shrimp
EKT	colombian longbeak shrimp	plesionika trispinus	shrimp
EKW	guinea striped shrimp	plesionika williamsi	shrimp
SSH	scarlet shrimp	plesiopenaeus edwardsianus	shrimp
ONZ	hardshell shrimp	pontocaris lacazei	shrimp
PKQ	feather shrimp	pontocaris pennata	shrimp
OFI	spiny shrimp	pontophilus spinosus	shrimp
RKU	processa shrimp	processa canaliculata	shrimp
RKD	nika shrimp	processa edulis	shrimp

RKJ	peruvian one-handed shrimp	processa peruviana	shrimp
RXK	processa shrimps nei	processa spp	shrimp
RRJ	titi shrimp	protrachypene precipua	shrimp
RCY	striped hinge beak shrimp	rhynchocinetes durbanensis	shrimp
RYW	rabbitnose shrimp	rhynchocinetes typus	shrimp
RNQ	spotted marbled shrimp	saron neglectus	shrimp
SJX	four-spines nutshell shrimp	sclerocrangon atrox	shrimp
EGL	bering shrimp	sclerocrangon salebrosa	shrimp
CVL	sculptured shrimps nei	sclerocrangon spp	shrimp
GTN	sakura shrimp	sergestes lucens	shrimp
SHS	sergestid shrimps nei	sergestidae	shrimp
YIF	coloured rock shrimp	sicyonia affinis	shrimp
YIN	hardhusk rock shrimp	sicyonia aliaffinis	shrimp
RSH	rock shrimp	sicyonia brevirostris	shrimp
YIB	burkenroad's rock shrimp	sicyonia burkenroadi	shrimp
YIA	mediterranean rock shrimp	sicyonia carinata	shrimp
YIC	ridgeback rock shrimp	sicyonia cristata	shrimp
YIO	keeled rock shrimp	sicyonia disdorsalis	shrimp
YIW	target shrimp	sicyonia disedwardsi	shrimp
YIR	notched rock shrimp	sicyonia disparri	shrimp
YID	lesser rock shrimp	sicyonia dorsalis	shrimp
YIG	tufted rock shrimp	sicyonia galeata	shrimp
YII	pacific rock shrimp	sicyonia ingentis	shrimp
YIV	notched tidal rock shrimp	sicyonia laevigata	shrimp
YIL	knight rock shrimp	sicyonia lancifera	shrimp
YIM	martin's rock shrimp	sicyonia martini	shrimp
YIX	mixed rock shrimp	sicyonia mixta	shrimp
YIP	peanut rock shrimp	sicyonia penicillata	shrimp
YIK	small peanut rock	sicyonia picta	shrimp
YIS	eyespot rock shrimp	sicyonia stimpsoni	shrimp
YIT	kinglet rock shrimp	sicyonia typica	shrimp
ONJ		solenocera acuminata	shrimp
SKF	african mud shrimp	solenocera africana	shrimp
SOK	kolibri shrimp	solenocera agassizii	shrimp
SAZ	deep-water mud shrimp	solenocera alfonso	shrimp
OAJ	algoa shrimp	solenocera algoensis	shrimp
SJR	high ridge mud shrimp	solenocera alticarinata	shrimp
SKQ	australia mus shrimp	solenocera australiana	shrimp
SKK	ridgeback shrimp	solenocera choprai	shrimp
SOJ	coastal mud shrimp	solenocera crassicornis	shrimp
SJO	flower shrimp	solenocera florea	shrimp

SJK	guiana mud shrimp	solenocera geijskesi	shrimp
SKW	malayan mud shrimp	solenocera halli	shrimp
SXT	deep-sea mud shrimp	solenocera hextii	shrimp
SKO	chinese mud shrimp	solenocera koelbeli	shrimp
SQH	razor mud shrimp	solenocera melantho	shrimp
SKM	atlantic mud shrimp	solenocera membranacea	shrimp
SMZ	blossom shrimp	solenocera mutator	shrimp
SKE	comb shrimp	solenocera pectinata	shrimp
SJQ	false comb shrimp	solenocera pectinulata	shrimp
SZK	solenocera shrimps nei	solenocera spp	shrimp
SOZ	solenocerid shrimps nei	solenoceridae	shrimp
IRI	friendly spine shrimp	spirontocaris lilljeborgii	shrimp
IRS	parrot shrimp	spirontocaris spinus	shrimp
TPD	banded coral shrimp	stenopus hispidus	shrimp
YSJ	quayle spinytail	systellaspis braueri	shrimp
TYK	hardback shrimp	trachypenaeus anchoralis	shrimp
TVU	smooth shrimp	trachypenaeus brevisuturae	shrimp
TBY	carabali shrimp	trachypenaeus byrdi	shrimp
TKN	roughneck shrimp	trachypenaeus constrictus	shrimp
TRV	southern rough shrimp	trachypenaeus curvirostris	shrimp
TFO	indio shrimp	trachypenaeus faoe	shrimp
TFV	brown rough shrimp	trachypenaeus fulvus	shrimp
TFJ	pinto shrimp	trachypenaeus fuscina	shrimp
TCQ	northern rough shrimp	trachypenaeus gonospinifer	shrimp
TPQ	coarse shrimp	trachypenaeus granulosus	shrimp
TLJ	longlegged rough shrimp	trachypenaeus longipes	shrimp
TKJ	zebra shrimp	trachypenaeus pacificus	shrimp
TDZ	malayan rough shrimp	trachypenaeus sedili	shrimp
TMY	yellow roughneck shrimp	trachypenaeus similis	shrimp
YEU	trachypenaeus shrimps nei	trachypenaeus spp	shrimp
TVZ	philippines rough shrimp	trachypenaeus villaluzi	shrimp
BOB	atlantic seabob	xiphopenaeus kroyeri	shrimp
TIT	pacific seabob	xiphopenaeus riveti	shrimp
XFS	xiphopenaeus shrimps nei	xiphopenaeus spp	shrimp
BOS	pacific seabobs	xiphopenaeus, trachypenaeus spp	shrimp
SWO	swordfish	xiphias gladius	swordfish
SKJ	skipjack tuna	katsuwonus pelamis	skipjack_tuna
ALB	albacore	thunnus alalunga	albacore_tuna
YFT	yellowfin tuna	thunnus albacares	yellowfin_tuna
SBF	southern bluefin tuna	thunnus maccoyii	bluefin_tuna
BET	bigeye tuna	thunnus obesus	bigeye_tuna
PBF	pacific bluefin tuna	thunnus orientalis	bluefin_tuna
BFT	atlantic bluefin tuna	thunnus thynnus	bluefin_tuna

TUN	tunas nei	thunnini	other_tuna
TUS	true tunas nei	thunnus spp	other_tuna

Table S13: Mapping of reported (2018) HTS codes to consistent (and usually less-refined) 2016 HTS codes used to link the 2018 ITC report data to 2016 import data. Also included is the mapping from reported and 2016 HTS codes to NMFS species groups.

NMFS Species Group	*Product Name*	*Reported HTS Code*	*2016 HTS Code*
abalone	ABALONE DRIED/SALTED/BRINE	0307870000	0307890000
abalone	ABALONE FROZEN	0307830000	0307890000
abalone	ABALONE FROZEN/DRIED/SALTED/BRINE	0307890000	0307890000
abalone	ABALONE LIVE/FRESH	0307810000	0307810000
abalone	ABALONE PREPARED/PRESERVED	1605576000	1605576000
abalone	ABALONE PRODUCTS PREPARED DINNERS	1605570500	1605570500
albacore_tuna	TUNA ALBACORE FRESH	0302310000	0302310000
albacore_tuna	TUNA ALBACORE FROZEN	0303410000	0303410000
albacore_tuna	TUNA ALBACORE IN ATC (FOIL OR FLEXIBLE) NOT IN OIL IN QUOTA	1604142251	1604142251
albacore_tuna	TUNA ALBACORE IN ATC (FOIL OR FLEXIBLE) NOT IN OIL OVER QUOTA	1604143051	1604143051
albacore_tuna	TUNA ALBACORE IN ATC (OTHER) IN OIL	1604141091	1604141091
albacore_tuna	TUNA ALBACORE IN ATC (OTHER) NOT IN OIL IN QUOTA	1604142259	1604142259
albacore_tuna	TUNA ALBACORE IN ATC (OTHER) NOT IN OIL OVER QUOTA	1604143059	1604143059
anchovy	ANCHOVY CANNED IN OIL	1604162000	1604162000
anchovy	ANCHOVY CANNED NOT IN OIL > 6.8KG	1604166000	1604166000
anchovy	ANCHOVY CANNED NOT IN OIL NOT > 6.8KG	1604164000	1604164000
anchovy	ANCHOVY FRESH	0302420000	0302420000
anchovy	ANCHOVY SALTED > 6.8KG	0305636000	0305636000
anchovy	ANCHOVY SALTED IN ATC NOT > 6.8KG	0305632000	0305632000
anchovy	ANCHOVY SALTED NOT IN ATC NOT > 6.8KG	0305634000	0305634000
atka mackerel	ATKA MACKEREL FRESH	0302895070	0302895070
atka mackerel	ATKA MACKEREL FROZEN	0303890046	0303890046
atlantic_salmon	SALMON ATLANTIC FILLET FRESH FARMED	0304410010	0304410010
atlantic_salmon	SALMON ATLANTIC FILLET FRESH WILD	0304410020	0304410020
atlantic_salmon	SALMON ATLANTIC FILLET FROZEN	0304815010	0304815010
atlantic_salmon	SALMON ATLANTIC FRESH FARMED	0302140003	0302140003
atlantic_salmon	SALMON ATLANTIC FRESH WILD	0302140004	0302140004
atlantic_salmon	SALMON ATLANTIC MEAT FRESH FARMED	0304520010	0304520010
atlantic_salmon	SALMON ATLANTIC MEAT FRESH WILD	0304520015	0304520015
atlantic_salmon	SALMON ATLANTIC,DANUBE FRESH	0302140062	0302140062
atlantic_salmon	SALMON ATLANTIC,DANUBE FROZEN	0303130000	0303130000
bass	BASS FRESH	0302895028	0302895028
bigeye_tuna	TUNA BIGEYE FRESH	0302340000	0302340000
bigeye_tuna	TUNA BIGEYE FROZEN	0303440000	0303440000

blue whiting_groundfish	GROUNDFISH BLUE WHITING FRESH	0302565000	0302565000
blue whiting_groundfish	GROUNDFISH BLUE WHITING FROZEN	0303680000	0303680000
blue_crab	CRABMEAT SWIMMING (CALLINECTES) FROZEN	1605104025	1605104025
blue_crab	CRABMEAT SWIMMING (CALLINECTES) IN ATC	1605102051	1605102051
bluefin_tuna	TUNA BLUEFIN ATLANTIC FROZEN	0303450110	0303450110
bluefin_tuna	TUNA BLUEFIN ATLANTIC,PACIFIC FRESH	0302350100	0302350100
bluefin_tuna	TUNA BLUEFIN PACIFIC FROZEN	0303450150	0303450150
bluefin_tuna	TUNA BLUEFIN SOUTHERN FRESH	0302360000	0302360000
bluefin_tuna	TUNA BLUEFIN SOUTHERN FROZEN	0303460000	0303460000
bonito	BONITO CANNED IN OIL	1604147000	1604147000
bonito	BONITO CANNED NOT IN OIL	1604148000	1604148000
bonito	BONITO,YELLOWTAIL,POLLOCK CANNED IN OIL	1604192500	1604192500
bonito	BONITO,YELLOWTAIL,POLLOCK CANNED NOT IN OIL	1604191000	1604191000
butterfish	BUTTERFISH FROZEN	0303890055	0303890055
capelin	CAPELIN FROZEN	0303890058	0303890058
carp	CARP NSPF FRESH NSPF	0302735000	0302735000
carp	CARP NSPF FROZEN	0303250000	0303250000
carp	CARP NSPF FROZEN	0303250100	0303250000
catfish	CATFISH (ICTALURUS) FILLET FRESH	0304320010	0304320010
catfish	CATFISH (ICTALURUS) FILLET FROZEN	0304620010	0304620010
catfish	CATFISH (ICTALURUS) FRESH	0302725001	0302725001
catfish	CATFISH (ICTALURUS) FROZEN	0303240010	0303240010
catfish	CATFISH (ICTALURUS) MEAT FRESH	0304510010	0304510010
catfish	CATFISH (ICTALURUS,PANGASIUS,SILURUS) FRESH NOT > 6.8KG	0302721100	0302721100
catfish	CATFISH (PANGASIUS) FILLET FRESH	0304320020	0304320020
catfish	CATFISH (PANGASIUS) FILLET FROZEN	0304620020	0304620020
catfish	CATFISH (PANGASIUS) FRESH	0302725004	0302725004
catfish	CATFISH (PANGASIUS) FROZEN	0303240020	0303240020
catfish	CATFISH (PANGASIUS) MEAT FRESH	0304510015	0304510015
catfish	CATFISH (SILURIFORMES OTHER) FILLET FROZEN	0304620030	0304620030
catfish	CATFISH (SILURUS,CLARIAS) FILLET FRESH	0304320090	0304320090
catfish	CATFISH NSPF FILLET FROZEN	0304620090	0304620090
catfish	CATFISH NSPF FRESH	0302725034	0302725034
catfish	CATFISH NSPF FROZEN	0303240050	0303240050
chinook_salmon	SALMON CHINOOK FRESH FARMED	0302130013	0302130013
chinook_salmon	SALMON CHINOOK FRESH WILD	0302130014	0302130014
chinook_salmon	SALMON CHINOOK FROZEN	0303120012	0303120012
chum_salmon	SALMON CHUM CANNED NOT IN OIL	1604114010	1604114010
chum_salmon	SALMON CHUM FRESH	0302130022	0302130022
chum_salmon	SALMON CHUM FROZEN	0303120022	0303120022

clam	CLAM GEODUCK DRIED/SALTED/BRINE	0307790130	0307790030
clam	CLAM GEODUCK FROZEN	0307720030	0307790030
clam	CLAM GEODUCK FROZEN/DRIED/SALTED/BRINE	0307790030	0307790030
clam	CLAM GEODUCK LIVE/FRESH	0307710050	0307710050
clam	CLAM NSPF BOILED CANNED WHETHER OR NOT MINCED/CHOP > 0.68KG	1605562000	1605562000
clam	CLAM NSPF BOILED CANNED WHETHER OR NOT MINCED/CHOP NOT > 0.68KG	1605561500	1605561500
clam	CLAM NSPF DRIED/SALTED/BRINE	0307790155	0307790055
clam	CLAM NSPF FROZEN	0307720055	0307790055
clam	CLAM NSPF FROZEN/DRIED/SALTED/BRINE	0307790055	0307790055
clam	CLAM NSPF LIVE/FRESH	0307710070	0307710070
clam	CLAM NSPF PREPARED/PRESERVED	1605563000	1605563000
clam	CLAM RAZOR CANNED	1605561000	1605561000
clam	CLAM STIMSON FROZEN/DRIED/SALTED/BRINE	0307790051	0307790051
cobia	COBIA FRESH	0302465000	0302465000
cobia	COBIA FRESH NOT >6.8KG	0302461100	0302461100
cobia	COBIA FROZEN	0303560000	0303560000
cockles	COCKLES,ARK SHELLS NSPF DRIED/SALTED/BRINE	0307790160	0307790060
cockles	COCKLES,ARK SHELLS NSPF FROZEN	0307720060	0307790060
cockles	COCKLES,ARK SHELLS NSPF FROZEN/DRIED/SALTED/BRINE	0307790060	0307790060
cockles	COCKLES,ARK SHELLS NSPF LIVE/FRESH	0307710090	0307710090
cockles	COCKLES,ARK SHELLS NSPF PREPARED/PRESERVED	1605566000	1605566000
cod_groundfish	GROUNDFISH COD ATLANTIC FILLET FRESH	0304440010	0304440010
cod_groundfish	GROUNDFISH COD ATLANTIC FRESH	0302510010	0302510010
cod_groundfish	GROUNDFISH COD ATLANTIC FROZEN	0303630010	0303630010
cod_groundfish	GROUNDFISH COD ATLANTIC MEAT FRESH	0304530010	0304530010
cod_groundfish	GROUNDFISH COD NSPF DRIED	0305510000	0305510000
cod_groundfish	GROUNDFISH COD NSPF FILLET BLOCKS FROZEN > 4.5KG	0304711000	0304711000
cod_groundfish	GROUNDFISH COD NSPF FILLET DRIED/SALTED/BRINE	0305320010	0305320010
cod_groundfish	GROUNDFISH COD NSPF FILLET FRESH	0304440015	0304440015
cod_groundfish	GROUNDFISH COD NSPF FILLET FROZEN	0304715000	0304715000
cod_groundfish	GROUNDFISH COD NSPF FILLET SALTED MOISTURE > 50%	0305620050	0305620050
cod_groundfish	GROUNDFISH COD NSPF FILLET SALTED MOISTURE CONTENT BET 45-50%	0305620060	0305620060
cod_groundfish	GROUNDFISH COD NSPF FILLET SALTED MOISTURE NOT > 43%	0305620080	0305620080
cod_groundfish	GROUNDFISH COD NSPF FRESH	0302510090	0302510090
cod_groundfish	GROUNDFISH COD NSPF FROZEN	0303630090	0303630090
cod_groundfish	GROUNDFISH COD NSPF MEAT FRESH	0304530015	0304530015

cod_groundfish	GROUNDFISH COD NSPF MEAT FROZEN > 6.8KG	0304951020	0304951020
cod_groundfish	GROUNDFISH COD NSPF MINCED FROZEN > 6.8KG	0304951010	0304951010
cod_groundfish	GROUNDFISH COD NSPF SALTED MOISTURE CONTENT > 50%	0305620010	0305620010
cod_groundfish	GROUNDFISH COD NSPF SALTED MOISTURE CONTENT BET 43-45%	0305620030	0305620025
cod_groundfish	GROUNDFISH COD NSPF SALTED MOISTURE CONTENT BET 45-50%	0305620025	0305620025
cod_groundfish	GROUNDFISH COD NSPF SALTED MOISTURE CONTENT NOT > 43%	0305620045	0305620045
cod_groundfish	GROUNDFISH COD,CUSK,HADDOCK,HAKE,POLLOCK SMOKED	0305494020	0305494020
coho_salmon	SALMON COHO FRESH FARMED	0302130053	0302130053
coho_salmon	SALMON COHO FRESH WILD	0302130054	0302130054
coho_salmon	SALMON COHO FROZEN	0303120052	0303120052
conch	CONCH LIVE/FRESH	0307910130	0307910130
conch	CONCH NSPF FROZEN	0307920030	0307910130
conch	CONCH NSPF LIVE/FRESH	0307910230	0307910130
conch	STROMBOID CONCHS (STROMBUS SPP.) DRIED/SALTED/BRINE	0307880000	0307910130
conch	STROMBOID CONCHS (STROMBUS SPP.) FROZEN	0307840000	0307910130
conch	STROMBOID CONCHS (STROMBUS SPP.) LIVE/FRESH	0307820000	0307910130
crawfish	CRAWFISH FRESHWATER FROZEN	0306190010	0306190010
crawfish	CRAWFISH FRESHWATER PEELED	1605401010	1605401010
cusk_groundfish	GROUNDFISH CUSK FILLET FRESH	0304490125	0302895040
cusk_groundfish	GROUNDFISH CUSK FILLET FROZEN	0304895060	0304895060
cusk_groundfish	GROUNDFISH CUSK FRESH	0302895040	0302895040
cusk_groundfish	GROUNDFISH CUSK FROZEN	0303890010	0303890010
cusk_groundfish	GROUNDFISH CUSK MEAT FRESH	0304590070	0304590070
cusk_groundfish	GROUNDFISH CUSK,HADDOCK FILLET SALTED	0305691049	0305691049
cusk_groundfish	GROUNDFISH CUSK,HADDOCK WHOLE/DRESSED SALTED	0305691029	0305691029
cuttlefish	CUTTLEFISH DRIED/SALTED/BRINE	0307490160	0307490060
cuttlefish	CUTTLEFISH FROZEN	0307430060	0307490060
cuttlefish	CUTTLEFISH FROZEN/DRIED/SALTED/BRINE	0307490060	0307490060
cuttlefish	CUTTLEFISH LIVE/FRESH	0307410060	0307410060
cuttlefish	CUTTLEFISH LIVE/FRESH	0307420060	0307410060
cuttlefish	CUTTLEFISH NSPF PREPARED/PRESERVED	1605546010	1605546010
dolphin	DOLPHINFISH FILLET FROZEN	0304895055	0304895055
dolphin	DOLPHINFISH FRESH	0302895072	0302895072
dungeness_crab	CRAB DUNGENESS FROZEN	0306144030	0306144030
dungeness_crab	CRABMEAT DUNGENESS FROZEN	1605104015	1605104015
dungeness_crab	CRABMEAT DUNGENESS IN ATC	1605102030	1605102030
eels	EELS FRESH	0302740000	0302740000

eels	EELS FROZEN	0303260000	0303260000
eels	EELS IN ATC NOT IN OIL	1604171000	1604171000
eels	EELS IN OIL >7KG	1604176000	1604176000
eels	EELS IN OIL NOT >7KG	1604178000	1604178000
flounder_flatfish	FLATFISH FLOUNDER FILLET BLOCKS FROZEN > 4.5KG	0304831020	0304831020
flounder_flatfish	FLATFISH FLOUNDER FILLET FRESH	0304430020	0304430020
flounder_flatfish	FLATFISH FLOUNDER FILLET FROZEN	0304835020	0304835020
flounder_flatfish	FLATFISH FLOUNDER FRESH	0302290110	0302290110
flounder_flatfish	FLATFISH FLOUNDER FROZEN	0303390110	0303390110
flounder_flatfish	FLATFISH FLOUNDER MEAT FRESH	0304590050	0304590050
grouper	GROUPER FRESH	0302895061	0302895061
grouper	GROUPER FROZEN	0303890070	0303890070
haddock_groundfish	GROUNDFISH HADDOCK FILLET BLOCKS FROZEN > 4.5KG	0304721000	0304721000
haddock_groundfish	GROUNDFISH HADDOCK FILLET FRESH	0304440020	0304440020
haddock_groundfish	GROUNDFISH HADDOCK FILLET FROZEN	0304725000	0304725000
haddock_groundfish	GROUNDFISH HADDOCK FRESH	0302520000	0302520000
haddock_groundfish	GROUNDFISH HADDOCK FROZEN	0303640000	0303640000
haddock_groundfish	GROUNDFISH HADDOCK MEAT FRESH	0304530020	0304530020
haddock_groundfish	GROUNDFISH HADDOCK MEAT FROZEN > 6.8KG	0304951025	0304951025
hake_groundfish	GROUNDFISH HAKE FILLET BLOCKS FROZEN > 4.5KG	0304741000	0304741000
hake_groundfish	GROUNDFISH HAKE FILLET FRESH	0304440030	0304440030
hake_groundfish	GROUNDFISH HAKE FILLET FROZEN	0304745000	0304745000
hake_groundfish	GROUNDFISH HAKE FILLET SALTED	0305691041	0305691041
hake_groundfish	GROUNDFISH HAKE FRESH	0302545000	0302545000
hake_groundfish	GROUNDFISH HAKE FRESH NOT >6.8KG	0302541100	0302541100
hake_groundfish	GROUNDFISH HAKE MEAT FRESH	0304530030	0304530030
hake_groundfish	GROUNDFISH HAKE WHOLE/DRESSED SALTED	0305691021	0305691021
hake_groundfish	GROUNDFISH HAKE,WHITING FROZEN	0303660000	0303660000
halibut_flatfish	FLATFISH HALIBUT ATLANTIC FRESH	0302210010	0302210010
halibut_flatfish	FLATFISH HALIBUT ATLANTIC FROZEN	0303310010	0303310010
halibut_flatfish	FLATFISH HALIBUT NSPF FILLET BLOCKS FROZEN > 4.5KG	0304831025	0304831025
halibut_flatfish	FLATFISH HALIBUT NSPF FILLET FROZEN	0304835025	0304835025
halibut_flatfish	FLATFISH HALIBUT PACIFIC FRESH	0302210020	0302210020
halibut_flatfish	FLATFISH HALIBUT PACIFIC FROZEN	0303310020	0303310020
herring	HERRING FILLET DRIED/SALTED/BRINE > 6.8KG	0305396010	0305396010
herring	HERRING FILLET DRIED/SALTED/BRINE > 6.8KG	0305396110	0305396010
herring	HERRING FILLET DRIED/SALTED/BRINE NOT > 6.8KG	0305392000	0305392000
herring	HERRING FILLET FROZEN	0304860000	0304860000
herring	HERRING FRESH	0302410000	0302410000
herring	HERRING FROZEN	0303510000	0303510000
herring	HERRING IN ATC IN OIL	1604122000	1604122000

herring	HERRING IN TOMATO SAUCE/SMOKED/KIPPERED > 0.45KG	1604124000	1604124000
herring	HERRING KIPPERED	1604126050	1604126050
herring	HERRING PICKLED	1604126030	1604126030
herring	HERRING PICKLED FILLET	1604126010	1604126010
herring	HERRING PREPARED/PRESERVED	1604126090	1604126090
herring	HERRING SALTED > 6.8KG	0305614000	0305614000
herring	HERRING SALTED NOT > 6.8KG	0305612000	0305612000
herring	HERRING SMOKED FILLET	0305420060	0305420060
herring	HERRING SMOKED FILLET BONELESS	0305420050	0305420050
herring	HERRING SMOKED WHOLE OR BEHEADED NOT OTHERWISE PROCESSED	0305420020	0305420020
horse mackerel_jack	JACK,HORSE MACKEREL FRESH	0302455000	0302455000
horse mackerel_jack	JACK,HORSE MACKEREL FRESH NOT > 6.8KG	0302451100	0302451100
horse mackerel_jack	JACK,HORSE MACKEREL FROZEN	0303550000	0303550000
jellyfish	JELLYFISH (RHOPILEMA SPP.) LIVE/FRESH/FROZEN/DRIED/SALTED/BRINE/SMOKED	0308300000	0308300000
jellyfish	JELLYFISH PREPARED/PRESERVED	1605630000	1605630000
king_crab	CRAB BLUE KING (PARALITHODES PLATYPUS) FROZEN	0306144006	0306144010
king_crab	CRAB GOLDEN/BROWN KING (LITHODES AEQUISPINUS) FROZEN	0306144009	0306144010
king_crab	CRAB KING FROZEN	0306144010	0306144010
king_crab	CRAB KING OTHER FROZEN	0306144015	0306144010
king_crab	CRAB RED KING (PARALITHODES CAMTSCHATICUS) FROZEN	0306144003	0306144010
king_crab	CRAB SOUTHERN RED SANTOLLA KING (LITHODES SANTOLLA)FROZEN	0306144012	0306144010
king_crab	CRABMEAT KING FROZEN	1605104002	1605104002
king_crab	CRABMEAT KING IN ATC	1605102010	1605102010
krill	KRILL ANTARCTIC	0306190030	0306190030
lingcod	LINGCOD FRESH	0302895064	0302895064
lobster	LOBSTER (HOMARUS SPP.) FRESH	0306320090	0306220090
lobster	LOBSTER (HOMARUS SPP.) FRESH/DRIED/SALTED/BRINE	0306220090	0306220090
lobster	LOBSTER (HOMARUS SPP.) FROZEN	0306120070	0306120070
lobster	LOBSTER (HOMARUS SPP.) FROZEN IN ATC	0306120030	0306120030
lobster	LOBSTER (HOMARUS SPP.) FROZEN IN ATC IN BRINE	0306120010	0306120010
lobster	LOBSTER (HOMARUS SPP.) FROZEN IN BRINE	0306120050	0306120050
lobster	LOBSTER (HOMARUS SPP.) LIVE	0306220010	0306220010
lobster	LOBSTER (HOMARUS SPP.) LIVE	0306320010	0306220010
lobster	LOBSTER (HOMARUS SPP.)DRIED/SALTED/BRINE	0306920000	0306220090
lobster	LOBSTER NORWAY DRIED/SALTED/BRINE	0306940000	0306250000
lobster	LOBSTER NORWAY FROZEN	0306150000	0306150000

lobster	LOBSTER NORWAY LIVE/FRESH	0306340000	0306250000
lobster	LOBSTER NORWAY LIVE/FRESH/SALTED/BRINE	0306250000	0306250000
lobster	LOBSTER NSPF MEAT COOKED CHILLED	1605301030	1605301030
lobster	LOBSTER NSPF MEAT COOKED FROZEN	1605301010	1605301010
lobster	LOBSTER NSPF MEAT COOKED IN ATC	1605301050	1605301050
lobster	LOBSTER NSPF MEAT COOKED OTHER PREPARATIONS	1605301090	1605301090
lobster	LOBSTER NSPF PRODUCTS PREPARED DINNERS IN ATC	1605300510	1605300510
lobster	LOBSTER NSPF PRODUCTS PREPARED DINNERS NOT IN ATC	1605300590	1605300590
lobster	LOBSTER ROCK CARIBBEAN SPINY FROZEN	0306110010	0306110010
lobster	LOBSTER ROCK NSPF DRIED/SALTED/BRINE	0306910000	0306210000
lobster	LOBSTER ROCK NSPF FROZEN	0306110020	0306110020
lobster	LOBSTER ROCK NSPF LIVE/FRESH	0306310000	0306210000
lobster	LOBSTER ROCK NSPF LIVE/FRESH/DRIED/SALTED/BRINE	0306210000	0306210000
mackerel	MACKEREL FILLET DRIED/SALTED/BRINE NOT > 6.8KG	0305394000	0305394000
mackerel	MACKEREL FRESH	0302440000	0302440000
mackerel	MACKEREL FROZEN	0303540000	0303540000
mackerel	MACKEREL PREPARED/PRESERVED	1604150000	1604150000
mackerel	MACKEREL SALTED > 6.8KG	0305693000	0305693000
mackerel	MACKEREL SALTED NOT > 6.8KG	0305692000	0305692000
mackerel	MACKEREL SMOKED	0305492000	0305492000
monkfish	MONKFISH FRESH	0302895067	0302895067
monkfish	MONKFISH FROZEN	0303890052	0303890052
mullet	MULLET FROZEN	0303890049	0303890049
mussels	MUSSELS DRIED/SALTED/BRINE	0307390100	0307390000
mussels	MUSSELS FROZEN	0307320000	0307390000
mussels	MUSSELS FROZEN/DRIED/SALTED/BRINE	0307390000	0307390000
mussels	MUSSELS LIVE/FRESH FARMED	0307310010	0307310010
mussels	MUSSELS LIVE/FRESH WILD	0307310090	0307310090
mussels	MUSSELS NSPF PREPARED/PRESERVED	1605536000	1605536000
mussels	MUSSELS PRODUCTS PREPARED DINNERS	1605530500	1605530500
nile perch	NILE PERCH FILLET FRESH	0304330000	0304330000
nile perch	NILE PERCH FILLET FROZEN	0304630000	0304630000
nile perch	NILE PERCH FRESH	0302795025	0302795025
nile perch	NILE PERCH FROZEN	0303290110	0303290110
nile perch	NILE PERCH MEAT FRESH	0304510020	0304510020
nile perch	NILE PERCH MEAT FRESH	0304510120	0304510020
ocean perch_groundfish	GROUNDFISH OCEAN PERCH ATLANTIC FILLET BLOCKS FROZEN > 4.5KG	0304891025	0304891025

ocean perch_groundfish	GROUNDFISH OCEAN PERCH ATLANTIC FILLET FRESH	0304490020	0304490020
ocean perch_groundfish	GROUNDFISH OCEAN PERCH ATLANTIC FILLET FRESH	0304490120	0304490020
ocean perch_groundfish	GROUNDFISH OCEAN PERCH ATLANTIC FILLET FROZEN	0304895035	0304895035
ocean perch_groundfish	GROUNDFISH OCEAN PERCH ATLANTIC MEAT FRESH	0304590065	0304590065
ocean perch_groundfish	GROUNDFISH OCEAN PERCH NSPF MEAT FROZEN > 6.8KG	0304991170	0304991170
ocean perch_groundfish	GROUNDFISH OCEAN PERCH NSPF FILLET BLOCKS FROZEN > 4.5KG	0304891030	0304891030
ocean perch_groundfish	GROUNDFISH OCEAN PERCH NSPF FILLET FROZEN	0304895040	0304895040
ocean perch_groundfish	GROUNDFISH OCEAN PERCH NSPF FRESH	0302895055	0302895055
ocean perch_groundfish	GROUNDFISH OCEAN PERCH NSPF FROZEN	0303890064	0303890064
octopus	OCTOPUS DRIED/SALTED/BRINE	0307590100	0307590000
octopus	OCTOPUS FROZEN	0307520000	0307590000
octopus	OCTOPUS FROZEN/DRIED/SALTED/BRINE	0307590000	0307590000
octopus	OCTOPUS LIVE/FRESH	0307510000	0307510000
octopus	OCTOPUS NSPF PREPARED/PRESERVED	1605556000	1605556000
octopus	OCTOPUS PRODUCTS PREPARED DINNERS	1605550500	1605550500
orange roughy	ORANGE ROUGHY FILLET FROZEN	0304895050	0304895050
other aquatic invertebrates	AQUATIC INVERTEBRATES NSPF	0308900000	0308900000
other aquatic invertebrates	AQUATIC INVERTEBRATES NSPF PREPARED/PRESERVED	1605690000	1605690000
other crustaceans	CRUSTACEANS NSPF DRIED/SALTED/BRINE	0306990000	0306290100
other crustaceans	CRUSTACEANS NSPF FROZEN	0306190061	0306190061
other crustaceans	CRUSTACEANS NSPF LIVE/FRESH	0306390000	0306290100
other crustaceans	CRUSTACEANS NSPF LIVE/FRESH/DRIED/SALTED/BRINE	0306290100	0306290100
other crustaceans	CRUSTACEANS NSPF PREPARED/PRESERVED	1605401090	1605401090
other crustaceans	CRUSTACEANS NSPF PRODUCTS PREPARED DINNERS	1605400500	1605400500
other molluscs	MOLLUSCS NSPF DRIED/SALTED/BRINE	0307990200	0307990100
other molluscs	MOLLUSCS NSPF FROZEN	0307920090	0307990100
other molluscs	MOLLUSCS NSPF FROZEN/DRIED/SALTED/BRINE	0307990100	0307990100
other molluscs	MOLLUSCS NSPF LIVE/FRESH	0307910190	0307910190
other molluscs	MOLLUSCS NSPF LIVE/FRESH	0307910290	0307910190
other molluscs	MOLLUSCS NSPF PREPARED/PRESERVED	1605596000	1605596000
other molluscs	MOLLUSCS NSPF PRODUCTS PREPARED DINNERS	1605590500	1605590500
other shellfish	ANALOG PRODUCT CONTAINING SHELLFISH	1604200590	1604200590
other_crab	CRAB NSPF DRIED/SALTED/BRINE	0306934000	0306244000

other_crab	CRAB NSPF FROZEN	0306144090	0306144090
other_crab	CRAB NSPF LIVE/FRESH	0306334000	0306244000
other_crab	CRAB NSPF LIVE/FRESH/SALTED/BRINE	0306244000	0306244000
other_crab	CRAB NSPF OTHER PREPARATIONS	1605106090	1605106090
other_crab	CRAB NSPF OTHER PREPARATIONS IN ATC	1605106010	1605106010
other_crab	CRAB PRODUCTS PREPARED DINNERS IN ATC	1605100510	1605100510
other_crab	CRAB PRODUCTS PREPARED DINNERS NOT IN ATC	1605100590	1605100590
other_crab	CRABMEAT NSPF DRIED/SALTED/BRINE	0306932000	0306242000
other_crab	CRABMEAT NSPF FRESH	0306332000	0306242000
other_crab	CRABMEAT NSPF FRESH/DRIED/SALTED/BRINE	0306242000	0306242000
other_crab	CRABMEAT NSPF FROZEN	0306142000	0306142000
other_crab	CRABMEAT NSPF FROZEN	1605104035	1605104035
other_crab	CRABMEAT NSPF IN ATC	1605102090	1605102090
other_crab	CRABMEAT NSPF OTHER PREPARATIONS	1605104040	1605104040
other_flatfish	FLATFISH NSPF FILLET BLOCKS FROZEN > 4.5KG	0304831090	0304831090
other_flatfish	FLATFISH NSPF FILLET FRESH	0304430090	0304430090
other_flatfish	FLATFISH NSPF FILLET FROZEN	0304835090	0304835090
other_flatfish	FLATFISH NSPF FRESH	0302290190	0302290190
other_flatfish	FLATFISH NSPF FROZEN	0303390195	0303390195
other_flatfish	FLATFISH NSPF MEAT FRESH	0304590060	0304590060
other_flatfish	FLATFISH NSPF MEAT FRESH	0304590061	0304590060
other_groundfish	GROUNDFISH NSPF DRIED	0305530000	0305320090
other_groundfish	GROUNDFISH NSPF FILLET BLOCKS FROZEN > 4.5KG	0304791090	0304791090
other_groundfish	GROUNDFISH NSPF FILLET DRIED/SALTED/BRINE	0305320090	0305320090
other_groundfish	GROUNDFISH NSPF FILLET FRESH	0304440090	0304440090
other_groundfish	GROUNDFISH NSPF FILLET FROZEN	0304795000	0304795000
other_groundfish	GROUNDFISH NSPF FRESH	0302595090	0302595090
other_groundfish	GROUNDFISH NSPF FRESH NOT >6.8KG	0302591100	0302591100
other_groundfish	GROUNDFISH NSPF FROZEN	0303690000	0303690000
other_groundfish	GROUNDFISH NSPF MEAT FRESH	0304530090	0304530090
other_groundfish	GROUNDFISH NSPF MEAT FROZEN > 6.8KG	0304951090	0304951090
other_groundfish	GROUNDFISH NSPF MEAT FROZEN NOT > 6.8KG	0304959000	0304959000
other_groundfish	GROUNDFISH NSPF MINCED FROZEN > 6.8KG	0304951015	0304951015
other_groundfish	GROUNDFISH NSPF SURIMI	0304951005	0304951005
other_salmon	SALMON FILLET BLOCKS FROZEN > 4.5KG	0304811000	0304811000
other_salmon	SALMON NSPF CANNED IN OIL	1604112090	1604112090
other_salmon	SALMON NSPF CANNED NOT IN OIL	1604114040	1604114040
other_salmon	SALMON NSPF FILLET FRESH	0304410090	0304410090
other_salmon	SALMON NSPF FILLET FROZEN	0304815090	0304815090
other_salmon	SALMON NSPF FRESH	0302130062	0302130062
other_salmon	SALMON NSPF MEAT FRESH	0304520020	0304520020
other_salmon	SALMON NSPF PREPARED/PRESERVED	1604114050	1604114050
other_salmon	SALMON NSPF SALTED	0305694000	0305694000

other_salmon	SALMON PACIFIC NSPF FROZEN	0303120062	0303120062
other_salmon	SALMON SMOKED	0305410000	0305410000
other_salmon	SALMONIDAE NSPF FRESH	0302190000	0302190000
other_salmon	SALMONIDAE NSPF FROZEN	0303190100	0303190100
other_salmon	SALMONIDAE NSPF MEAT FRESH	0304520090	0304520090
other_tuna	TUNA NSPF FILLET FROZEN	0304870000	0304870000
other_tuna	TUNA NSPF FRESH	0302390200	0302390200
other_tuna	TUNA NSPF FROZEN	0303490200	0303490200
other_tuna	TUNA NSPF IN ATC (FOIL OR FLEXIBLE) IN OIL	1604141010	1604141010
other_tuna	TUNA NSPF IN ATC (FOIL OR FLEXIBLE) NOT IN OIL IN QUOTA	1604142291	1604142291
other_tuna	TUNA NSPF IN ATC (FOIL OR FLEXIBLE) NOT IN OIL OVER QUOTA	1604143091	1604143091
other_tuna	TUNA NSPF IN ATC (OTHER) IN OIL	1604141099	1604141099
other_tuna	TUNA NSPF IN ATC (OTHER) NOT IN OIL IN QUOTA	1604142299	1604142299
other_tuna	TUNA NSPF IN ATC (OTHER) NOT IN OIL OVER QUOTA	1604143099	1604143099
other_tuna	TUNA NSPF MEAT FROZEN > 6.8KG	0304991090	0304991090
other_tuna	TUNA NSPF NOT IN A.T.C. NOT IN OIL > 6.8KG	1604144000	1604144000
other_tuna	TUNA NSPF NOT IN A.T.C. NOT IN OIL NOT > 6.8KG	1604145000	1604145000
other_tuna	TUNA (THUNNUS)/SKIPJACK/BONITO MEAT FROZEN > 6.8 kg	0304991190	0304991190
oysters	OYSTERS CANNED	1605515000	1605515000
oysters	OYSTERS CANNED SMOKED	1605514000	1605514000
oysters	OYSTERS DRIED/SALTED/BRINE FARMED	0307190160	0307190060
oysters	OYSTERS DRIED/SALTED/BRINE WILD	0307190180	0307190080
oysters	OYSTERS FROZEN FARMED	0307120060	0307190060
oysters	OYSTERS FROZEN WILD	0307120080	0307190080
oysters	OYSTERS FROZEN/DRIED/SALTED/BRINE FARMED	0307190060	0307190060
oysters	OYSTERS FROZEN/DRIED/SALTED/BRINE WILD	0307190080	0307190080
oysters	OYSTERS LIVE/FRESH FARMED	0307110060	0307110060
oysters	OYSTERS LIVE/FRESH WILD	0307110080	0307110080
oysters	OYSTERS PRODUCTS PREPARED DINNERS	1605510500	1605510500
perch nspf	PERCH NSPF FILLET BLOCKS FROZEN > 4.5KG	0304891035	0304891035
perch nspf	PERCH NSPF FILLET FROZEN	0304895009	0304895009
perch nspf	PERCH NSPF MEAT FRESH	0304590009	0304590009
pickerel	PICKEREL FILLET BLOCKS FROZEN > 4.5KG	0304891015	0304891015
pickerel	PICKEREL FILLET FRESH	0304490006	0304490006
pickerel	PICKEREL FILLET FRESH	0304490106	0304490006
pickerel	PICKEREL FILLET FROZEN	0304895015	0304895015
pickerel	PICKEREL FRESH	0302895010	0302895010
pickerel	PICKEREL MEAT FRESH	0304590020	0304590020
pike	PIKE FILLET FRESH	0304490003	0304490003
pike	PIKE FILLET FRESH	0304490103	0304490003

pike	PIKE FILLET FROZEN	0304895020	0304895020
pike	PIKE FRESH	0302895007	0302895007
pike	PIKE MEAT FRESH	0304590015	0304590015
pike	PIKE MEAT FROZEN > 6.8KG	0304991182	0304991182
pink_salmon	SALMON PINK CANNED IN OIL	1604112020	1604112020
pink_salmon	SALMON PINK CANNED NOT IN OIL	1604114020	1604114020
pink_salmon	SALMON PINK FRESH	0302130032	0302130032
pink_salmon	SALMON PINK FROZEN	0303120032	0303120032
plaice_flatfish	FLATFISH PLAICE FILLET FRESH	0304430015	0304430015
plaice_flatfish	FLATFISH PLAICE FRESH	0302220000	0302220000
plaice_flatfish	FLATFISH PLAICE FROZEN	0303320000	0303320000
plaice_flatfish	FLATFISH PLAICE MEAT FRESH	0304590045	0304590045
pollock_groundfish	GROUNDFISH POLLOCK ALASKA FILLET BLOCKS FROZEN > 4.5KG	0304751000	0304751000
pollock_groundfish	GROUNDFISH POLLOCK ALASKA FILLET FROZEN	0304755000	0304755000
pollock_groundfish	GROUNDFISH POLLOCK ALASKA FRESH	0302555000	0302555000
pollock_groundfish	GROUNDFISH POLLOCK ALASKA FRESH NOT > 6.8KG	0302551100	0302551100
pollock_groundfish	GROUNDFISH POLLOCK ALASKA FROZEN	0303670000	0303670000
pollock_groundfish	GROUNDFISH POLLOCK ALASKA MEAT FROZEN > 6.8KG	0304941090	0304941090
pollock_groundfish	GROUNDFISH POLLOCK ALASKA MEAT FROZEN NOT > 6.8KG	0304949000	0304949000
pollock_groundfish	GROUNDFISH POLLOCK ALASKA MINCED FROZEN > 6.8KG	0304941010	0304941010
pollock_groundfish	GROUNDFISH POLLOCK ALASKA SURIMI	0304941005	0304941005
pollock_groundfish	GROUNDFISH POLLOCK ATLANTIC FILLET BLOCKS FROZEN > 4.5KG	0304731000	0304731000
pollock_groundfish	GROUNDFISH POLLOCK ATLANTIC FILLET FROZEN	0304735000	0304735000
pollock_groundfish	GROUNDFISH POLLOCK ATLANTIC FRESH	0302530000	0302530000
pollock_groundfish	GROUNDFISH POLLOCK ATLANTIC FROZEN	0303650000	0303650000
pollock_groundfish	GROUNDFISH POLLOCK NSPF FILLET BLOCKS FROZEN > 4.5KG	0304791010	0304791010
pollock_groundfish	GROUNDFISH POLLOCK NSPF FILLET FRESH	0304440025	0304440025
pollock_groundfish	GROUNDFISH POLLOCK NSPF FILLET SALTED	0305691042	0305691042
pollock_groundfish	GROUNDFISH POLLOCK NSPF FRESH	0302595010	0302595010
pollock_groundfish	GROUNDFISH POLLOCK NSPF MEAT FRESH	0304530025	0304530025
pollock_groundfish	GROUNDFISH POLLOCK NSPF MEAT FROZEN > 6.8KG	0304951030	0304951030
pollock_groundfish	GROUNDFISH POLLOCK NSPF SALTED WHOLE/DRESSED	0305691022	0305691022
rays skates	RAYS AND SKATES (RAJIDAE) FILLET FRESH	0304480000	0302820000
rays skates	RAYS AND SKATES (RAJIDAE) MEAT FRESH	0304570000	0302820000
rays skates	RAYS AND SKATES (RAJIDAE) MEAT FROZEN	0304970000	0303820000
rays skates	RAYS, SKATES FRESH	0302820000	0302820000
rays skates	RAYS, SKATES FROZEN	0303820000	0303820000

sablefish	SABLEFISH FRESH	0302895052	0302895052
sablefish	SABLEFISH FRESH NOT > 6.8KG	0302891120	0302891120
sablefish	SABLEFISH FROZEN	0303890061	0303890061
sardine	SARDINE CANNED IN OIL NOT SKINNED/BONE	1604132000	1604132000
sardine	SARDINE CANNED IN OIL SKINNED/BONE	1604133000	1604133000
sardine	SARDINE CANNED IN OIL SMOKED NOT SKIN/BONE VALUE > $1/KG	1604131000	1604131000
sardine	SARDINE CANNED NOT IN OIL > 225 GR	1604139000	1604139000
sardine	SARDINE CANNED NOT IN OIL NOT > 225 GR	1604134000	1604134000
sardine	SARDINE,SARDINELLA,BRISLING,SPRAT FRESH	0302430000	0302430000
sardine	SARDINE,SARDINELLA,BRISLING,SPRAT FROZEN	0303530000	0303530000
sauger	SAUGER FILLET BLOCKS FROZEN > 4.5KG	0304891020	0304891020
sauger	SAUGER FILLET FROZEN	0304895025	0304895025
sauger	SAUGER FROZEN	0303890034	0303890034
scallops	SCALLOPS DRIED/SALTED/BRINE	0307290100	0307290000
scallops	SCALLOPS FROZEN	0307220000	0307290000
scallops	SCALLOPS FROZEN/DRIED/SALTED/BRINE	0307290000	0307290000
scallops	SCALLOPS LIVE/FRESH	0307210000	0307210000
scallops	SCALLOPS PREPARED/PRESERVED	1605526000	1605526000
scallops	SCALLOPS PRODUCTS PREPARED DINNERS	1605520500	1605520500
sea bass	SEA BASS (DICENTRARCHUS SPP.) FRESH	0302845000	0302845000
sea bass	SEA BASS (DICENTRARCHUS SPP.) FRESH NOT > 6.8KG	0302841100	0302841100
sea bass	SEA BASS (DICENTRARCHUS SPP.) FROZEN	0303840000	0303840000
sea cucumber	SEA CUCUMBERS DRIED/SALTED/BRINE	0308190100	0308190000
sea cucumber	SEA CUCUMBERS FROZEN	0308120000	0308190000
sea cucumber	SEA CUCUMBERS FROZEN/DRIED/SALTED/BRINE	0308190000	0308190000
sea cucumber	SEA CUCUMBERS LIVE/FRESH	0308110000	0308110000
sea cucumber	SEA CUCUMBERS PREPARED/PRESERVED	1605610000	1605610000
sea urchin	SEA URCHIN DRIED/SALTED/BRINE	0308290100	0308290000
sea urchin	SEA URCHIN FROZEN	0308220000	0308290000
sea urchin	SEA URCHIN FROZEN/DRIED/SALTED/BRINE	0308290000	0308290000
sea urchin	SEA URCHIN LIVE/FRESH	0308210029	0308210029
sea urchin	SEA URCHIN PREPARED/PRESERVED	1605620000	1605620000
seabream	SEABREAM (SPARIDAE) FRESH	0302855000	0302855000
seabream	SEABREAM (SPARIDAE) FRESH NOT > 6.8KG	0302851100	0302851100
shark	DOGFISH AND OTHER SHARK FILLET FRESH	0304470000	0302810011
shark	DOGFISH AND OTHER SHARK MEAT FROZEN	0304960000	0303810010
shark	SHARK DOGFISH FRESH	0302810010	0302810010
shark	SHARK DOGFISH FRESH	0302810011	0302810010
shark	SHARK DOGFISH FROZEN	0303810010	0303810010
shark	SHARK DOGFISH FROZEN	0303810011	0303810010
shark	SHARK FINS	0305710000	0305710000

shark	SHARK FINS FRESH	0302920000	0305710000
shark	SHARK FINS FROZEN	0303920000	0305710000
shark	SHARK FINS PREPARED/PRESERVED	1604189000	0305710000
shark	SHARK NSPF FRESH	0302810090	0302810090
shark	SHARK NSPF FRESH	0302810091	0302810090
shark	SHARK NSPF FROZEN	0303810090	0303810090
shark	SHARK NSPF FROZEN	0303810091	0303810090
shrimp	SHRIMP BREADED FROZEN	1605211020	1605211020
shrimp	SHRIMP CANNED	1605291040	1605291040
shrimp	SHRIMP COLD-WATER PEELED FRESH	0306350040	0306260040
shrimp	SHRIMP COLD-WATER PEELED FRESH/DRIED/SALTED/BRINE	0306260040	0306260040
shrimp	SHRIMP COLD-WATER PEELED FROZEN	0306160040	0306160040
shrimp	SHRIMP COLD-WATER SHELL-ON FRESH	0306350020	0306260020
shrimp	SHRIMP COLD-WATER SHELL-ON FRESH/DRIED/SALTED/BRINE	0306260020	0306260020
shrimp	SHRIMP COLD-WATER SHELL-ON FROZEN 15/20	0306160006	0306160006
shrimp	SHRIMP COLD-WATER SHELL-ON FROZEN 21/25	0306160009	0306160009
shrimp	SHRIMP COLD-WATER SHELL-ON FROZEN 26/30	0306160012	0306160012
shrimp	SHRIMP COLD-WATER SHELL-ON FROZEN 31/40	0306160015	0306160015
shrimp	SHRIMP COLD-WATER SHELL-ON FROZEN 41/50	0306160018	0306160018
shrimp	SHRIMP COLD-WATER SHELL-ON FROZEN 51/60	0306160021	0306160021
shrimp	SHRIMP COLD-WATER SHELL-ON FROZEN 61/70	0306160024	0306160024
shrimp	SHRIMP COLD-WATER SHELL-ON FROZEN < 15	0306160003	0306160003
shrimp	SHRIMP COLD-WATER SHELL-ON FROZEN > 70	0306160027	0306160027
shrimp	SHRIMP FROZEN IN ATC	1605291010	1605291010
shrimp	SHRIMP FROZEN OTHER PREPARATIONS	1605211030	1605211030
shrimp	SHRIMP OTHER PREPARATIONS	1605211050	1605211050
shrimp	SHRIMP PEELED DRIED/SALTED/BRINE	0306950040	0306270040, 0306260040
shrimp	SHRIMP PRODUCTS PREPARED DINNERS IN ATC	1605290500	1605290500
shrimp	SHRIMP PRODUCTS PREPARED DINNERS NOT IN ATC	1605210500	1605210500
shrimp	SHRIMP SHELL-ON DRIED/SALTED/BRINE	0306950020	0306260020, 0306270020
shrimp	SHRIMP WARM-WATER PEELED FRESH	0306360040	0306270040
shrimp	SHRIMP WARM-WATER PEELED FRESH/DRIED/SALTED/BRINE	0306270040	0306270040
shrimp	SHRIMP WARM-WATER PEELED FROZEN	0306170040	0306170040
shrimp	SHRIMP WARM-WATER SHELL-ON FRESH	0306360020	0306270020
shrimp	SHRIMP WARM-WATER SHELL-ON FRESH/DRIED/SALTED/BRINE	0306270020	0306270020
shrimp	SHRIMP WARM-WATER SHELL-ON FROZEN 15/20	0306170006	0306170006
shrimp	SHRIMP WARM-WATER SHELL-ON FROZEN 21/25	0306170009	0306170009
shrimp	SHRIMP WARM-WATER SHELL-ON FROZEN 26/30	0306170012	0306170012

shrimp	SHRIMP WARM-WATER SHELL-ON FROZEN 31/40	0306170015	0306170015
shrimp	SHRIMP WARM-WATER SHELL-ON FROZEN 41/50	0306170018	0306170018
shrimp	SHRIMP WARM-WATER SHELL-ON FROZEN 51/60	0306170021	0306170021
shrimp	SHRIMP WARM-WATER SHELL-ON FROZEN 61/70	0306170024	0306170024
shrimp	SHRIMP WARM-WATER SHELL-ON FROZEN < 15	0306170003	0306170003
shrimp	SHRIMP WARM-WATER SHELL-ON FROZEN > 70	0306170027	0306170027
skipjack_tuna	TUNA SKIPJACK FRESH	0302330000	0302330000
skipjack_tuna	TUNA SKIPJACK FROZEN	0303430000	0303430000
smelts	SMELTS NSPF FRESH	0302895037	0302895037
smelts	SMELTS NSPF FROZEN	0303890004	0303890004
smelts	SMELTS SEA FROZEN	0303890001	0303890001
snail	SNAIL OTHER THAN SEA SNAIL LIVE/FRESH/FROZEN/SALTED	0307600000	0307600000
snail	SNAIL OTHER THAN SEA SNAIL PREPARED/PRESERVED	1605585500	1605585500
snail	SNAIL OTHER THAN SEA SNAIL PRODUCTS PREPARED DINNERS	1605580500	1605580500
snapper	SNAPPER (LUTJANIDAE SPP.) FRESH	0302895058	0302895058
snapper	SNAPPER (LUTJANIDAE SPP.) FROZEN	0303890067	0303890067
snow_crab	CRAB SNOW FROZEN	0306144020	0306144020
snow_crab	CRABMEAT SNOW (OPILIO) FROZEN	1605104005	1605104005
snow_crab	CRABMEAT SNOW (OPILIO) IN ATC	1605102022	1605102022
snow_crab	CRABMEAT SNOW OTHER FROZEN	1605104010	1605104010
snow_crab	CRABMEAT SNOW OTHER IN ATC	1605102025	1605102025
sockeye_salmon	SALMON SOCKEYE CANNED IN OIL	1604112030	1604112030
sockeye_salmon	SALMON SOCKEYE CANNED NOT IN OIL	1604114030	1604114030
sockeye_salmon	SALMON SOCKEYE FRESH	0302130042	0302130042
sockeye_salmon	SALMON SOCKEYE FROZEN	0303110000	0303110000
sole_flatfish	FLATFISH SOLE NSPF MEAT FRESH	0304590040	0304590040
sole_flatfish	FLATFISH SOLE (SOLEA SPP) FRESH	0302230000	0302230000
sole_flatfish	FLATFISH SOLE (SOLEA SPP) FROZEN	0303330000	0303330000
sole_flatfish	FLATFISH SOLE NSPF FILLET BLOCKS FROZEN > 4.5KG	0304831015	0304831015
sole_flatfish	FLATFISH SOLE NSPF FILLET FRESH	0304430010	0304430010
sole_flatfish	FLATFISH SOLE NSPF FILLET FROZEN	0304835015	0304835015
sole_flatfish	FLATFISH SOLE NSPF MEAT FROZEN > 6.8KG	0304991150	0304991150
sole_flatfish	FLATFISH SOLE ROCK FROZEN	0303390120	0303390120
sole_flatfish	FLATFISH SOLE YELLOWFIN FROZEN	0303390130	0303390130
species_unidentified	CARP,CATFISH,EELS,NILE PERCH,SNAKEHEAD,TILAPIA DRIED	0305520000	0305310000
species_unidentified	CARP,CATFISH,EELS,NILE PERCH,SNAKEHEAD,TILAPIA FILLET DRIED/SALTED/BRINE	0305310000	0305310000
species_unidentified	CARP,CATFISH,EELS,NILE PERCH,SNAKEHEAD,TILAPIA FILLET DRIED/SALTED/BRINE	0305310100	0305310000

species_unidentified	CARP,CATFISH,EELS,NILE PERCH,SNAKEHEAD,TILAPIA SALTED > 6.8 KG	0305645000	0305645000
species_unidentified	CARP,CATFISH,EELS,NILE PERCH,SNAKEHEAD,TILAPIA SALTED NOT > 6.8KG	0305641000	0305641000
species_unidentified	CARP,CATFISH,EELS,NILE PERCH,SNAKEHEAD,TILAPIA SMOKED	0305440000	0305440000
species_unidentified	CARP,CATFISH,EELS,NILE PERCH,SNAKEHEAD,TILAPIA SMOKED	0305440100	0305440000
species_unidentified	CARP,CATFISH,EELS,SNAKEHEAD MEAT FRESH	0304510090	0304510090
species_unidentified	CARP,CATFISH,EELS,SNAKEHEAD MEAT FRESH	0304510190	0304510090
species_unidentified	CARP,CATFISH,EELS,SNAKEHEAD,TILAPIA FROZEN NOT > 6.8KG	0304939000	0304939000
species_unidentified	CARP,CATFISH,EELS,SNAKEHEAD,TILAPIA MEAT FROZEN > 6.8KG	0304931090	0304931090
species_unidentified	CARP,CATFISH,EELS,SNAKEHEAD,TILAPIA MINCED FROZEN > 6.8KG	0304931010	0304931010
species_unidentified	CARP,CATFISH,EELS,SNAKEHEAD,TILAPIA SURIMI	0304931005	0304931005
species_unidentified	CARP,EELS,SNAKEHEAD FILLET FRESH	0304390000	0304390000
species_unidentified	CARP,EELS,SNAKEHEAD FILLET FROZEN	0304690000	0304690000
species_unidentified	CLAM,COCKLES,ARK SHELLS NSPF PRODUCTS PREPARED DINNERS	1605560500	1605560500
species_unidentified	CUTTLEFISH,SQUID PRODUCTS PREPARED DINNERS	1605540500	1605540500
species_unidentified	DOGFISH, OTHER SHARK, RAYS AND SKATES (RAJIDAE) FILLET FROZEN	0304880000	0303810010
species_unidentified	FISH NSPF DRIED	0305590000	0305590000
species_unidentified	FISH NSPF DRIED	0305590001	0305590000
species_unidentified	FISH NSPF FILLET BLOCKS FROZEN > 4.5KG	0304891090	0304891090
species_unidentified	FISH NSPF FILLET DRIED/SALTED/BRINE	0305396080	0305396080
species_unidentified	FISH NSPF FILLET DRIED/SALTED/BRINE	0305396180	0305396080
species_unidentified	FISH NSPF FRESH SCALED NOT >6.8KG	0302891140	0302891140
species_unidentified	FISH NSPF HEADS,TAILS,MAWS DRIED/SALTED/BRINE/SMOKED	0305720000	0305720000
species_unidentified	FISH NSPF IN ATC IN OIL	1604193100	1604193100
species_unidentified	FISH NSPF IN ATC IN OIL	1604193200	1604193100
species_unidentified	FISH NSPF IN ATC NOT IN OIL	1604192100	1604192100
species_unidentified	FISH NSPF IN ATC NOT IN OIL	1604192200	1604192100
species_unidentified	FISH NSPF MINCED FROZEN > 6.8KG	0304991109	0304991109
species_unidentified	FISH NSPF PREPARED DINNERS CONTAINING SHELLFISH	1604200510	1604200510
species_unidentified	FISH NSPF SALTED > 6.8KG	0305696001	0305696001
species_unidentified	FISH NSPF SALTED NOT > 6.8KG	0305695001	0305695001
species_unidentified	FISH NSPF SMOKED	0305494041	0305494041
species_unidentified	FISH NSPF SMOKED	0305494045	0305494041
species_unidentified	FISH NSPF SURIMI	0304991104	0304991104
species_unidentified	FRESHWATER FISH NSPF FILLET FRESH	0304490015	0304490015

species_unidentified	FRESHWATER FISH NSPF FILLET FRESH	0304490115	0304490015
species_unidentified	FRESHWATER FISH NSPF FILLET FROZEN	0304895030	0304895030
species_unidentified	FRESHWATER FISH NSPF FRESH	0302795076	0302795076
species_unidentified	FRESHWATER FISH NSPF FRESH	0302895034	0302895034
species_unidentified	FRESHWATER FISH NSPF FRESH SCALED, IN CONTAINER NOT >6.8KG	0302791100	0302791100
species_unidentified	FRESHWATER FISH NSPF FROZEN	0303290150	0303290150
species_unidentified	FRESHWATER FISH NSPF FROZEN	0303890043	0303890043
species_unidentified	FRESHWATER FISH NSPF MEAT FRESH	0304590035	0304590035
species_unidentified	FRESHWATER FISH NSPF MEAT FRESH	0304590036	0304590035
species_unidentified	FRESHWATER FISH NSPF MEAT FROZEN > 6.8KG	0304991184	0304991184
species_unidentified	FRESHWATER FISH NSPF MEAT FROZEN NOT > 6.8KG	0304999192	0304999192
species_unidentified	HERRING, ANCHOVY, SARDINE, SPRAT, MACKEREL, INDIAN MACKEREL, SEERFISH, JACK AND HORSE MACKEREL, JACKS, CREVALLES, COBIA, SILVER POMFRETS, PACIFIC SAURY, SCAD, CAPELIN, SWORDFISH, KAWAKAWA, BONITO, MARLIN, SAILFISH, SPEARFISH DRIED	0305540000	0305590000
species_unidentified	MARINE FISH NSPF FILLET FRESH	0304490090	0304490090
species_unidentified	MARINE FISH NSPF FILLET FRESH	0304490190	0304490090
species_unidentified	MARINE FISH NSPF FILLET FROZEN	0304895090	0304895090
species_unidentified	MARINE FISH NSPF FILLET FROZEN	0304895091	0304895090
species_unidentified	MARINE FISH NSPF FRESH	0302490000	0302895076
species_unidentified	MARINE FISH NSPF FRESH	0302895076	0302895076
species_unidentified	MARINE FISH NSPF FRESH	0302895077	0302895076
species_unidentified	MARINE FISH NSPF FROZEN	0303590000	0303890079
species_unidentified	MARINE FISH NSPF FROZEN	0303890079	0303890079
species_unidentified	MARINE FISH NSPF FROZEN	0303890080	0303890079
species_unidentified	MARINE FISH NSPF MEAT FRESH	0304590090	0304590090
species_unidentified	MARINE FISH NSPF MEAT FRESH	0304590091	0304590090
species_unidentified	MARINE FISH NSPF MEAT FROZEN > 6.8KG	0304991194	0304991194
species_unidentified	MARINE FISH NSPF MEAT FROZEN NOT > 6.8 KG	0304999190	0304999191
species_unidentified	MARINE FISH NSPF MEAT FROZEN NOT > 6.8 KG	0304999191	0304999191
species_unidentified	PERCH FRESHWATER NSPF FRESH	0302895025	0302895025
species_unidentified	PERCH,PIKE PERCH,YELLOW PIKE FROZEN	0303890031	0303890031
species_unidentified	PIKE PERCH,YELLOW PIKE FILLET FROZEN	0304895006	0304895006
species_unidentified	PIKE PERCH,YELLOW PIKE FRESH	0302895013	0302895013
species_unidentified	PIKE PERCH,YELLOW PIKE MEAT FRESH	0304590006	0304590006
species_unidentified	PIKE,PICKEREL FROZEN	0303890028	0303890028
species_unidentified	SHAD,STURGEON FRESH	0302895049	0302895049
species_unidentified	SHAD,STURGEON FROZEN	0303890013	0303890013
squid	SQUID (LOLIGO NSPF) DRIED/SALTED/BRINE	0307490129	0307490029
squid	SQUID (LOLIGO NSPF) FROZEN	0307430029	0307490029
squid	SQUID (LOLIGO NSPF) FROZEN/DRIED/SALTED/BRINE	0307490029	0307490029

squid	SQUID (LOLIGO NSPF) LIVE/FRESH	0307410020	0307410020
squid	SQUID (LOLIGO NSPF) PREPARED/PRESERVED	1605546020	1605546020
squid	SQUID (LOLIGO OPALESCENS) FROZEN	0307430022	0307490022
squid	SQUID (LOLIGO OPALESCENS) FROZEN/DRIED/SALTED/BRINE	0307490022	0307490022
squid	SQUID (LOLIGO OPALESCENS)DRIED/SALTED/BRINE	0307490122	0307490022
squid	SQUID (LOLIGO PEALEI) DRIED/SALTED/BRINE	0307490124	0307490024
squid	SQUID (LOLIGO PEALEI) FROZEN	0307430024	0307490024
squid	SQUID (LOLIGO PEALEI) FROZEN/DRIED/SALTED/BRINE	0307490024	0307490024
squid	SQUID (LOLIGO SPP.) LIVE/FRESH	0307420020	0307410020
squid	SQUID FILLET FROZEN	0307430010	0307490010
squid	SQUID NSPF DRIED/SALTED/BRINE	0307490150	0307490050
squid	SQUID NSPF FILLET FROZEN	0307490010	0307490010
squid	SQUID NSPF FROZEN	0307430050	0307490050
squid	SQUID NSPF FROZEN/DRIED/SALTED/BRINE	0307490050	0307490050
squid	SQUID NSPF LIVE/FRESH	0307410040	0307410040
squid	SQUID NSPF LIVE/FRESH	0307420040	0307410040
squid	SQUID NSPF PREPARED/PRESERVED	1605546030	1605546030
swimming_crab	CRABMEAT SWIMMING (PORTUNIDAE) FROZEN	1605104030	1605104030
swimming_crab	CRABMEAT SWIMMING (PORTUNIDAE) IN ATC	1605102059	1605102059
swordfish	SWORDFISH FILLET FRESH	0304450000	0304450000
swordfish	SWORDFISH FILLET FROZEN	0304840000	0304840000
swordfish	SWORDFISH FRESH	0302470090	0302470090
swordfish	SWORDFISH FROZEN	0303570090	0303570090
swordfish	SWORDFISH MEAT FRESH	0304540000	0304540000
swordfish	SWORDFISH MEAT FROZEN > 6.8KG	0304911000	0304911000
swordfish	SWORDFISH MEAT FROZEN NOT > 6.8KG	0304919000	0304919000
swordfish	SWORDFISH STEAKS FRESH	0302470010	0302470010
swordfish	SWORDFISH STEAKS FROZEN	0303570010	0303570010
tilapia	TILAPIA (OREOCHROMIS SPP.) FILLET FRESH	0304310000	0304310000
tilapia	TILAPIA (OREOCHROMIS SPP.) FILLET FROZEN	0304610000	0304610000
tilapia	TILAPIA (OREOCHROMIS SPP.) FRESH	0302715000	0302715000
tilapia	TILAPIA (OREOCHROMIS SPP.) FRESH NOT >6.8KG	0302711100	0302711100
tilapia	TILAPIA (OREOCHROMIS SPP.) FROZEN	0303230000	0303230000
tilapia	TILAPIA (OREOCHROMIS SPP.) MEAT FRESH	0304510025	0304510025
tilapia	TILAPIA (OREOCHROMIS SPP.) MEAT FRESH	0304510125	0304510025
tilapia	TILAPIA NSPF FILLET FRESH	0304490012	0304490012
tilapia	TILAPIA NSPF FILLET FRESH	0304490112	0304490012
tilapia	TILAPIA NSPF FROZEN	0303890040	0303890040
tilapia	TILAPIA NSPF MEAT FRESH	0304590030	0304590030
toothfish	TOOTHFISH NSPF FILLET FRESH	0304460000	0304460000
toothfish	TOOTHFISH NSPF FILLET FROZEN	0304850000	0304850000

toothfish	TOOTHFISH NSPF FRESH	0302830000	0302830000
toothfish	TOOTHFISH NSPF FROZEN	0303830000	0303830000
toothfish	TOOTHFISH NSPF MEAT FRESH	0304550000	0304550000
toothfish	TOOTHFISH NSPF MEAT FROZEN > 6.8KG	0304921000	0304921000
toothfish	TOOTHFISH NSPF MEAT FROZEN NOT > 6.8KG	0304929000	0304929000
trout	TROUT NSPF FILLET BLOCKS FROZEN > 4.5KG	0304821000	0304821000
trout	TROUT NSPF FILLET FRESH	0304420000	0304420000
trout	TROUT NSPF FILLET FROZEN	0304825000	0304825000
trout	TROUT NSPF FRESH	0302110090	0302110090
trout	TROUT NSPF FROZEN	0303140000	0303140000
trout	TROUT NSPF SMOKED	0305430000	0305430000
trout	TROUT RAINBOW FRESH FARMED	0302110010	0302110010
turbot_flatfish	FLATFISH TURBOT (PSETTA MAXIMA) FRESH	0302240000	0302240000
turbot_flatfish	FLATFISH TURBOT (PSETTA MAXIMA) FROZEN	0303340000	0303340000
turbot_flatfish	FLATFISH TURBOT GREENLAND FILLET BLOCKS FROZEN > 4.5KG	0304831010	0304831010
turbot_flatfish	FLATFISH TURBOT GREENLAND FILLET FRESH	0304430025	0304430025
turbot_flatfish	FLATFISH TURBOT GREENLAND FILLET FROZEN	0304835010	0304835010
turbot_flatfish	FLATFISH TURBOT GREENLAND FRESH	0302210090	0302210090
turbot_flatfish	FLATFISH TURBOT GREENLAND FROZEN	0303310030	0303310030
turbot_flatfish	FLATFISH TURBOT GREENLAND MEAT FRESH	0304590055	0304590055
turbot_flatfish	FLATFISH TURBOT GREENLAND MEAT FROZEN > 6.8KG	0304991160	0304991160
turbot_flatfish	FLATFISH TURBOT NSPF FILLET BLOCKS FROZEN > 4.5KG	0304831030	0304831030
turbot_flatfish	FLATFISH TURBOT NSPF FILLET FROZEN	0304835030	0304835030
whitefish	WHITEFISH FILLET FRESH	0304490009	0304490009
whitefish	WHITEFISH FILLET FRESH	0304490109	0304490009
whitefish	WHITEFISH FRESH	0302895031	0302895031
whitefish	WHITEFISH FROZEN	0303890037	0303890037
whitefish	WHITEFISH MEAT FRESH	0304590025	0304590025
whitefish	WHITEFISH MEAT FROZEN > 6.8KG	0304991183	0304991183
whiting_groundfish	GROUNDFISH WHITING FILLET BLOCKS FROZEN > 4.5KG	0304791015	0304791015
wolffish	WOLFFISH FILLET BLOCKS FROZEN > 4.5KG	0304891010	0304891010
wolffish	WOLFFISH FILLET FROZEN	0304895045	0304895045
yellow perch	YELLOW PERCH FILLET FROZEN	0304895003	0304895003
yellow perch	YELLOW PERCH MEAT FRESH	0304590003	0304590003
yellowfin_tuna	TUNA YELLOWFIN EVISCERATED HEAD-OFF FROZEN	0303420060	0303420060
yellowfin_tuna	TUNA YELLOWFIN EVISCERATED HEAD-ON FROZEN	0303420040	0303420040
yellowfin_tuna	TUNA YELLOWFIN FRESH	0302320000	0302320000
yellowfin_tuna	TUNA YELLOWFIN WHOLE FROZEN	0303420020	0303420020

References

Breheny, P., n.d. MCMC Diagnostics. BST 701, Bayesian modeling in biostatistics, http://web.as.uky.edu/statistics/users/pbreheny/701/s13/notes/3-5.pdf.

European Market Observatory for Fisheries and Aquaculture Products (EUMOFA). 2021. “Data management Annex 7: Conversion factors by CN8 code.” Web. Retrieved July 11, 2023. https://www.eumofa.eu/supply-balance-and-other-methodologies

Food and Agriculture Organization of the United Nations (FAO). 2023. “FishstatJ: Global production by production source,” Web. Retrieved July 12, 2023. https://www.fao.org/fishery/en/topic/166235?lang=en

Gelman, A., Jakulin, A., Pittau, M.G., Su, Y.S. 2008. A weakly informative default prior distribution for logistic and other regression models, The Annals of Applied Statistics 2(4) 1360-1383.

Gephart, JA, Bejarano, RA, Gorospe, K, Godwin, A, Golden, CD, Naylor, RL, Nash, KL, Pace, ML and Troell, M (In Press). Globalization of wild capture and farmed aquatic foods. Nature Communications.

Kroetz, K., Luque, G.M., Gephart, J.A., Jardine, S.L., Lee, P., Chicojay Moore, K. Cole, C., Steinkruger,A. and C.J. Donlan. 2020. Consequences of seafood mislabeling for marine populations and fisheries management, Proc. Natl. Acad. Sci. 117: 30318–30323. https://doi.org/10.1073/pnas.2003741117.

Kruschke, J. 2015. Doing Bayesian data analysis: A tutorial with R, JAGS, and Stan, Academic Press.

Love DC, Nussbaumer EM, Harding J, Gephart JA, Anderson JL, Asche F, Stoll JS, Thorne-Lyman AL, Bloem MW. 2021. Risks shift along seafood supply chains. Global Food Security. 1;28:100476.

Luque, G. and C.J. Donlan. 2019. The characterization of seafood mislabeling: A global meta-analysis, Biol. Conserv. https://doi.org/https://doi.org/10.1016/j.biocon.2019.04.006.

Macfadyen, G. and Hosch, G. 2023. The IUU Fishing Risk Index: 2023 Update. Poseidon Aquatic Resource Management Limited and the Global Initiative Against Transnational Organized Crime (www.iuufishingindex.net). Accessed May 5, 2024.

National Marine Fisheries Service (NMFS). 2016a. Magnuson-Stevens Fishery Conservation and Management Act; Seafood Import Monitoring Program (Proposed Rule), 81 FR 18558.

National Marine Fisheries Service (NMFS). 2016b. Magnuson-Stevens Fishery Conservation and Management Act; Seafood Import Monitoring Program (Final Rule), 81 FR 88975.

National Marine Fisheries Service (NMFS). 2019. Compliance Guide: U.S. Seafood Import Monitoring Program. https://www.fisheries.noaa.gov/resource/document/compliance-guide-seafood-import-monitoring-program

National Marine Fisheries Service (NMFS). N.d. Foreign Fishery Trade Data. https://www.fisheries.noaa.gov/national/sustainable-fisheries/foreign-fishery-trade-data. Retrieved July 11-12, 2023.

National Oceanic and Atmospheric Administration (NOAA). 2016. “Final rule to implement U.S. Seafood Import Monitoring Program, RIN 0648-BF09. Final regulatory impact review and final regulatory flexibility analysis.”

National Oceanic and Atmospheric Administration (NOAA). 2021. “Efforts to prevent importation of seafood harvested through illegal, unreported, and unregulated fishing and address imported seafood fraud.” https://media.fisheries.noaa.gov/2021-08/SIMP%20Report%20to%20Congress_Efforts%20to%20Prevent%20Seafood%20Harvested%20through%20IUU%20fishing.pdf. Last accessed August 16, 2024.

National Oceanic and Atmospheric Administration (NOAA). 2022. “Developing a priority list of species for consideration under the Seafood Import Monitoring Program.” https://media.fisheries.noaa.gov/2022-03/NOAA%20NMFS-Seafood%20Import%20Monitoring%20Program%20Priority%20List%20Report.pdf. Last

accessed August 16, 2024
National Oceanic and Atmospheric Administration, National Marine Fisheries Service (NOAA Fisheries). 2019. “Three Alpha Codes for Seafood Import Monitoring Program.” Web. Retrieved June 22, 2023. https://www.fisheries.noaa.gov/resource/form/three-alpha-codes-seafood-import-monitoring-program
National Oceanic and Atmospheric Administration, National Marine Fisheries Service (NOAA Fisheries). 2024. “Report to Congress: Report on the Seafood Import Monitoring Program – FY 2023.” Available: https://www.fisheries.noaa.gov/s3/2024-05/SIMP-Report-to-Congress-FY2023.pdf. Last accessed August 14, 2024.
Plummer, M. 2017. JAGS Version 4.3.0 user manual, https://sourceforge.net/projects/mcmc-jags/files/Manuals/4.x/jags_user_manual.pdf/download.
Poseidon Aquatic Resource Management Ltd (Poseidon). 2024. Methodology for IUU Fishing Index. Available: https://iuufishingindex.net/methodology.pdf. Accessed May 5, 2024.
"Presidential Task Force on Combating Illegal Unreported and Unregulated Fishing and Seafood Fraud Action Plan (Notice; request for comments)." Federal Register 80:148 (August 3, 2015) pp. 45955-45963.
R Development Core Team. 2017. R: A Language and Environment for Statistical Computing. The R Foundation for Statistical Computing, Available online at http://www.R-project.org, Vienna, Austria.
U.S. International Trade Commission (USITC). 2021. Seafood Obtained via Illegal, Unreported, and Unregulated Fishing: U.S. Imports and Economic Impact on U.S. Commercial Fisheries. USITC Publication 5168. Washington, DC: USITC, March 2021. https://usitc.gov/publications/332/pub5168.pdf.
U.S. International Trade Commission (USITC). 2023. Harmonized tariff Schedule. Web. Accessed August 8, 2023. https://hts.usitc.gov
